# *N-*salicyloyl tryptamine derivatives as potential therapeutic agents for doxorubicin-induced hepatotoxicity *via* PI3K/AKT/HO-1 signaling pathway

**DOI:** 10.3389/fphar.2026.1929788

**Published:** 2026-09-24

**Authors:** Yuting Zhao, Jing Zeng, Guanglong Pang, Ying Wu, Shuzhi Wang, Zhengwen Lei, Xiangyong Hao, Yaxin Chen, Chengxuan Hu, Xianlin Zeng, Xinyan Huang, Xinlin Zhong, Zhen Wang, Xue Peng

**Affiliations:** 1 School of Basic Medicine, School of Pharmaceutical Science, Hengyang Medical School, University of South China, Hengyang, Hunan, China; 2 The First Clinical Medical College of Gansu University of Chinese Medicine, Gansu Provincial Hospital, Lanzhou, China; 3 Department of General Surgery, Gansu Provincial Hospital, Lanzhou, China; 4 Department of Thoracic and Cardiovascular Surgery, The First Affiliated Hospital, Hengyang Medical School, University of South China, Hengyang, Hunan, China; 5 Hengyang Traditional Chinese Medicine Hospital, Hengyang, Hunan, China; 6 Department of Oncology, The Affiliated Nanhua Hospital, Hengyang Medical School, University of South China, Hengyang, Hunan, China; 7 Qinghai Provincial Key Laboratory of Tibetan Medicine Research, Northwest Institute of Plateau Biology, Chinese Academy of Sciences, Xining, Qinghai, China; 8 National Health Commission Key Laboratory of Birth Defect Research and Prevention Hunan Provincial Maternal and Child Healthcare Hospital, Changsha, Hunan, China

**Keywords:** DOX-induced hepatotoxicity, doxorubicin, *N*-salicyloyl tryptamine derivatives, oxidative stress, PI3K/AKT/HO-1

## Abstract

Doxorubicin (DOX) is a broad-spectrum antineoplastic agent with remarkable efficacy against various malignant tumors. However, its clinical application is severely limited by profound hepatotoxicity, wherein oxidative stress acts as a pivotal mechanism underlying DOX-induced hepatotoxicity. *N*-salicyloyl tryptamine derivatives (NSTs) have been reported to possess potent antioxidant activities. In the present study, NSTs were applied for the first time in DOX-induced hepatotoxicity with structural modifications to the scaffold. Through systematic screening, a novel compound **A50** was identified as the most potent active candidate. Both in vitro (AML12 cell lines) and in vivo (mice model of DOX-induced hepatotoxicity) experiments demonstrated that **A50** significantly enhanced superoxide dismutase (SOD) activity, reduced malondialdehyde (MDA) levels, stabilized mitochondrial membrane potential (MMP), and promoted adenosine triphosphate (ATP) production, thus effectively improving mitochondrial function and suppressing oxidative stress-mediated damage. Additionally, **A50** decreased serum levels of liver injury biomarkers [alanine transaminase (ALT), aspartate transaminase (AST) and levels of proinflammatory cytokines [tumor necrosis factor-αα (TNF-α), interleukin-6 (IL-6)], inhibited lactate dehydrogenase (LDH) activity, and attenuated histopathological lesions in liver tissues, without affecting its anti-hepatocellular carcinoma activity. Mechanism investigations revealed the hepatoprotective effects of **A50** were associated with activation of the PI3K/AKT/HO-1 signaling pathway, and these effects could be blocked by the PI3K-specific inhibitor LY294002 in vitro. In conclusion, **A50** may alleviated DOX-induced hepatotoxicity by activating the PI3K/AKT/HO-1 pathway to inhibit oxidative stress-driven pathological processes, including mitochondrial dysfunction, inflammation, and apoptosis. These findings highlight **A50** as a promising candidate for the treatment of DOX-induced hepatotoxicity.

## Graphical Abstract

**Figure:**
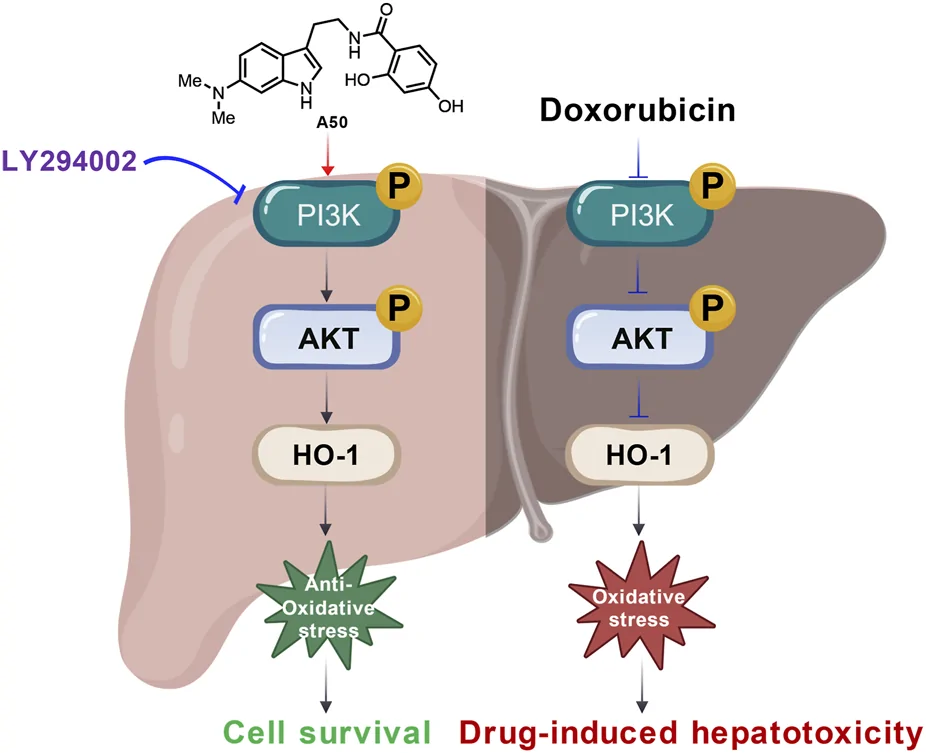

## 1 Introduction

Chemotherapy remains one of the primary therapeutic modalities in contemporary oncological practice and plays a pivotal role in the clinical management of various malignant tumors. It is estimated that approximately 70% of cancer patients undergo chemotherapy at some stage during their treatment course (Walker et al., 2020). Among chemotherapeutic agents, doxorubicin (DOX), a hydroxylated derivative of daunorubicin, is widely used in the treatment of hematologic malignancies (e.g., leukemia and lymphoma), as well as solid tumors including lung, breast, colon, and thyroid cancers (Renu et al., 2019; Sohail et al., 2022). Despite its potent efficacy in inducing cancer cell death, DOX inevitably elicits damage to normal tissues and organs, resulting in multi-system toxicity involving the liver, heart, nervous system, and kidneys, which significantly limits its clinical application (Pugazhendhi et al., 2018; Szponar et al., 2024). Notably, severe DOX-induced adverse effects are associated with an extremely poor prognosis, with a reported mortality rate as high as 48%. Accumulating evidence indicates that the liver, as the primary organ responsible for detoxification and drug metabolism, is particularly susceptible to DOX-induced injury (Zhou et al., 2022). Clinical data show that approximately 40% of patients receiving DOX-based chemotherapy develop hepatotoxicity (Afsar et al., 2019; Kalender et al., 2005). Collectively, these pathological processes compromise the structural and functional integrity of hepatocytes, thereby increasing the risk of hepatic fibrosis and even liver failure (Chen et al., 2016; Martinel Lamas et al., 2015).

In the early stage of DOX-induced hepatotoxicity, oxidative stress emerges as the primary manifestation (Alherz et al., 2023; Huang et al., 2024). The underlying mechanism is that DOX, characterized by its anthraquinone core structure, can be metabolized into unstable semiquinone intermediates. Through redox cycling, these intermediates inactivate endogenous antioxidant defense systems such as superoxide dismutase (SOD)—thereby triggering an explosive burst of reactive oxygen species (ROS). Excessive ROS tightly bind to organelles and cell membranes, causing oxidative damage to lipids, proteins and DNA. Consequently, these lead to cell death, oxidative organ damage and increasing the risk of liver fibrosis and even liver failure (Li et al., 2025; Prasanna et al., 2020). Currently, therapeutic strategies for alleviating DOX-induced hepatotoxicity primarily depend on pharmacological interventions, including iron chelators (e.g., deferoxamine), antioxidants (e.g., vitamin C, vitamin E, *N*-acetylcysteine and silybin) and anti-inflammatory agents (e.g., ibuprofen and dexamethasone) (Chen et al., 2025; Li et al., 2006; Yeh et al., 2007). However, these existing agents were unable to systematically attenuate DOX-triggered oxidative stress and the subsequent downstream pathological cascade reactions, resulting in limited hepatoprotective efficacy (Vickers et al., 2017).

The phosphoinositide 3-kinase/protein kinase B (PI3K/AKT) signaling pathway plays a pivotal role in the pathogenesis of DOX-induced hepatotoxicity. The underlying mechanism is that DOX inhibits AKT activation by promoting the dephosphorylation of phosphatidylinositol 3,4,5-trisphosphate (PIP3), thereby affecting hepatocyte survival, proliferation and metabolism while inducing oxidative stress damage (Alwaili et al., 2025; Liu et al., 2021; Saleh et al., 2022). Accumulating studies have confirmed that DOX significantly reduces the phosphorylation levels of PI3K and AKT in liver tissues (Fawzy et al., 2025; Qin et al., 2022). Conversely, activation of the PI3K/AKT pathway upregulates the expression of multiple antioxidant proteins, thereby exerting prominent antioxidant and anti-inflammatory effects (Ding et al., 2022). Among these, heme oxygenase-1 (HO-1), as the rate-limiting enzyme in heme catabolism, catalyzes the conversion of heme into carbon monoxide (CO), free iron and biliverdin. Biliverdin can further transform into the potent antioxidant bilirubin, serving as a central component in hepatocyte antioxidant defense (Jun et al., 2014). Recent evidence indicates that syringic acid, a classic phenolic acid derivative, activates the PI3K/AKT pathway and significantly upregulates HO-1 expression, thereby effectively mitigating DOX-induced oxidative stress and inflammatory responses in liver tissues and improving liver dysfunction (Jun et al., 2014). Similarly, ginsenoside Rg1 enhances HO-1 levels by increasing the phosphorylation of PI3K (p-PI3K) and phosphorylation of AKT (p-AKT), which in turn inhibits DOX-induced hepatocyte apoptosis and alleviates hepatic pathological damage (Xu et al., 2018). Amentoflavone reverses the inhibitory effect of DOX on the PI3K/AKT pathway and exerts combined hepatoprotective and nephroprotective effects through the upregulation of HO-1 expression (Fang et al., 2023). Additionally, natural products such as melatonin, artemisinin extract and omega-3 polyunsaturated fatty acids have been shown to ameliorate DOX-induced hepatotoxicity by activating the PI3K/AKT/HO-1 pathway (Espírito Santo et al., 2023; Oz and Ilhan, 2006; Saleh et al., 2022). Therefore, targeting the activation of the PI3K/AKT/HO-1 signaling pathway to mitigate oxidative stress and block downstream pathological cascades represents a promising therapeutic strategy for alleviating DOX-induced hepatotoxicity. These findings hold significant implications for the development of novel hepatoprotective agents aimed at reducing the hepatotoxic side effects of chemotherapeutic drugs.

*N*-salicyloyl tryptamine derivatives (NSTs) have been identified as multifunctional molecules with potential applications in the treatment of neurodegenerative diseases (NDDs). Among these derivatives, **A1** has demonstrated potent antioxidant, anti-inflammatory, and neuroprotective activities, as evidenced by its ability to effectively suppress the levels of ROS, malondialdehyde (MDA) and lactate dehydrogenase (LDH), while enhancing SOD activity (Su et al., 2023; Wu et al., 2024; Zeng et al., 2026; Zhao et al., 2024). Furthermore, it inhibited pro-inflammatory factor release to exert neuroprotective functions, although its antioxidant activity still remained to be improved. In medicinal chemistry, the phenolic hydroxyl group serves as a potent hydrogen donor that can directly scavenge ROS, thereby exhibiting well-characterized antioxidant activity. Given that oxidative stress is a pivotal mechanism in DOX-induced hepatotoxicity, this study sought to enhance the hepatoprotective potential of **A1** by introducing a hydroxyl group at the four-position of the salicylic acid ring, yielding the novel derivative **A50** (Scheme 1). Following the evaluation of 50 candidate NSTs, **A50** exhibited the optimal antioxidant and hepatoprotective effects in a DOX-induced hepatotoxicity cell model. *In vivo*, **A50** significantly decreased serum ALT and AST levels, and restored normal hepatic architecture in DOX-treated mice. Mechanistic investigations indicated that **A50** attenuated hepatocyte death by activating the PI3K/AKT/HO-1 signaling pathway *in vitro*, thereby suppressing oxidative stress and inflammatory responses. In conclusion, **A50** as a novel phenolic hydroxyl-containing NSTs, represented a promising candidate for the treatment of DOX-induced hepatotoxicity. These findings not only provided a new lead compound but also offered valuable insights for the development of highly effective therapeutic agents targeting DOX-induced hepatotoxicity.

**SCHEME 1:**
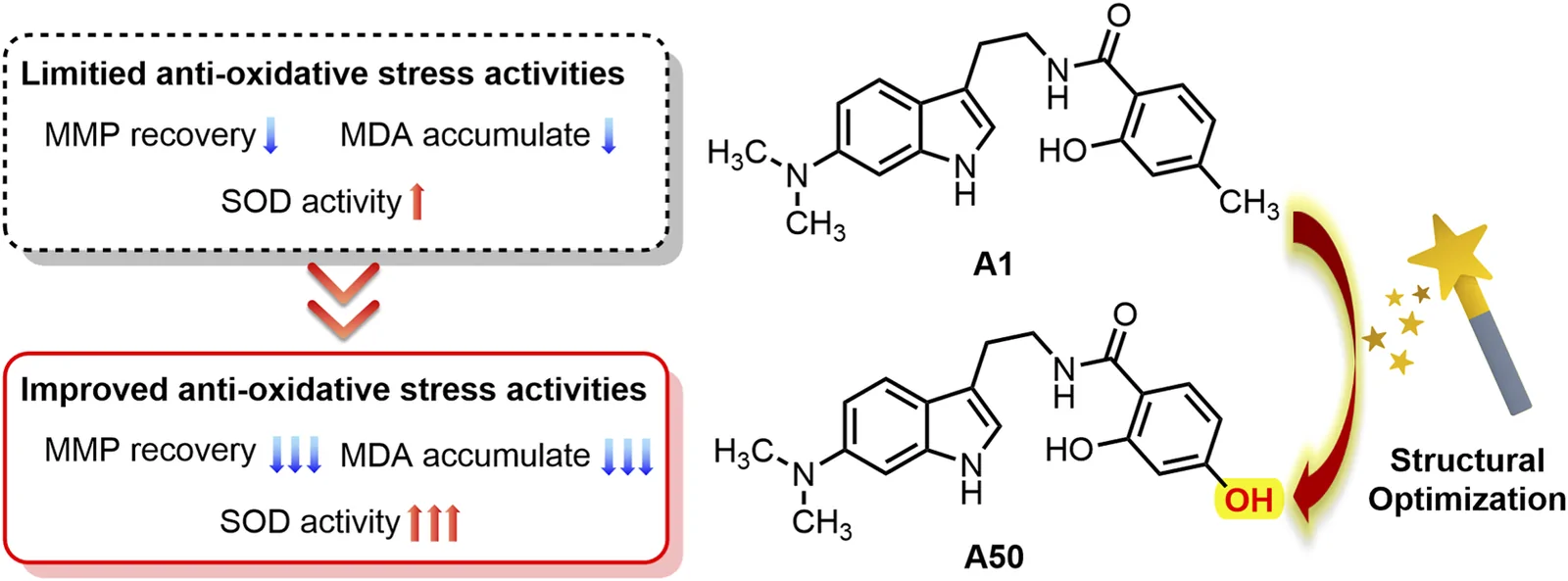
Design Strategy.

## 2 Materials and methods

### 2.1 General synthesis procedures of A50

Unless otherwise stated, all reagents and solvents were obtained from commercial suppliers and used without further purification. All reactions were conducted by using oven-dried glassware under an inert atmosphere of argon unless noted otherwise. Thin-layer chromatography (TLC) was utilized to monitor reaction progress using 25 mm × 100 mm silica gel plates and visualized with UV, KMnO_4_ or ninhydrin stains. Column chromatography was carried out on 200–300 mesh silica gel (Qingdao, China). ^1^H NMR and ^13^C NMR spectra were recorded on a Bruker AVANCE 500 spectrometer using CD_3_OD as solvents and tetramethylsilane (TMS) as an internal reference. Chemical shifts (*δ*) were reported in ppm, and coupling constants (*J*) were given in Hz. High-performance liquid chromatography (HPLC) analyses were performed on a Shimadzu SPD-20A system using an Agilent HC-C18 column (4.6 mm × 250 mm, 5 μm) with a MeOH/H_2_O (40:60) mobile phase at a flow rate of 1.0 mL/min. The purity of all tested compounds was confirmed to be more than 95%.

Step a To a solution of 6-aminoindole (1.0 mmol, 1.0 equiv) and potassium carbonate (K_2_CO_3_, 2.5 mmol, 2.5 equiv) in anhydrous tetrahydrofuran (THF, 10 mL), iodomethane (2.5 mmol, 2.5 equiv) was added at 0 °C under nitrogen atmosphere. After stirring for 10 min, the mixture was stirred overnight at room temperature. Upon completion, the reaction was quenched with ice water and extracted with ethyl acetate (EtOAc). The combined organic phase was concentrated *in vacuo*. The crude product was purified by silica gel column chromatography (petroleum ether/EtOAc = 6:1) to afford compound **S1** in a 31% yield.
Step b Compound **S1** (1.0 mmol, 1.0 equiv) was dissolved in anhydrous dichloromethane (DCM, 5 mL) to form a solution of **S1**. To a solution of *N,N-*dimethyl-2- nitrovinylamine (1.1 mmol, 1.1 equiv) in anhydrous DCM (5 mL), trifluoroacetic acid (TFA, 10 mmol, 10 equiv) was added at 0 °C under nitrogen atmosphere. After mixing uniformly, the solution of **S1** was added, and the mixture was stirred at room temperature for 2 h. The reaction was quenched with saturated aqueous sodium bicarbonate (NaHCO3) and extracted with EtOAc. The combined organic phase was concentrated *in vacuo*. The crude product was purified by silica gel column chromatography (petroleum ether/ EtOAc = 4:1) to afford compound **S2** in a 46% yield.
Step c To a solution of compound **S2** (1.0 mmol, 1.0 equiv) in anhydrous THF (10 mL), lithium aluminum hydride (LiAlH_4_, 1 mol/L in THF, 10 mL, 10 equiv) was added dropwise at −78 °C under nitrogen atmosphere. Then the mixture was stirred at room temperature for 2 h. After completion, the reaction was quenched with 10% aqueous sodium hydroxide (NaOH) solution in an ice bath, and the pH of the system was adjusted to 9. The mixture was extracted with DCM, and the combined organic phase was concentrated *in vacuo* to give crude product **S3** in approximately 80% yield.
Step d To a solution of 4-hydroxysalicylic acid (1.0 mmol, 1.0 equiv), compound **S3** (1.2 mmol, 1.2 equiv), 1-hydroxybenzotriazole (HOBt, 1.1 mmol, 1.1 equiv) and 1-ethyl-3-(3-dimethylaminopropyl)carbodiimide (EDCI, 1.1 mmol, 1.1 equiv) in anhydrous DCM (15 mL), triethylamine (0.05 mmol, 0.05 equiv) was added under nitrogen atmosphere. The mixture was stirred overnight at room temperature. Upon completion, the reaction was quenched with water and extracted with DCM. The combined organic phase was concentrated *in vacuo*. The crude product was purified by silica gel column chromatography (DCM/methanol = 20:1) to afford **A50** in a 34% yield.

#### 2.1.1 Spectral analysis of A50

##### 2.1.1.1 *N*-(2-(6-(dimethylamino)-1H-indol-3-yl)ethyl)-2,4-dihydroxybenzamide (A50)

^1^H NMR (500 MHz, CD_3_OD) *δ* 7.91 (s, 1H), 7.55 (d, *J* = 8.7 Hz, 1H), 7.50 (d, *J* = 8.7 Hz, 1H), 6.97 (s, 1H), 6.89 (d, *J* = 2.2 Hz, 1H), 6.81 (dd, *J* = 8.7, 2.1 Hz, 1H), 6.31 (dd, *J* = 8.7, 2.4 Hz, 1H), 6.29 (d, *J* = 2.4 Hz, 1H), 3.64 (t, *J* = 7.4 Hz, 3H), 3.01 (t, *J* = 7.4 Hz, 3H), 2.91 (s, 6H). ^13^C NMR (125 MHz, CD_3_OD) *δ* 169.8, 162.3, 162.1, 146.9, 137.7, 128.8, 121.6, 120.7, 118.4, 111.9, 109.7, 107.5, 106.9, 102.5, 97.1, 42.1, 40.1, 25.1. HPLC purity: 99.9%.

### 2.2 Cell culture and treatments

The Alpha mouse liver 12 (AML12) cell line was purchased from Procell (Wuhan, China). Cells were maintained in Dulbecco’s Modified Eagle Medium/Nutrient Mixture F-12 (DMEM/F12) supplemented with 10% fetal bovine serum (FBS), 5 μg/mL insulin, 5 ng/mL selenium, 40 ng/mL dexamethasone, and 5 μg/mL transferrin. Cells were incubated at 37 °C in a humidified atmosphere of 5% CO_2_. Huh7, HepG2, and BEL-7404 cells were maintained in Dulbecco’s Modified Eagle Medium (DMEM) supplemented with 10% FBS, 100 U/mL penicillin, and 100 μg/mL streptomycin. All cells were incubated at 37 °C in a humidified atmosphere of 5% CO_2_.

To establish an *in vitro* model of DOX-induced hepatotoxicity, AML12 cells were seeded in 96-well or 6-well plates. After 24 h of attachment, cells were treated with 2.5 μM DOX for 24 h. For compound screening, cells were co-treated with DOX (2.5 μM) and 50 test compounds at 10 μM, with silybin (10 µM) serving as a positive control. Based on initial screening, the potent compounds were selected for further investigation. To elucidate the protective effects, cells were co-treated with **A50** (2.5, 5, 10 µM) or silybin (10 µM) and DOX (2.5 µM) for 24 h. All compounds and silybin were dissolved in dimethyl sulfoxide (DMSO), with the final DMSO concentration not exceeding 0.1% (v/v). Control groups received vehicle alone.

To further explore the PI3K/AKT pathway, LY294002 (10 μM; MedChemExpress, China), a specific PI3K/AKT inhibitor, was used to treat AML12 cells 1 h before the DOX exposure. Untreated cells were used as control.

### 2.3 Cell counting kit-8 (CCK-8) assay

Cell viability was assessed using the CCK-8 assay (Applygen, China). Briefly, AML12 cells were seeded in 96-well plates at a density of 4 × 10^3^ cells per well, incubated overnight, and then treated with varying concentrations of the tested compounds or silybin (10 µM) and DOX (2.5 µM) for 24 h. After 24 h of drug treatment, 10 μL of CCK-8 solution was added. After 2 h of incubation, the absorbance was measured in a microplate reader (Bioteck, USA) at 450 nm.

### 2.4 Measurement of mitochondrial membrane potential (MMP)

The MMP was analyzed by using the JC-1 Mitochondrial Membrane Potential Assay Kit (Solarbio, China). AML12 cells were seeded in 6-well plates at a density of 8 × 10^4^ cells per well, incubated overnight, and then treated with **A50** (2.5, 5, 10 µM) or silybin (10 µM) and DOX (2.5 µM) for 24 h. Following staining with JC-1 at 37 °C for 20 min, cells were washed, and fluorescence intensity was measured using flow cytometry (Agilent Technologies, USA). JC-1 exhibited dual color fluorescence: it formed red emitting aggregates within mitochondria under high MMP (healthy mitochondria), whereas it existed as green-fluorescent monomers in the cytoplasm upon mitochondrial depolarization. The parameter “JC-1 monomer (percent of area)” was defined as the percentage of intact gated cell events with JC-1 monomer-derived green fluorescence relative to the total gated cell population. The detection wavelengths were set at excitation/emission of 515 nm/529 nm for green monomer fluorescence.

### 2.5 Measurement of superoxide dismutase (SOD) activity

SOD activity was determined using a Total Superoxide Dismutase Assay Kit with WST-8 (Beyotime, China) according to the manufacturer’s protocol. AML12 cells lysates or liver tissues homogenates were prepared using RIPA lysis buffer containing protease inhibitors. The assay principle is based on the inhibition of superoxide anion-generated WST-8 formazan production by SOD. Absorbance was measured at 450 nm using a microplate reader (Bioteck, USA). One unit of SOD activity was defined as the amount of enzyme that inhibits the reduction reaction by 50% under assay conditions. Results were normalized to the total protein concentration measured *via* a BCA Protein Assay Kit (Applygen, China).

### 2.6 Measurement of malondialdehyde (MDA) content

The extent of lipid peroxidation was assessed by measuring MDA levels using a Lipid Peroxidation MDA Assay Kit (Beyotime, China). Liver tissues homogenates were prepared using RIPA lysis buffer containing protease inhibitors. This assay is based on the reaction of MDA with thiobarbituric acid (TBA) to form a pink MDA-TBA adduct. Briefly, liver tissues were homogenized and mixed with the provided TBA reagent. After heating at 95 °C for 60 min and cooling, the mixture was centrifuged. The absorbance of the supernatant was measured at 532 nm using a microplate reader (Bioteck, USA). MDA concentrations were calculated using a standard curve prepared from a known MDA standard and normalized to total protein content *via* a BCA Protein Assay Kit (Applygen, China).

### 2.7 Measurement of adenosine triphosphate (ATP) content

Liver tissues or cellular ATP content were quantified using an ATP Assay Kit (Beyotime, China). Liver tissues or AML12 cells were lysed with ATP lysis buffer provided in the kit. The lysates were centrifuged at 12,000 × g for 5 min at 4 °C to remove debris. The supernatants were collected and mixed with ATP detection reagent containing luciferase, which catalyzes a light-emitting reaction in the presence of ATP. Luminescence was measured immediately using a microplate reader (Bioteck, USA). ATP concentration was determined from a standard curve generated with known ATP concentrations and normalized to total protein content *via* a BCA Protein Assay Kit (Applygen, China).

### 2.8 Animal experiments

All the animal experiments were conducted according to a protocol approved by the University Animal Care and Use Committee of University of South China (Ethics Approved No. USC2024XS015). *In vivo* experimental operations, mice were anesthetized *via* intraperitoneal injection of 50 mg/kg body weight pentobarbital sodium (Sigma-Aldrich, St. Louis, MO, USA), which was freshly dissolved in sterile 0.9% normal saline to prepare a homogeneous working solution prior to use. Adequate anesthetic depth was confirmed by the complete disappearance of pedal withdrawal and corneal reflexes, and only mice with stable anesthesia were used for subsequent experiments. Upon completion of all experimental detections, mice were humanely euthanized with an overdose of pentobarbital sodium (150 mg/kg, intraperitoneal injection). All mice were obtained from Hunan Slack Jingda Laboratory Animal Co., Ltd.

#### 2.8.1 Acute toxicity study

The acute toxicity study was performed in accordance with OECD Guidelines for the Testing of Chemicals, No. 423 (Acute Oral Toxicity–Acute Toxic Class Method). A single-dose acute toxicity test was performed in Kunming mice (6–8 weeks old, 25–30 g). Mice (n = 6, three males and three females per group) were administered a single intragastric gavage of **A50** at 500 mg/kg or an equivalent volume of normal saline. The 500 mg/kg dose of **A50** for the acute toxicity study was selected according to our previously published work (Wu et al., 2024), where we had established the preliminary safety profile of NSTs. General behavior, morbidity, and mortality were monitored closely for the first 24 h and daily for 14 days. The body weights were recorded for 14 days. After mice were sacrificed, blood was collected for complete blood count, and the heart, liver, spleen, lung, and kidney were collected for hematoxylin and eosin (H&E) staining.

#### 2.8.2 Efficacy study in DOX-induced hepatotoxicity model

Male C57BL/6 mice (6–8 weeks old, 20–25 g) were housed under standard conditions (12 h light/dark cycle, 22 °C ± 2 °C) with free access to food and water. After 1 week of acclimatization, mice were randomly divided into six groups (n = 6 per group): (1) Control (saline), (2) DOX (15 mg/kg, i.p.), (3) DOX + silybin (50 mg/kg, i.g.), and (4–6) DOX + **A50** (12.5, 25, 50 mg/kg, i.g.). Mice received daily oral administrations of saline, silybin, or **A50** for 14 consecutive days. On day 7, mice in groups 2 -6 received a single intraperitoneal injection of DOX (15 mg/kg), while the control group received an intraperitoneal injection of saline. The dose of silybin (50 mg/kg) was selected to match the highest dose of **A50** used in this study. This dose matching design was intentionally adopted to enable a direct and fair comparison of the hepatoprotective efficacy between **A50** and silybin under equivalent dosing conditions. At the endpoint, mice within each group were randomly allocated to two analytical subsets (n = 3): one subset was used for serum biochemistry, livers and hearts histopathology (H&E staining), and the other subset was used for biochemical analyses in tissues. The mice were anesthetized with pentobarbital sodium and blood was collected from the retro-orbital plexus. Serum was separated by centrifugation (3,000 × g, 10 min, 4 °C) for subsequent biochemical analysis, livers and hearts were immediately harvested. A portion of each liver and hearts was fixed in 4% paraformaldehyde for histology, another portion of liver was flash-frozen in liquid nitrogen and stored at −80 °C for biochemical assays. This prospective allocation was designed to obtain complementary functional data while minimizing the number of mice used while adhering to the 3R principles.

### 2.9 Biochemical analyses in serum and liver tissues

Serum levels of ALT, AST, LDH, creatine kinase (CK), creatine kinase MB isoenzyme (CK-MB) and myoglobin (Mb) were measured using commercially available assay kits (NJJCBIO, China), following the manufacturer’s instructions.

### 2.10 Enzyme-linked immunosorbent assay (ELISA)

The levels of pro-inflammatory cytokines (IL-6 and TNF-α) in liver homogenates were determined using commercial sandwich ELISA kits (Reedbiotech, China) according to the manufacturer’s instructions. Absorbance was read at 450 nm with a correction wavelength of 530 nm using a microplate reader (Biotek, USA).

### 2.11 Hematoxylin and eosin (H&E) staining

Formalin-fixed liver and heart tissues were processed, paraffin-embedded, and sectioned at 5 µm thickness. Sections were stained with H&E and examined under a light microscope (Nikon, Japan). Histopathological lesions were blindly scored as a summation of sinusoidal congestion, hepatocyte necrosis, and ballooning degeneration scores, each graded from 0 (no damage) to 4 (severe damage), as previously described by Suzuki et al. (Alqussair et al., 2025; Suzuki, 1999). For each sample, three random non-overlapping fields were analyzed. Microscopic assessment was conducted at ×40 magnification, with a scale bar of 50 μm.

### 2.12 Western blot analysis

AML12 cells were lysed in RIPA buffer containing protease and phosphatase inhibitors. Protein concentration was determined using a BCA assay. Equal amounts of protein (20 µg per lane) were separated by SDS-PAGE and transferred to polyvinylidene difluoride (PVDF) membranes. After blocking with 5% non-fat milk for 1 h, membranes were incubated overnight at 4 °C with primary antibodies against PI3K (Proteintech, Cat# 67071-1-Ig, China, dilution 1:1000), p-PI3K (Affinity Biosciences, Cat# AF3242, China, dilution 1:1000), AKT (Proteintech, Cat# 10176-2-AP, China, dilution 1:1000), p-AKT (Proteintech, Cat# 66444-1-Ig, China, dilution 1:1000), HO-1 (Proteintech, Cat# 10701-1-AP, China, dilution 1:1000), GAPDH (Proteintech, Cat# 10494-1-AP, China, dilution 1:1000), caspase-3 (Proteintech, Cat# 19677-1-AP, China, dilution 1:1000), cleaved caspase-3 (Proteintech, Cat# 25128-1-AP, China, dilution 1:1000), β-tubulin (Proteintech, Cat# 10094-1-AP, China, dilution 1:5000), and β-actin (Proteintech, Cat# 66009-1-Ig, China, dilution 1:5000). Followed by washing with 1 × TBST four times. Then, incubated with HRP-conjugated secondary antibodies (Applygen, Cat# V2001 or V2003, China, dilution 1:5000) for 1 h at room temperature. After washing with 1 × TBST at least four times, the immunocomplexes were detected using an ECL reagent (the Shenhua Technology Imaging System, China). All the images were quantified by densitometry using ImageJ software. The results were calculated from at least three independent experiments.

### 2.13 MD simulation analysis

The best dock conformation with the lowest energy was selected and then subjected to MD simulation analysis. MD simulations were conducted using Gromacs 2020.6. The topology for the PI3K protein was generated using the Gromacs command pdb2gmx, which utilizes the Amber99SB-ILDN force field and the SPC water model. The PI3K-**A50** complex was solvated in a dodecahedron box with a minimum distance of 1.0 nm from the solute to the box edge. Following ion addition, energy minimization, ligand restraining, and NVT/NPT equilibration, a 100 ns MD simulation was performed with 2 fs time every 10 ps. Post-simulation analysis involved periodic calibration and stability evaluation, including calculations of RMSD, RMSF, and interaction energy to assess the stability of the protein-ligand system. Structure images were created with PyMOL2, and interaction plots were generated using the QtGrace software.

### 2.14 Statistical analysis

All data were presented as mean ± standard deviation (SD). Statistical comparisons among multiple groups were performed using one-way analysis of variance (ANOVA) or two-way ANOVA followed by Tukey’s *post hoc* test. A *p*-value of less than 0.05 was considered statistically significant. All analyses were conducted using IBM SPSS Statistics 25.0.

## 3 Results

### 3.1 Chemistry

The chemical structures for compounds **A1–A50** were shown in Figure 1. These compounds were synthesized by simple condensation of corresponding salicylic acid and tryptamine derivatives. The synthesis route of compounds **A1–A49** was shown in Ref. (Bai et al., 2021; Fan et al., 2020; Zhao et al., 2024), and **A50** was illustrated in Scheme 2. The structure of the compound was characterized by ^1^H NMR and ^13^C NMR spectroscopy. Its purity was determined to be more than 95% by HPLC under the conditions of methanol/water = 40:60 (v/v) on a Shimadzu LC-20AT instrument equipped with a Supersil ODs2 column (4.6 × 250 mm, 5 µm). The detailed synthetic steps were as follows: Commercially available 6-aminoindole underwent N-methylation to afford compound **S1**, followed by coupling with N,N-dimethyl-2-nitroethyleneamine to yield compound **S2**. Reduction of compound **S2** furnished compound **S3**, which was subsequently condensed with 4-hydroxysalicylic acid to provide the target compound **A50**.

**FIGURE 1:**
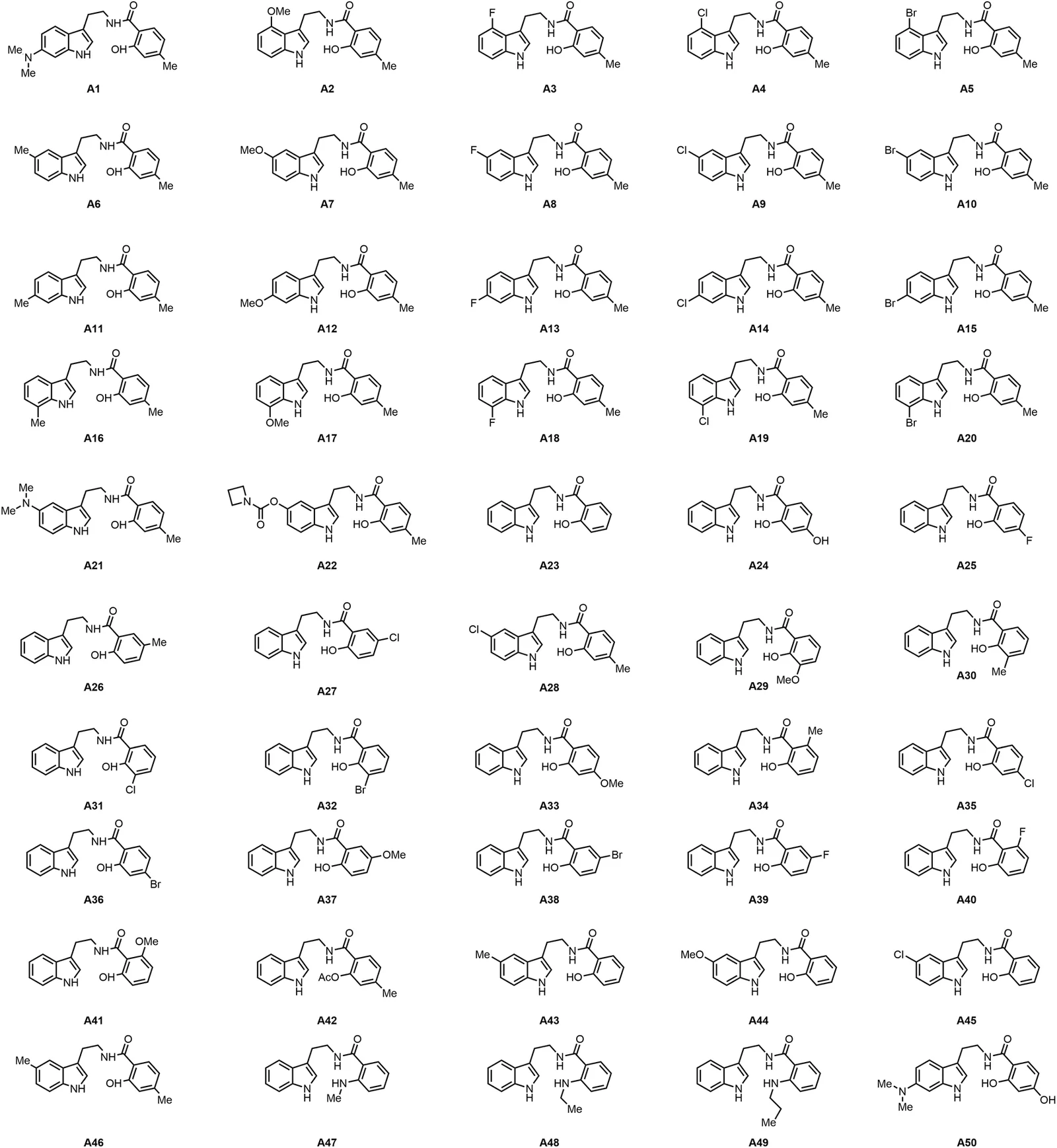
Molecular library of NSTs.

**SCHEME 2:**
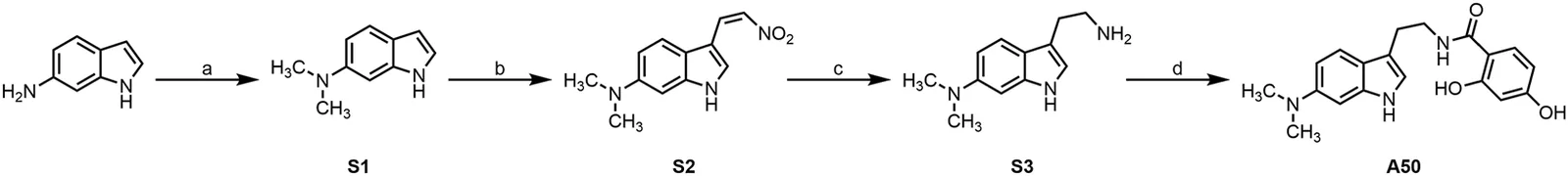
The synthetic route of target compound A50. Reagents and conditions: **(a)** Iodomethane, Potassium carbonate, THF. **(b)** *N,N*-Dimethyl-2-nitroethenamine, TFA, DCM, r.t. **(c)** LiAlH_4_, THF, −78 °C. **(d)** 2,4-Dihydroxybenzoic Acid, EDCI, HOBT, Et_3_N, DCM, r.t.

### 3.2 Effect of the NSTs on DOX-induced AML12 cells damage

To identify candidate compounds with potential protective activities against DOX-induced hepatotoxicity, we initially screened 50 compounds at a concentration of 10 μM by using CCK-8 assay in DOX-induced AML12 cell injury model. Preliminary screening results indicated that compounds **A1, A2, A3, A4, A5, A6, A7, A8, A16, A19, A43** and **A50** exhibited potential hepatoprotective activities (Figure 2A). Based on these results, we conducted a further screening using 2.5, 5 and 10 μM concentrations of the selected compounds. The results indicated that **A50** exhibited the most significant protective effect against DOX-induced hepatotoxicity compared to the positive control silybin at a concentration of 10 μM, achieving a 28.2% elevation in cell viability (Figure 2B). This structural feature may enhanced radical-scavenging capacity –the phenolic hydroxyls serve as hydrogen donors to efficiently neutralize DOX-induced overproduction ofROS, with the specific substitution pattern further stabilizing the radical intermediates. To further exclude the possibility that **A50** might interfere with the anticancer efficacy of DOX, Huh7, HepG2, and BEL-7404 cell lines were employed. The results confirmed that **A50** did not compromise the anti-proliferative effect of DOX *in vitro* (Figures 2C -E). Consequently, compound **A50** was selected for subsequent investigations of its protective activity and underlying mechanisms against DOX-induced hepatotoxicity.

**FIGURE 2:**
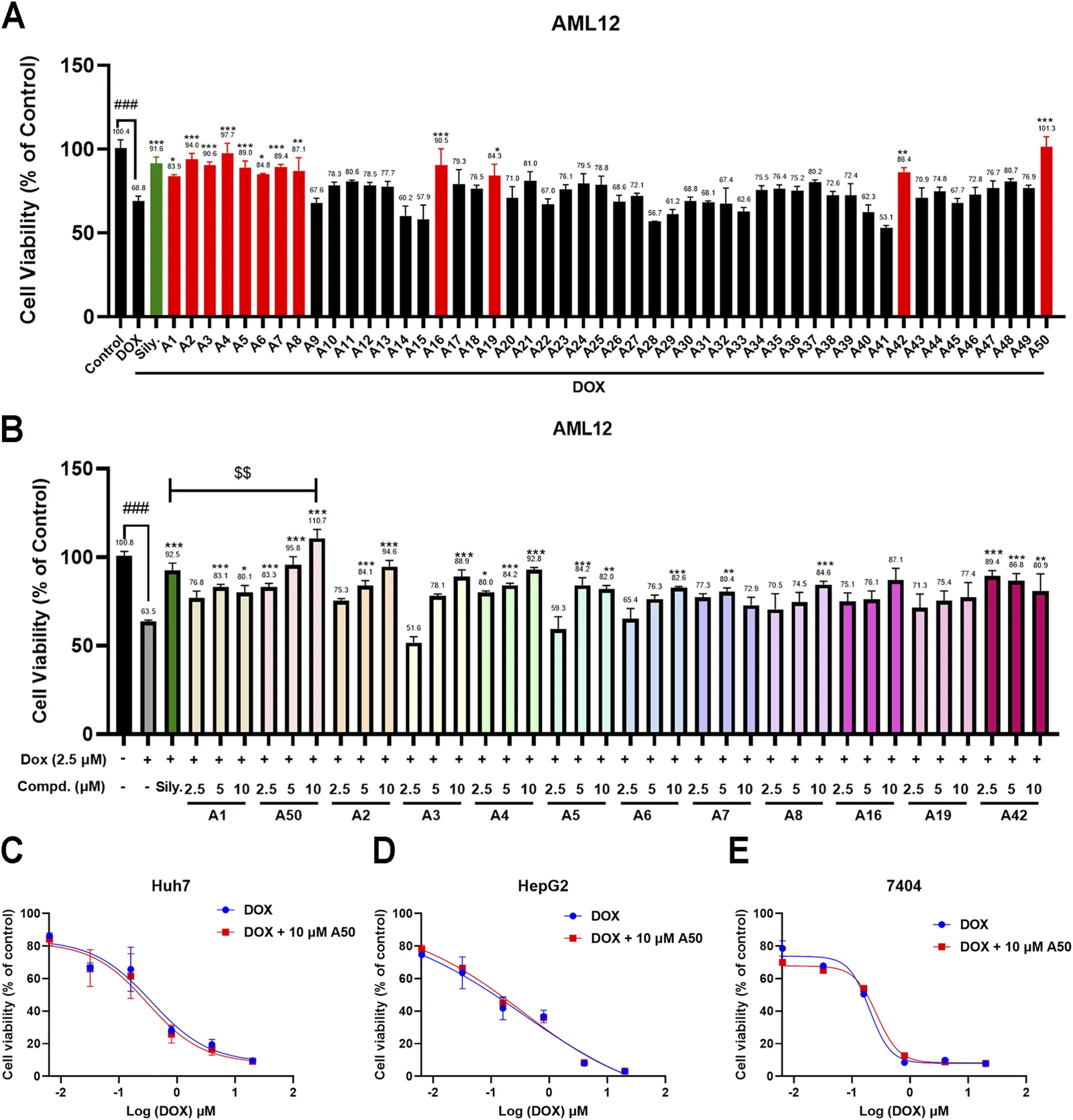
Evaluation of the protective effects of 50 NSTs against DOX-induced damage in AML12 cells. **(A)** Screening results of 50 compounds at 10 μM concentration in DOX-treated AML12 cells; **(B)** Screening results of 12 compounds (2.5, 5, 10 μM) in DOX-treated AML12 cells; Cell viability in **(C)** Huh7, **(D)** HepG2, and **(E)** BEL-7404 cells following treatment with increasing concentrations of DOX alone or in combination with 10 μM **A50**. All data were presented as the mean ± SD (n = 3). ^###^ *p* < 0.001 vs. Control group; ^*^ *p* < 0.05, ^**^ *p* < 0.01, ^***^ *p* < 0.001 vs. DOX group; ^$$^ *p* < 0.01 vs. DOX + silybin group. Determined with one-way ANOVA or two-way ANOVA followed by Tukey’s *post hoc* test.

### 3.3 A50 suppressed DOX-induced oxidative stress, mitochondrial damage and apoptosis in AML12 cells

Oxidative stress and mitochondrial dysfunction are central pathophysiological events in DOX-induced hepatotoxicity. SOD activity, MMP, and ATP levels serve as key biomarkers in this process. The structure of **A50** is shown in Figure 3A. The results demonstrated that **A50** significantly enhanced SOD activity (Figure 3B), effectively mitigating oxidative stress-induced hepatocyte damage. It also restored ATP synthesis (Figure 3C) and MMP (Figure 3D), thereby improving hepatocyte energy metabolism homeostasis. Notably, **A50** exhibited significant MMP maintenance effects at 5 and 10 μM, whereas the positive control silybin was unable to restore abnormal MMP. Furthermore, under 10 μM interventions, **A50** exhibited greater efficacy than silybin in enhancing SOD activity and promoting ATP production, with an improvement of approximately 20% -30%, indicating its promising potential for alleviating oxidative stress. Apoptosis is one of the key mechanisms underlying DOX-induced hepatotoxicity. As shown in Figure 3E, treatment with 2.5 μM DOX alone significantly increased the level of cleaved-Caspase-3 compared with the control group. However, co-treatment with increasing concentrations of **A50** (2.5, 5, and 10 μM) attenuated DOX-induced caspase-3 cleavage. These findings demonstrated that **A50** effectively inhibited DOX-triggered caspase-3 activation in AML12 cells, supporting its anti-apoptotic and cytoprotective potential.

**FIGURE 3:**
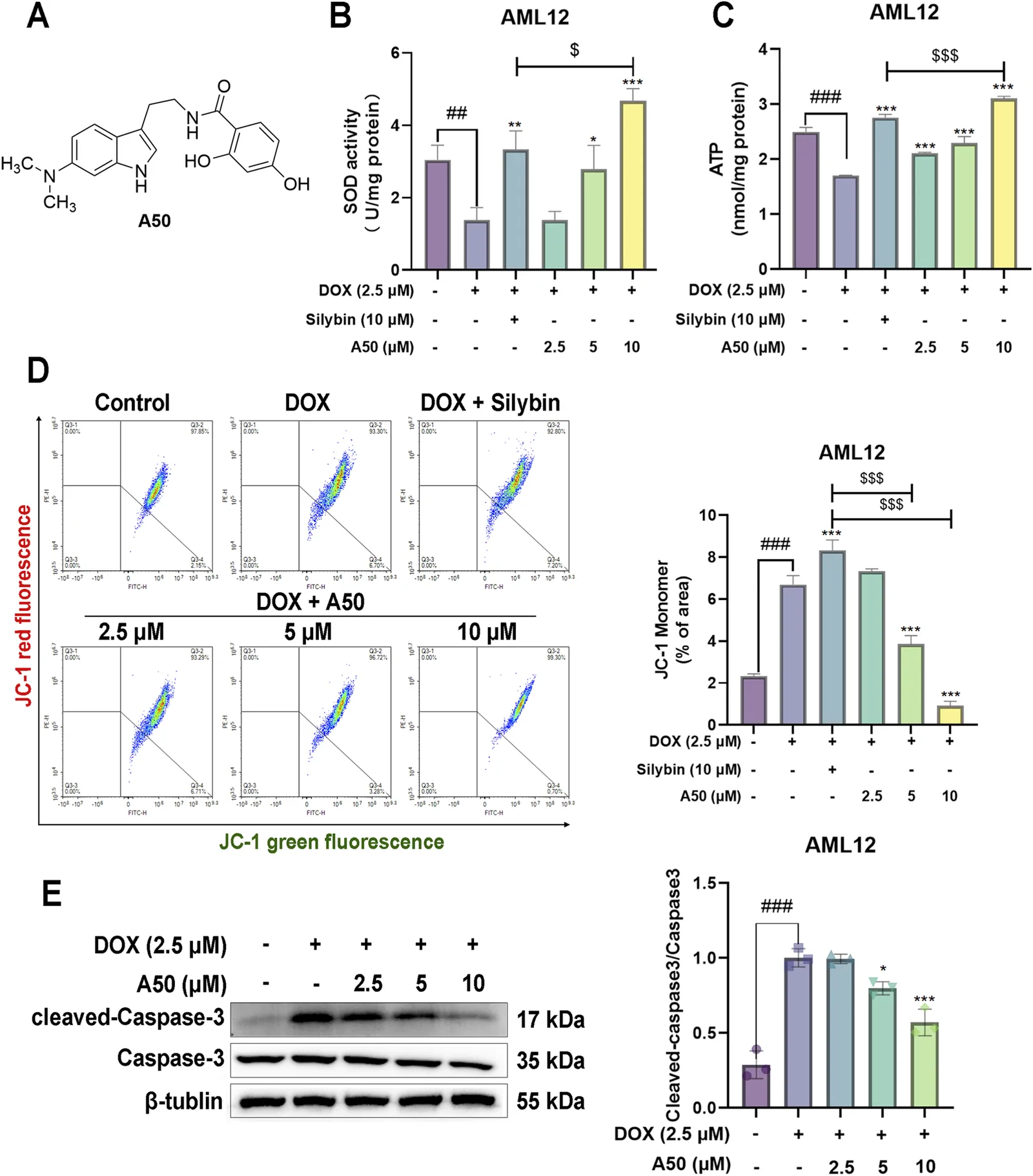
**A50** attenuated DOX-induced oxidative stress injury in AML12 cells **(A)** Chemical structure of **A50**. **(B,C)** Intracellular SOD activity and ATP content were measured using biochemical assay kits **(D)** MMP was assessed by flow cytometry. **(E)** Western blot analysis of cleaved-caspase-3 and caspase-3 protein levels in AML12 cells. All data were presented as the mean ± SD (n = 3). ^##^ *p* < 0.01, ^###^ *p* < 0.001 vs. Control group; ^*^ *p* < 0.05, ^**^ *p* < 0.01, ^***^ *p* < 0.001 vs. DOX group; ^$^ *p* < 0.05, ^$$^ *p* < 0.01, ^$$$^ *p* < 0.001 vs. DOX + silybin group. Determined with two-way ANOVA followed by Tukey’s *post hoc* test.

### 3.4 Acute toxicity study of A50

Drug safety assessment is an indispensable critical phase in new drug development. In this study, an acute toxicity test of **A50** was conducted following OECD guidelines. The experiment employed a single intragastric gavage (i.g.) at a dose of 500 mg/kg. No mortality or treatment-related abnormalities were observed within 4 h post-administration. The mice were subsequently monitored continuously for 14 days and recorded body weight data. No significant difference in average body weight was found between the control group and the **A50**-treated group (Figure 4A). On day 14, the mice were euthanized for hematological evaluations and H&E staining. As shown in Figure 4B and Table 1 H&E staining of major organs (heart, liver, spleen, lungs, and kidneys) revealed normal tissue architecture with no apparent treatment-related damage. All hematological parameters remained within physiological ranges. These findings demonstrated **A50** inherited favorable *in vivo* safety with a preliminary LD_50_ > 500 mg/kg (i.g.).

**FIGURE 4:**
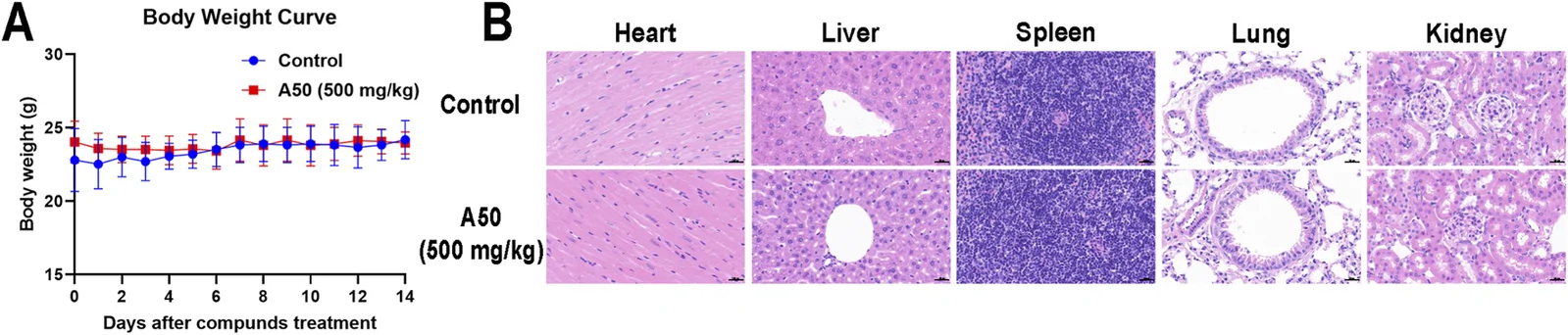
Acute toxicity experiments *in vivo* **(A)** Changes in body weight of mice within 14 days after administration of **A50**; **(B)** H&E stained tissue specimens of heart, liver, spleen, lung, and kidney of mice (magnification ×40, scale bar = 50 µm).

**TABLE 1:** Complementary hematology data of **A50**.

Parameters^a^	Control	A50 500 mg/kg	P Value summary	Parameters^a^	Control	A50 500 mg/kg	P Value summary
WBC (×10^9^/L)	0.70 ± 0.29	0.79 ± 0.23	ns	MCV (AL)	51.55 ± 0.42	51.73 ± 0.53	ns
NEU# (×10^9^/L)	1.84 ± 0.36	1.75 ± 0.63	ns	MCH (pg)	14.13 ± 0.05	14.18 ± 0.19	ns
LYM# (×10^9^/L)	0.64 ± 0.25	0.70 ± 0.24	ns	MCHC (g/L)	274.00 ± 2.94	274.00 ± 4.62	ns
MON# (×10^9^/L)	0	0	ns	RDW-CV (%)	17.23 ± 0.66	17.15 ± 0.31	ns
NE% (%)	0.60 ± 0.71	0.48 ± 0.95	ns	RDW-SD (fL)	20.58 ± 0.48	21.63 ± 1.59	ns
LY% (%)	91.18 ± 1.91	88.05 ± 8.26	ns	PLT (×10^9^/L)	765.25 ± 63.62	797.33 ± 190.90	ns
M0% (%)	0	0	ns	MPV (fL)	6.28 ± 0.17	6.50 ± 0.36	ns
RBC (×10^12^/L)	8.23 ± 1.33	11.48 ± 8.51	ns	PDW (%)	6.15 ± 0.21	6.30 ± 0.40	ns
WBC (×10^9^/L)	148.25 ± 4.5	143.50 ± 8.19	ns	MPV (×10%/L)	6.28 ± 0.17	6.50 ± 0.36	ns
NEU# (×10^9^/L)	54.10 ± 1.22	52.35 ± 2.93	ns	P-LCR (%)	2.43 ± 0.61	2.87 ± 1.08	ns

^a^ Data were expressed as mean ± SD (n = 3).

### 3.5 Effect of A50 on DOX-induced cardiotoxicity

Compound-treated groups were treated with **A50** (12.5, 25, 50 mg/kg, i.g. daily for 14 days) or silybin (50 mg/kg, i.g. daily for 14 days), control and DOX groups were gavaged with the vehicle. On the seventh day, after 30 min of corresponding treatments then injected with DOX (15 mg/kg, i.p.) or saline to establish a hepatotoxicity model (Figure 5A). Cardiotoxicity induced by DOX is also a common side effect of chemotherapy along with liver damage. To further evaluate the safety of **A50** and protective effect against DOX-induced cardiotoxicity, this study examined the impact of **A50** on myocardial histopathology and serum cardiac injury markers. Histopathological analysis revealed that DOX exposure caused disorganized arrangement and rupture of myocardial fibers alongside interstitial edema. **A50** significantly ameliorated these pathological changes, restoring structural integrity of cardiomyocytes and promoting regular fiber alignment (Figure 5B). Regarding serum biomarkers, DOX elevated the levels of CK, CK-MB and Mb, indicating myocardial cell damage. In contrast, **A50** reversed these biomarkers with greater efficacy than the positive control drug silybin, with an improvement of approximately 20%–30% (Figures 5C–E). Moreover, changes in the activity of LDH as another key non-specific marker of myocardial injury were consistent with the aforementioned myocardial enzyme results. DOX significantly increased serum LDH activity, while **A50** effectively inhibited this effect, with the 50 mg/kg group exhibiting stronger protective effects than silybin (Figure 5F). In summary, **A50** significantly alleviated DOX-induced cardiotoxicity by mitigating myocardial tissue pathological damage, suppressing the activities of myocardial injury-related enzymes and LDH.

**FIGURE 5:**
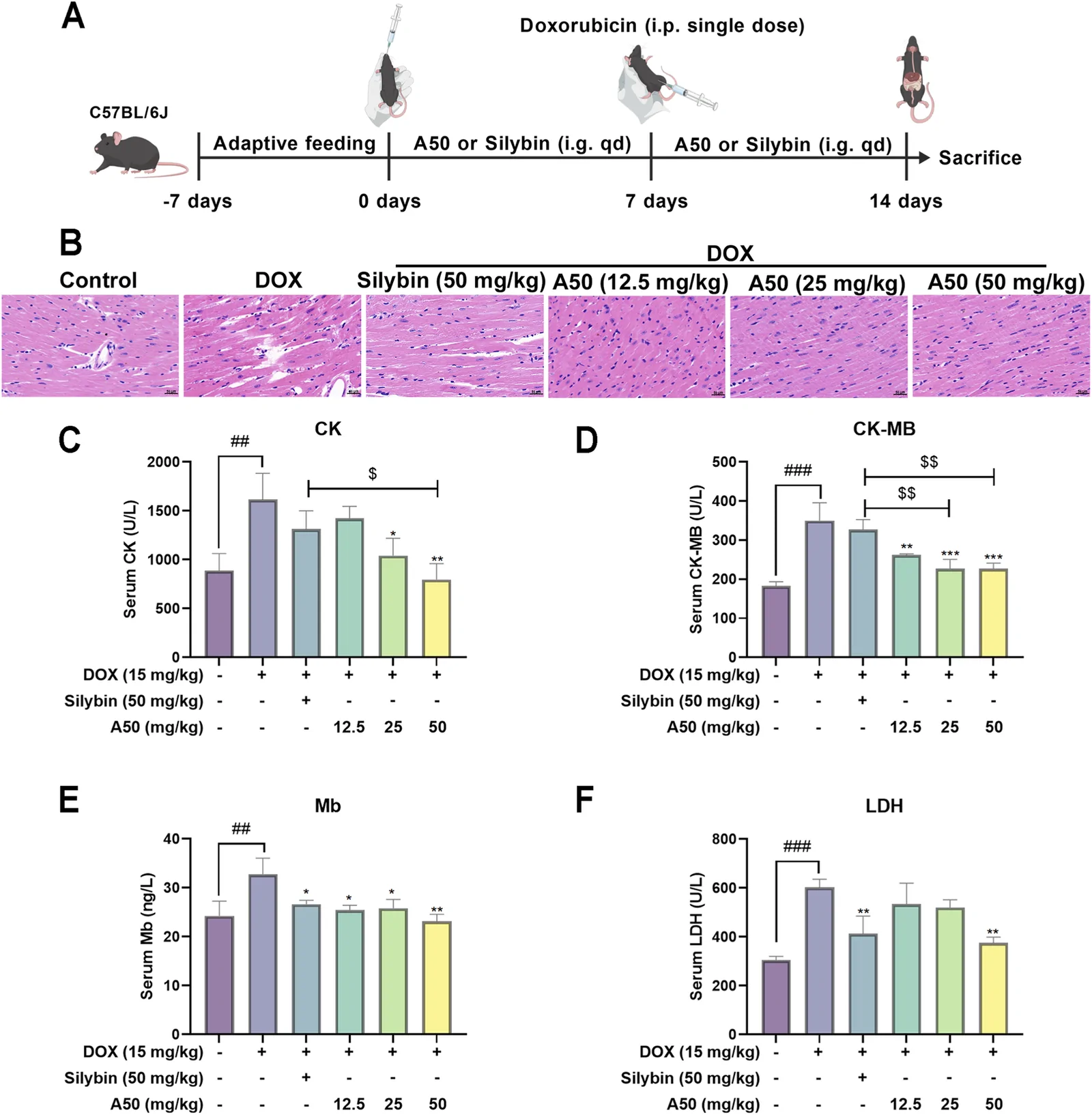
Protective effects of **A50** against DOX-induced cardiotoxicity in mice. **(A)** Experimental protocol for *in vivo* studies. i.p. (intraperitoneal injection); i.g. (intragastric gavarage); qd (once daily); **(B)** H&E-stained cardiac tissue sections (magnification ×40, scale bar = 50 µm); **(C–F)** Serum levels of CK, CK-MB, Mb and LDH in mice. All data were presented as the mean ± SD (n = 3). ^##^ *p* < 0.01, ^###^ *p* < 0.001 vs. Control group; ^*^ *p* < 0.05, ^**^ *p* < 0.01, ^***^ *p* < 0.001 vs. DOX group; ^$^ *p* < 0.05, ^$$^ *p* < 0.01 vs. DOX + silybin group. Determined with one-way ANOVA or two-way ANOVA followed by Tukey’s *post hoc* test.

### 3.6 A50 suppressed liver injury by attenuating oxidative stress and inflammation in DOX-induced mice

H&E staining histopathological analysis revealed that after DOX exposure, liver tissues exhibited significant structural disorganization, including disrupted hepatic cord arrangement, hepatocyte edema and apoptotic cell formation, indicating severe acute hepatotoxicity induced by DOX. Treatment with 12.5 mg/kg **A50** mildly improved these pathological alterations, whereas 25 mg/kg and 50 mg/kg **A50** markedly attenuated liver injury, restored hepatocyte morphology and reduced inflammatory infiltration, which were comparable to the positive control silybin (Figure 6A). Quantitative histological scoring confirmed a significant reduction in liver damage in the **A50**-treated groups compared with DOX (Figure 6B), accompanied by a recovery in body weight (Figure 6C). To further systematically evaluate the protective effect of **A50** against DOX-induced hepatotoxicity, we first measured serum levels of hepatotoxicity biomarkers. As shown in Figures 6D,E, DOX treatment significantly elevated the levels of ALT and AST, indicative of substantial hepatic damage. Following **A50** treatment, both ALT and AST levels were significantly suppressed, with a particularly notable reduction in AST. Notably, **A50** exhibited nearly 50% stronger efficacy in lowering AST levels compared with silybin. Overall, **A50** exerted better therapeutic efficacy than the positive control silybin. Further studies revealed that **A50** significantly reduced the content of MDA and enhanced the activity of the SOD in mice livers, confirming its role in inhibiting oxidative stress-induced damage (Figures 6F,G). In addition, inflammatory response is a critical pathological phase in the progression of drug-induced hepatotoxicity. DOX significantly promoted the secretion of pro-inflammatory factors IL-6 and TNF-α in mice livers. **A50** inhibited the release of both factors, with the 50 mg/kg group showing significantly greater efficacy than silybin (Figures 6H,I). In summary, **A50** effectively ameliorated DOX-induced hepatotoxicity by alleviating oxidative stress and modulating the inflammatory response, demonstrating better efficacy than silybin.

**FIGURE 6:**
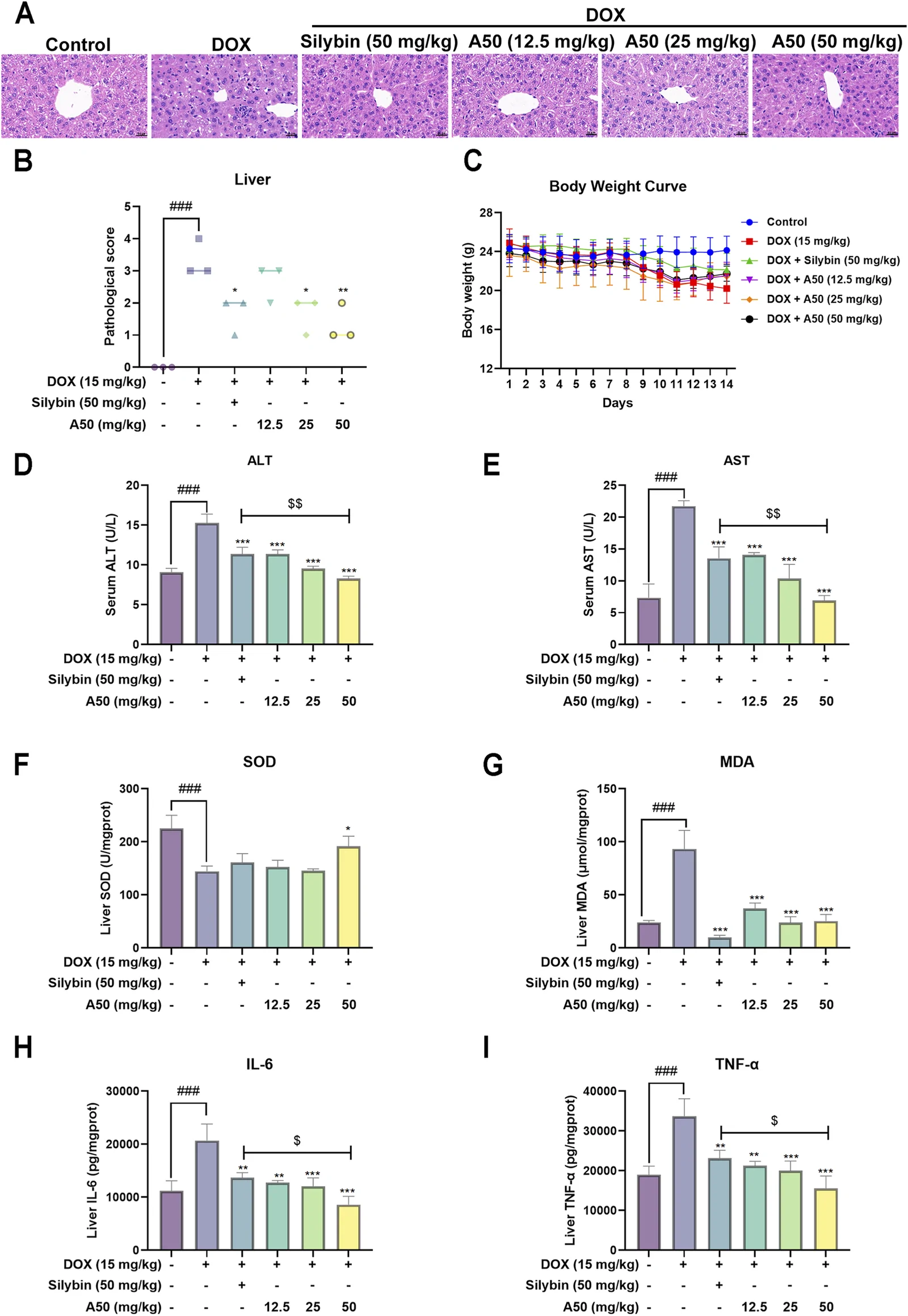
Protective effects of **A50** on DOX-induced hepatotoxicity in mice **(A)** H&E-stained liver tissue sections (magnification ×40, scale bar = 50 µm); **(B)** Semi-quantitative histological scoring of liver damage; **(C)** Changes in body weight of mice within 14 days; **(D,E)** Serum AST and ALT levels in mice; **(F)** SOD activities and **(G)** MDA levels in mice liver tissues; **(H,I)** IL-6 and TNF-α levels in mice liver tissues. All data were presented as the mean ± SD (n = 3). ^###^ *p* < 0.001 vs. Control group; ^*^ *p* < 0.05, ^**^ *p* < 0.01, ^***^ *p* < 0.001 vs. DOX group; ^$^ *p* < 0.05, ^$$^ *p* < 0.01 vs. DOX + silybin group. Determined with one-way ANOVA or two-way ANOVA followed by Tukey’s *post hoc* test.

### 3.7 Mechanism of A50 in attenuating DOX-induced hepatotoxicity *via* the PI3K/AKT/HO-1 pathway

The PI3K/AKT/HO-1 signaling pathway is one of the key mechanisms regulating DOX-induced hepatotoxicity, playing a crucial role in pathological processes such as oxidative stress, inflammatory response, and cell death. As shown in Figures 7A–D, after DOX treatment, the levels of PI3K and AKT proteins did not change significantly, while the level of p-PI3K was significantly reduced. This subsequently inhibited the downstream p-AKT level, ultimately leading to downregulation of HO-1 expression. Following **A50** administration, p-PI3K, p-AKT, and HO-1 expression were significantly upregulated, implying that **A50** mitigated DOX-induced hepatic oxidative stress damage by activating the PI3K/AKT/HO-1 pathway. Next, the PI3K inhibitor, LY294002, was used to verify the involvement of PI3K signaling in the hepatoprotective mechanism of **A50**. After the addition of LY294002, the activation of p-PI3K, p-AKT, and HO-1 by **A50** treatment was reversed (Figures 7E–H). These findings suggested that the protective effects of **A50** against DOX-induced hepatotoxicity *via* PI3K/AKT/HO-1 signaling pathway *in vitro*.

**FIGURE 7:**
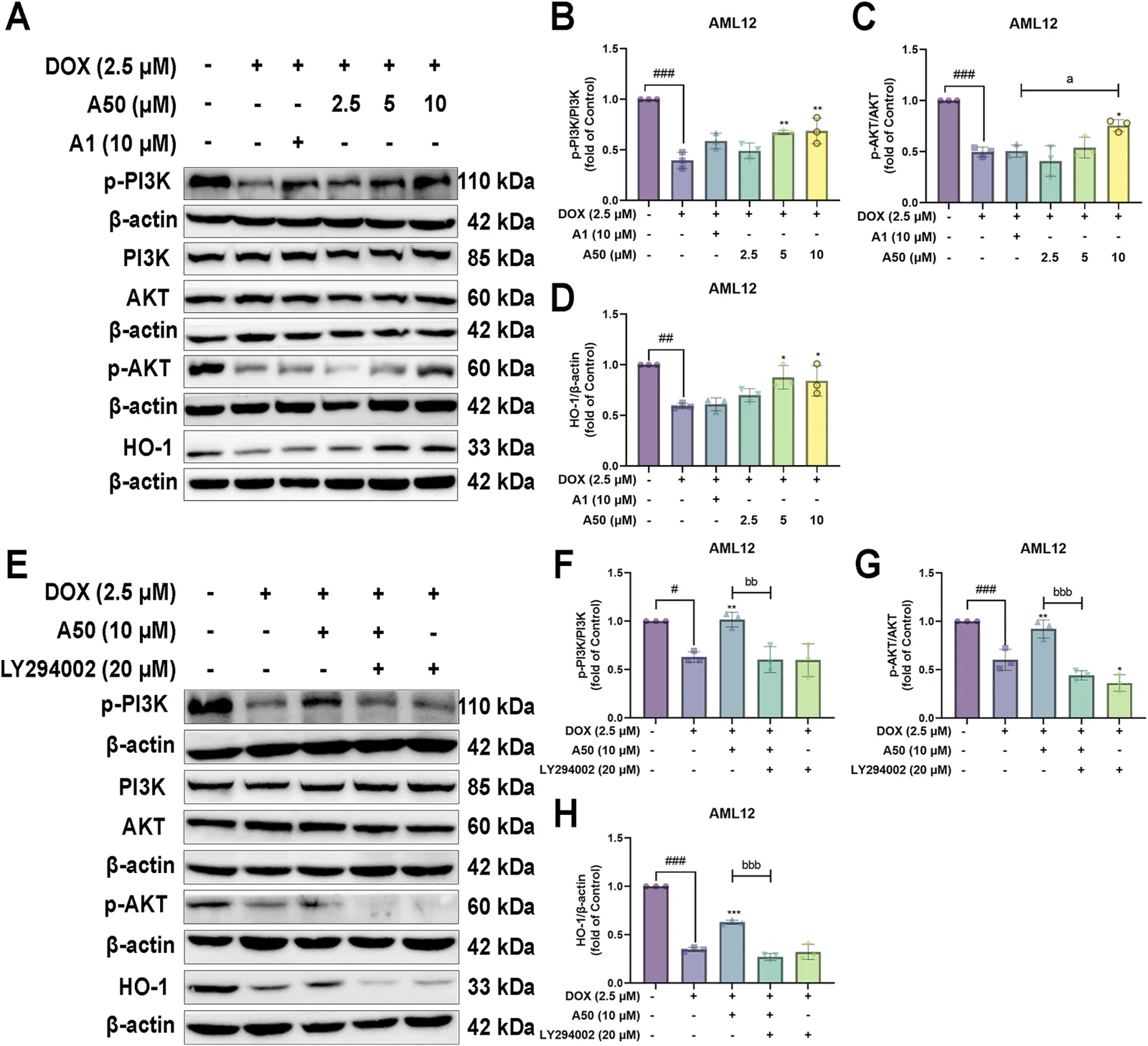
**A50** exerted hepatoprotective effects by regulating the PI3K/AKT/HO-1 pathway **(A)** Western blot analysis of p-PI3K, PI3K, AKT, p-AKT, and HO-1 expression; **(B)** Histogram showing the relative ratio of p-PI3K/PI3K; **(C)** Histogram showing relative p-AKT/AKT ratio; **(D)** Histogram showing HO-1 expression levels; **(E)** Western blot analysis of p-PI3K, PI3K, AKT, p-AKT, and HO-1 after addition of PI3K inhibitor LY294002 (n = 3); **(F)** Histogram showing the relative ratio of p-PI3K/PI3K; **(G)** Histogram showing the relative ratio of p-AKT/AKT; **(H)** Histogram showing the expression level of HO-1. All data were presented as the mean ± SD (n = 3). #p < 0.05, ##p < 0.01, ###p < 0.001 vs. Control group; *p < 0.05, **p < 0.01 vs. DOX group; ap < 0.05 vs. DOX + A1 group, bbp < 0.01, bbbp < 0.001 vs. DOX + **A50** group. Determined with one-way ANOVA or two-way ANOVA followed by Tukey's post hoc test.

### 3.8 Binding mode of A50 with PI3K: molecular docking and dynamics simulations

To systematically investigate the binding mode and structural stability of **A50** within the PI3K binding pocket, molecular docking was first performed, followed by molecular dynamics (MD) simulations to validate the stability of the predicted complexes.

In the **A50**-PI3K complex, the ligand engaged in multiple interactions within the binding pocket. Specifically, **A50** exhibited two π -hydrogen interactions: one between its indole ring and Ile932, and the other between its indole NH group and Tyr836. Additionally, the hydroxyl group of **A50** formed a hydrogen bond with Gln859, and the amide NH group of **A50** formed another hydrogen bond with Val851 (Figures 8A,B). The phenolic hydroxyl moiety of **A50** remodulated molecular polarity and electronic distribution, which promoted a favorable binding conformation and facilitated hydrogen-bonding interactions with key residues lining the PI3K pocket.

**FIGURE 8:**
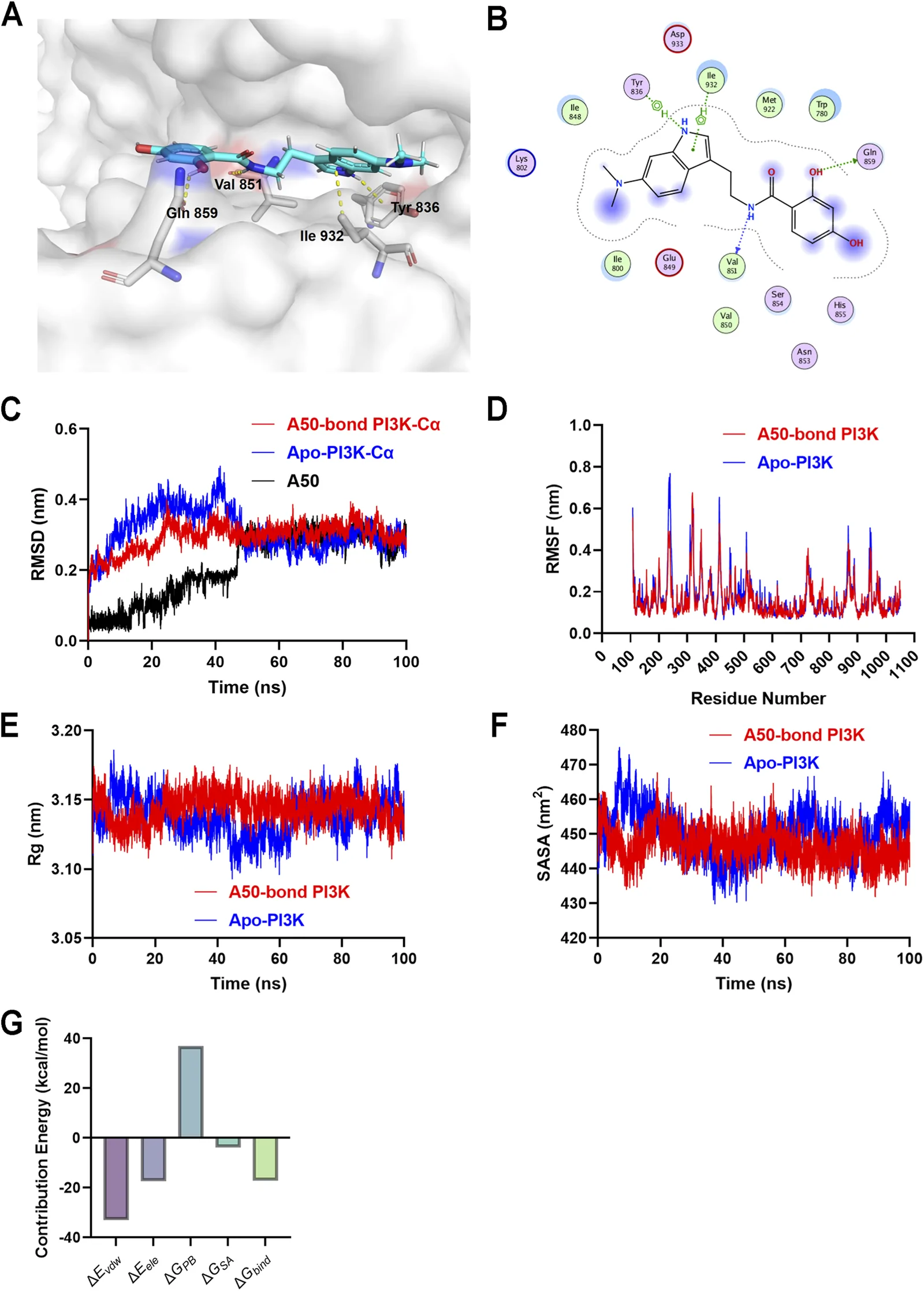
Molecular docking and molecular dynamics analysis **(A)** Interactions of **A50** with the residues in the PI3K pocket. (PDB ID: 6PYS); **(B)** Predicted two-dimensional binding mode of **A50** towards PI3K; **(C)** The RMSD of PI3K Cα atoms (apo and **A50**-bound) during the 100 ns MD simulations; **(D)** RMSF comparison of PI3K residues in the presence and absence of **A50**; **(E)** Rg analysis of PI3K with and without **A50** during the 100 ns MD simulation; **(F)** SASA profiles of the **A50**-PI3K complexes during the 100 ns MD simulation; **(G)** Contribution energy component analysis of the **A50**-PI3K complexes over the 100 ns MD simulation.

To validate these binding features, 100 ns MD simulations were performed for the **A50**-PI3K complex and apo-PI3K systems.

The root-mean-square deviation (RMSD) values of both the apo-PI3K and the **A50**-PI3K complex remained relatively stable throughout the simulations (Figure 8C). For the apo-PI3K and the **A50**-PI3K complex, the RMSD values stabilized at approximately 0.3 -0.4 nm after an initial ∼40 ns equilibration phase, while the RMSD of the ligand **A50** remained consistently low (<0.3 nm) throughout the simulation, indicating **A50**-PI3K complex achieved a stable binding configuration without significant positional drift. Root-mean-square fluctuation (RMSF) analysis revealed that both systems exhibited similar profiles, with high fluctuations localized to known flexible loop regions (Figure 8D). Notably, residues lining the **A50**-binding pocket (including Tyr836, Gln859, and Ile932) showed significantly reduced RMSF values in the **A50**-bound complex compared to apo-PI3K, indicating that ligand binding markedly restricted local flexibility at the active site and stabilized the protein's active conformation.

Radius of gyration (Rg) analysis further demonstrated the structural compactness of the systems: both the **A50**-PI3K complex and apo-PI3K maintained stable Rg values (∼3.13 - 3.17 nm) throughout the simulation, with no significant drift or unfolding events observed (Figure 8E). These results confirmed that **A50** binding did not induce large-scale conformational rearrangements in the global fold of PI3K. Solvent-accessible surface area (SASA) analysis further supported these findings, with the **A50**-PI3K complex exhibiting a slightly lower and more stable average SASA profile (430 -470 nm2) compared to apo-PI3K, consistent with stable encapsulation of the ligand within the binding pocket and reduced solvent exposure of the protein (Figure 8F).

Finally, MM-PBSA analysis was performed to decompose the binding free energy of the **A50**-PI3K complex (Figure 8G). The van der Waals interaction energy (ΔE_*vdW*_) contributed the most favorable component (∼-30 kcal/mol), followed by electrostatic interactions (ΔE_*ele*_, ∼-10 kcal/mol). Although the polar solvation energy (ΔG_*PB*_) was unfavorable (∼+35 kcal/mol), the non-polar solvation energy (ΔG_*SA*_) contributed slightly favorably (∼-5 kcal/mol). The calculated total binding free energy (ΔG_*bind*_) of approximately −15 kcal/mol confirmed that **A50** formed a thermodynamically stable complex with PI3K, driven primarily by van der Waals and electrostatic interactions.

Taken together, the docking and MD simulation results demonstrated that **A50** engaged in a well-balanced network of hydrogen-bonding and hydrophobic interactions within the PI3K binding pocket, conferring enhanced conformational stability and favorable binding energetics. These findings provided a structural basis for the potent binding of **A50** to PI3K.

## 4 Discussion

Chemotherapy remains a cornerstone of cancer treatment. DOX, as a highly effective and broad-spectrum anthracycline drug, is widely used in treating hematologic malignancies and various solid tumors. However, its severe multi-organ toxicity significantly limits its application. The liver, as the primary organ for detoxification and drug metabolism, is particularly susceptible to damage. Current clinical strategies for mitigating DOX-induced liver toxicity primarily rely on symptomatic relief through antioxidants, iron chelators and anti-inflammatory agents.

Nevertheless, there remains a lack of highly effective and specific hepatoprotective drugs capable of specifically blocking the complex interaction network involving DOX-induced oxidative stress and its subsequent effects on mitochondrial damage, inflammation and apoptosis. Therefore, the development of novel hepatoprotective agents represents a critical and unmet clinical need. Based on our previous research that NSTs exhibited potent antioxidant, anti-inflammatory, and neuroprotective activities in NDDs. This study extended its application to the treatment of DOX-induced hepatotoxicity and modified the scaffold by introducing potent hydrogen-donating phenolic hydroxyl groups. Through subsequent screening, **A50** was identified as a candidate with promising hepatoprotective activity. And a systematic evaluation was conducted of **A50** to characterize its hepatoprotective effects and elucidate the underlying molecular mechanisms.

To comprehensively evaluate the hepatoprotective effects of **A50** against DOX-induced hepatotoxicity, we performed systematic analysis of serum biochemical markers and liver histopathology. Results showed that mice treated with DOX exhibited significantly increased serum ALT and AST levels, indicating severe hepatic dysfunction. Histopathological examination revealed marked inflammatory infiltration, disruption of hepatic cord architecture, and extensive apoptotic cell death—features consistent with classic drug-induced hepatotoxicity (Al-Qahtani et al., 2022; Kalantary-Charvadeh et al., 2023; Qaed et al., 2026). Notably, administration of **A50** at different doses (12.5, 25, and 50 mg/kg) led to a significant reduction in serum ALT and AST levels. Furthermore, liver tissues exhibited improved structural organization and marked decrease in cell death. Collectively, these results demonstrated that **A50** effectively attenuated DOX-induced hepatotoxicity.

Oxidative stress is widely recognized as a central mechanism underlying DOX-induced hepatotoxicity. During hepatic metabolism of DOX, excessive accumulation of superoxide anions, hydroxyl radicals and hydrogen peroxide overwhelms the clearance capacity of endogenous antioxidant defenses, resulting in oxidative damage to lipids, proteins and DNA, ultimately culminating in cell death. Following administration of **A50**, MDA levels were significantly reduced, SOD activity was enhanced, and MMP was restored, accompanied by improved ATP production. These findings indicated that the hepatoprotective effects of **A50** were closely associated with its potent antioxidant activity, thereby validating the scaffold modification strategy of introducing phenolic hydroxyl groups to enhance anti-oxidative stress activity.

To elucidate the molecular mechanism underlying the antioxidant and hepatoprotective effects of **A50**, we investigated the PI3K/AKT/HO-1 signaling axis, a critical pathway involved in redox homeostasis regulation and cell survival. Our findings revealed that DOX suppressed the expression of p-PI3K, p-AKT, and their downstream HO-1 protein, consistent with previous reports that DOX exacerbated oxidative stress *via* inhibiting this pathway (Alwaili et al., 2025; Liu et al., 2021; Saleh et al., 2022). In contrast, administration of **A50** significantly upregulated the levels of p-PI3K, p-AKT, and HO-1 *in vitro*. This effect was blocked by the PI3K-specific inhibitor LY294002, thereby attenuating its hepatoprotective effect. This indicated that **A50** may alleviated DOX-induced hepatotoxicity by reducing oxidative stress and inflammatory responses *via* activating the PI3K/AKT/HO-1 pathway.

To our surprise, **A50** not only showed favorable safety *in vivo* (LD_50_ > 500 mg/kg) but also exhibited significant protective potential against DOX-induced myocardial injury. Specifically, **A50** markedly reduced serum levels of cardiac injury biomarkers (CK, CK-MB, Mb and LDH), improved myocardial histopathological architecture, and decreased cardiomyocyte apoptosis. Given that the hepatotoxicity and cardiotoxicity of DOX share highly overlapping core mechanisms, such as oxidative stress, inflammation and cell death. The multi-organ protective effects of **A50** suggested that it may acts on a common upstream pathway—the activation of the PI3K/AKT/HO-1 signaling pathway. This pathway is not only critical in the liver but may also exert protective effects in cardiomyocytes, providing a reasonable explanation for the broad-spectrum organ-protective properties of **A50**. Nevertheless, the molecular mechanisms underlying its cardioprotective effect, as well as the relationship and distinctions between its hepatoprotective and cardioprotective activities, remained to be elucidated in future studies.

One limitation of the present study is that the preservation of DOX anti-tumour activity by **A50** was only verified in hepatoma cell lines. Further validation using hepatocellular carcinoma xenograft models is needed to confirm whether **A50** maintains the anti-tumour potency of DOX *in vivo*.

## 5 Conclusion

This study systematically investigated the protective effects of **A50** against DOX-induced hepatotoxicity and its molecular mechanisms. Results demonstrated that **A50** attenuated oxidative stress damage, maintained mitochondrial homeostasis, suppressed subsequent inflammatory responses and hepatocyte death, without compromising its anti-hepatocellular carcinoma activity. Consequently, it effectively ameliorated DOX-induced liver dysfunction and histopathological alterations both *in vivo* and *in vitro*. Molecular mechanism studies revealed that **A50** exerted its hepatoprotective effects by activating the PI3K/AKT/HO-1 signaling pathway. Specifically, it upregulated the levels of p-PI3K, p-AKT and the downstream HO-1 level. This effect was reversed by the PI3K-specific inhibitor LY294002 *in vitro*. Molecular docking and molecular dynamics simulations further demonstrated the strong and stable interaction between **A50** and the PI3K active site. In summary, **A50** served as a novel hepatoprotective candidate by regulating the PI3K/AKT/HO-1 signaling pathway to suppress oxidative stress and inflammatory cascades, offering a promising therapeutic prospect for DOX-induced hepatotoxicity.

## Data Availability

The original contributions presented in the study are included in the article/Supplementary Material, further inquiries can be directed to the corresponding authors.
