# Supplementary material for: N-salicyloyl tryptamine derivatives as potential therapeutic agents for doxorubicin-induced hepatotoxicity via PI3K/AKT/HO-1 signaling pathway
Source: Front Pharmacol. 2026 Sep 24;17:1929788. doi: 10.3389/fphar.2026.1929788 (PMC13646450; doi:10.3389/fphar.2026.1929788)

Supplementary material

N-salicyloyl tryptamine derivatives as potential therapeutic agents for doxorubicin-induced hepatotoxicity via PI3K/AKT/HO-1 signaling pathway

Yuting Zhao[a,1], Jing Zeng[a,1], Guanglong Pang[d,e,1], Ying Wu[a], Shuzhi Wang[a], Zhengwen Lei[c], Xiangyong Hao[e], Yaxin Chen[a], Chengxuan Hu[a], Xianlin Zeng[a], Xinyan Huang[b*], Xinlin Zhong[b*], Zhen Wang[a,f,g,h*], Xue Peng[a*]

[a] School of Basic Medicine, School of Pharmaceutical Science, Hengyang Medical School, University of South China, Hengyang, Hunan, 421001, China

[b] Hengyang Traditional Chinese Medicine Hospital, Hengyang, Hunan, 421001, China

[c] Department of Thoracic and Cardiovascular Surgery, The First Affiliated Hospital, Hengyang Medical School, University of South China, Hengyang, Hunan, 421001, China

[d] The First Clinical Medical College of Gansu University of Chinese Medicine, Gansu Provincial Hospital, Lanzhou, 730000, China

[e] Department of General Surgery, Gansu Provincial Hospital, Lanzhou, 730000, China

[f] Department of Oncology, The Affiliated Nanhua Hospital, Hengyang Medical School, University of South China, Hengyang, Hunan, 421001, China

[g] Qinghai Provincial Key Laboratory of Tibetan Medicine Research, Northwest Institute of Plateau Biology, Chinese Academy of Sciences, Xining, Qinghai, 810008, China

[h] National Health Commission Key Laboratory of Birth Defect Research and Prevention Hunan Provincial Maternal and Child Health Care Hospital, Changsha, Hunan, 410008, China

[1]These authors contributed equally to the work.

[*]Corresponding author： huangxy123123@aliyun.com (X. Huang), 1259106525@163.com (X. Zhong), zhenw@usc.edu.cn (Z. Wang), pengxue@usc.edu.cn (X. Peng).

Table of Contents

1. ^{1}H NMR and ^{13}C NMR for compound **A50**..........**S2**
2. HPLC results for compound **A50**..........**S3**
3. Western blot of Caspase-3, cleaved-Caspase-3, β-Tublin..........**S4**
4. Western blot of HO-1, PI3K, p-PI3K, AKT and p-AKT..........**S7**

^{1}H NMR and ^{13}C NMR for compound A50

*N-(2-(6-(dimethylamino)-1H-indol-3-yl)ethyl)-2,4-dihydroxybenzamide (**A50**)*

^{1}H NMR Spectrum of A50 (500 MHz, CD_3OD)

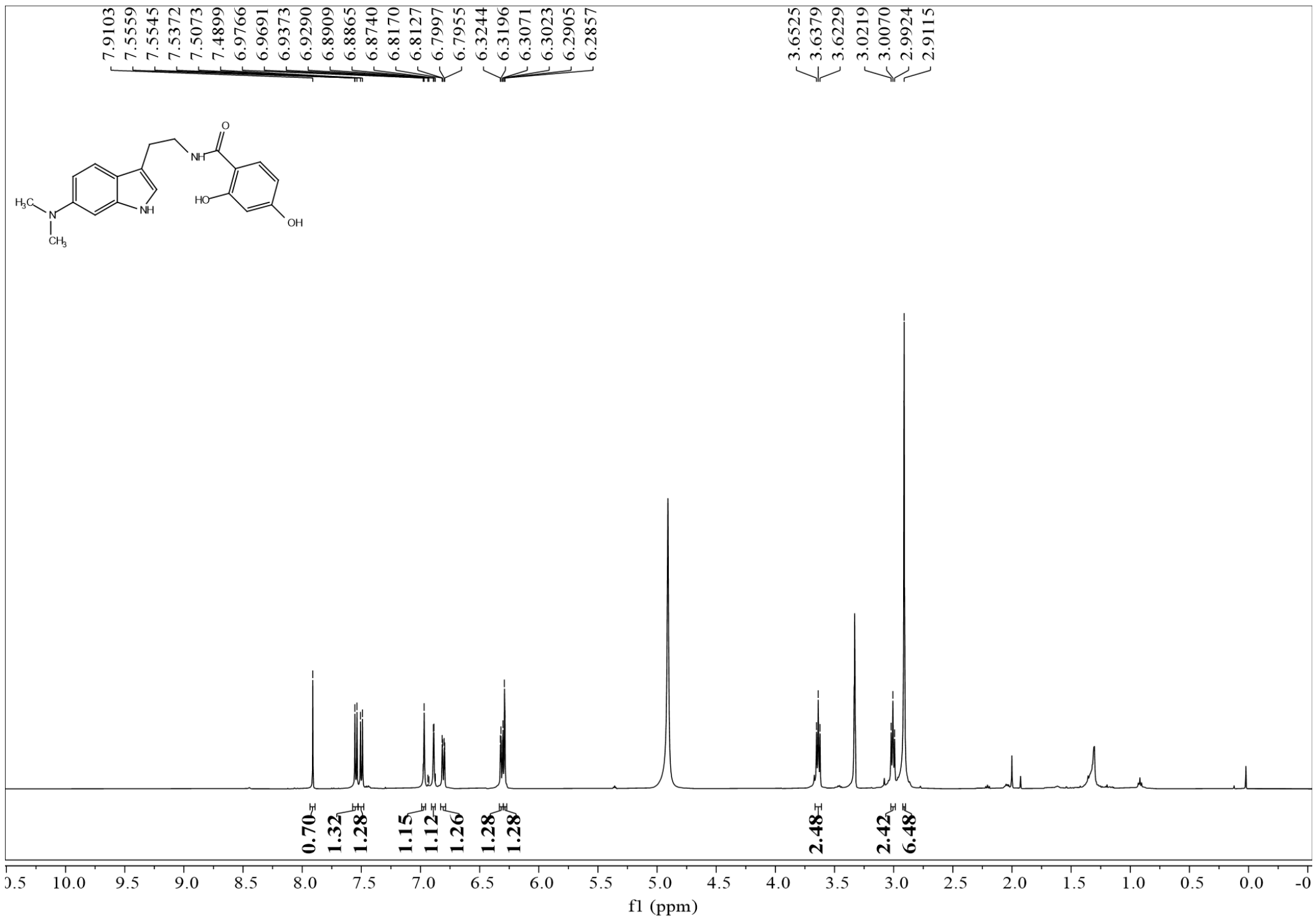

^{13}C NMR Spectrum of A50 (500 MHz, CD_3OD)

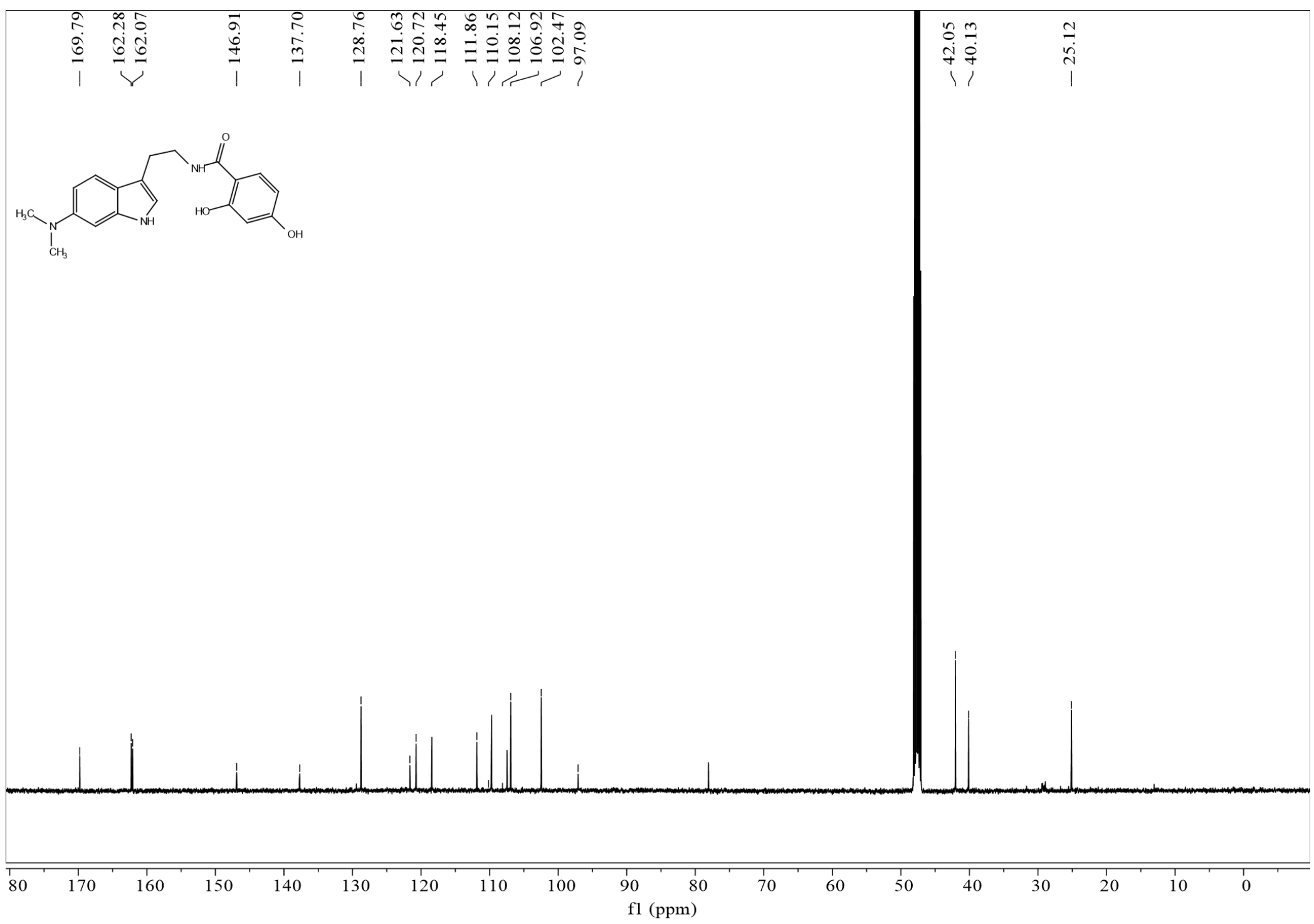

HPLC results for compound A50

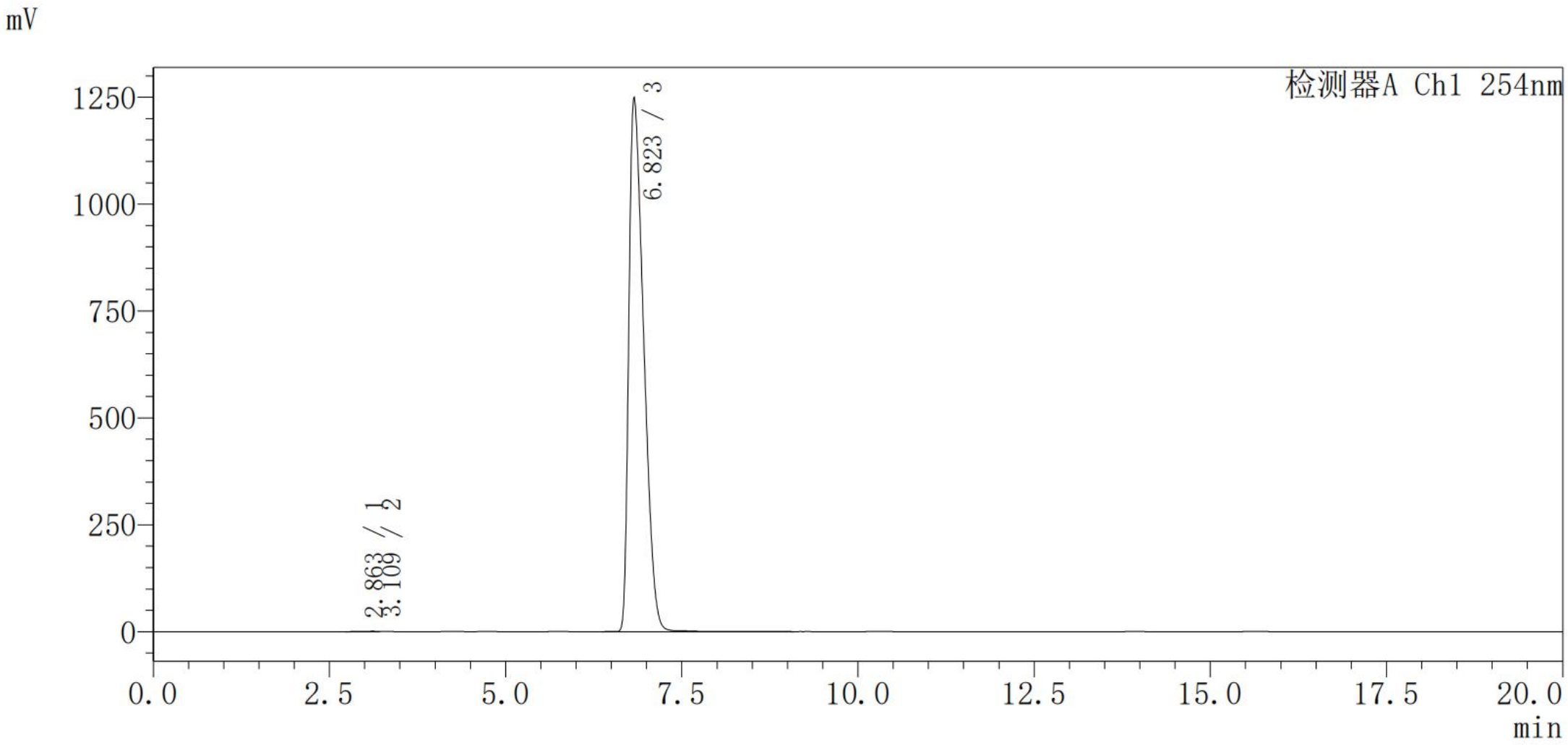

Peak #	RetTime [min]	Area [mAU*s]	Height [mAU]	Area %
1	2.863	5001	702	0.027
2	3.109	8638	1397	0.047
3	6.823	18400981	1249794	99.926

Western blot of Caspase-3, cleaved-Caspase-3, β-Tublin (Figure 3 E. Repeat 1-3)

(1) Caspase-3

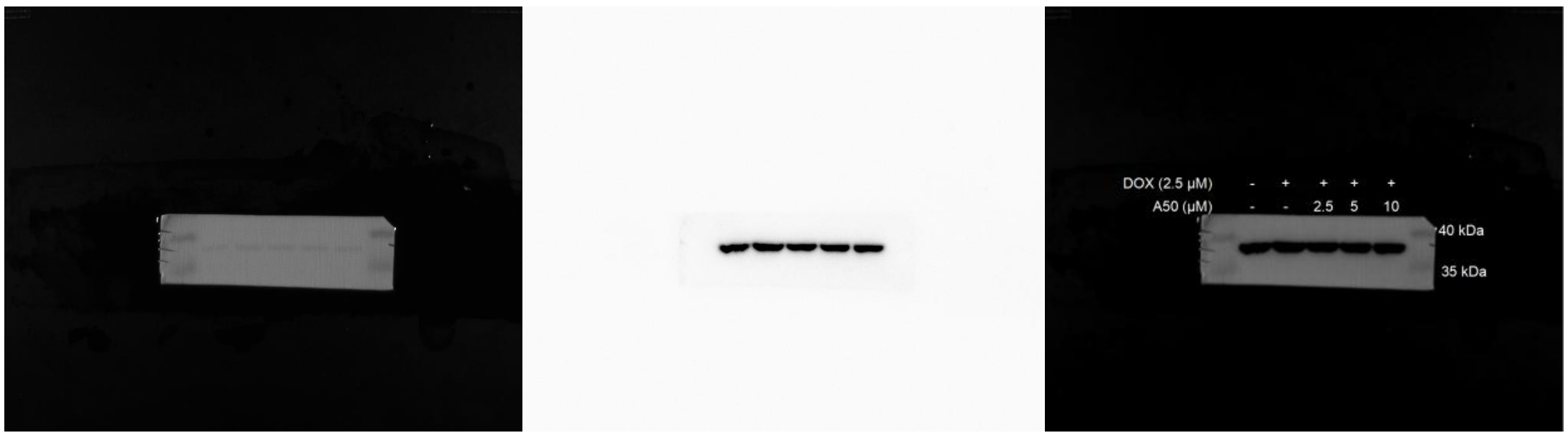

Cleaved-Caspase-3

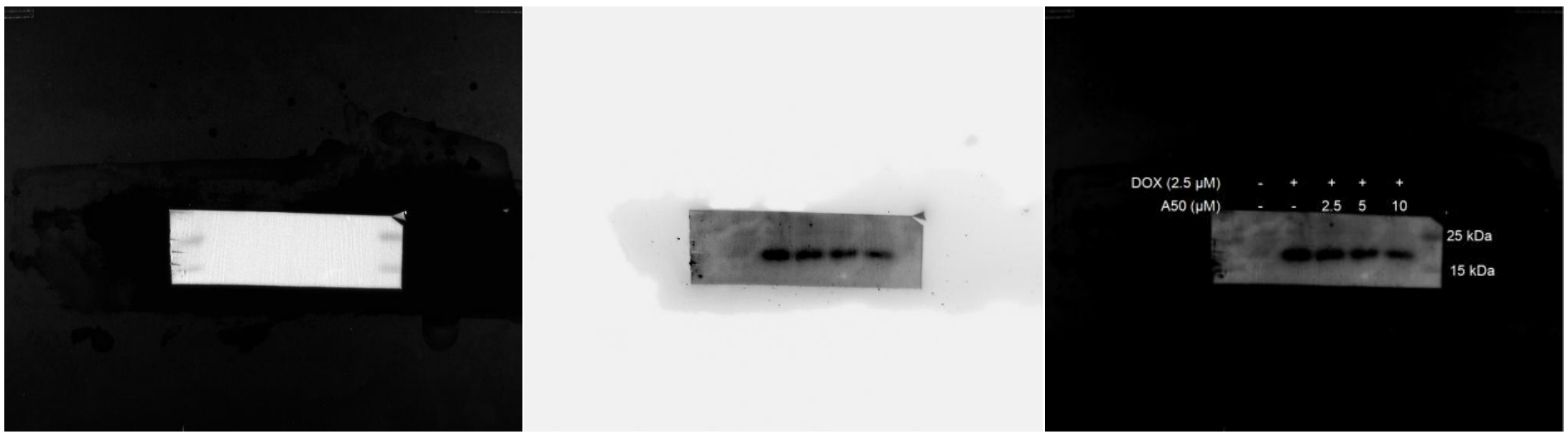

β-Tublin

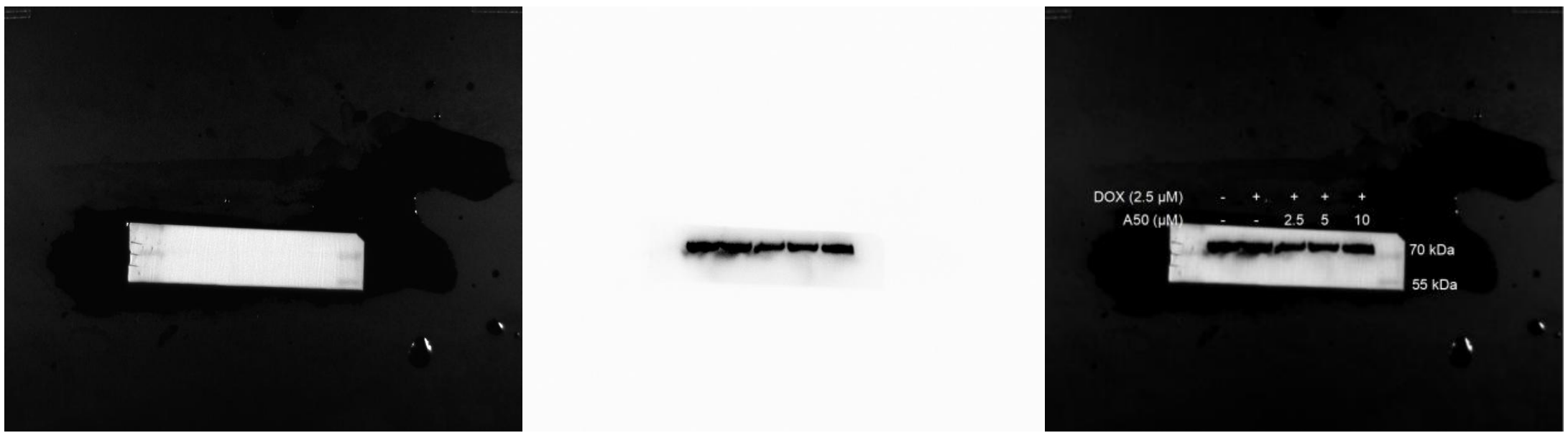

Full-size

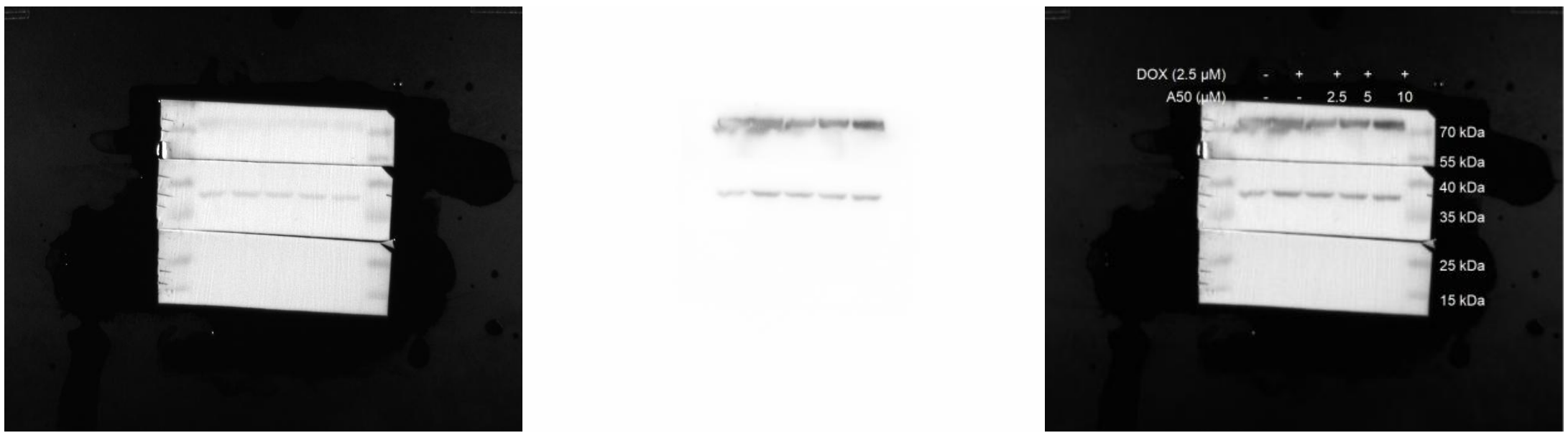

(2) Caspase-3

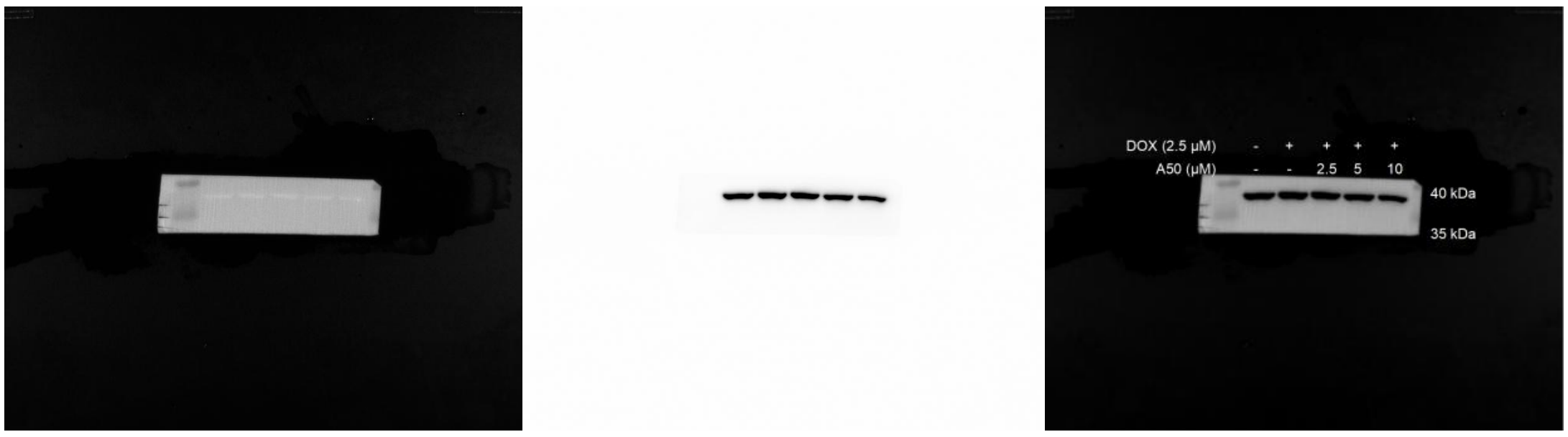

Cleaved-Caspase-3

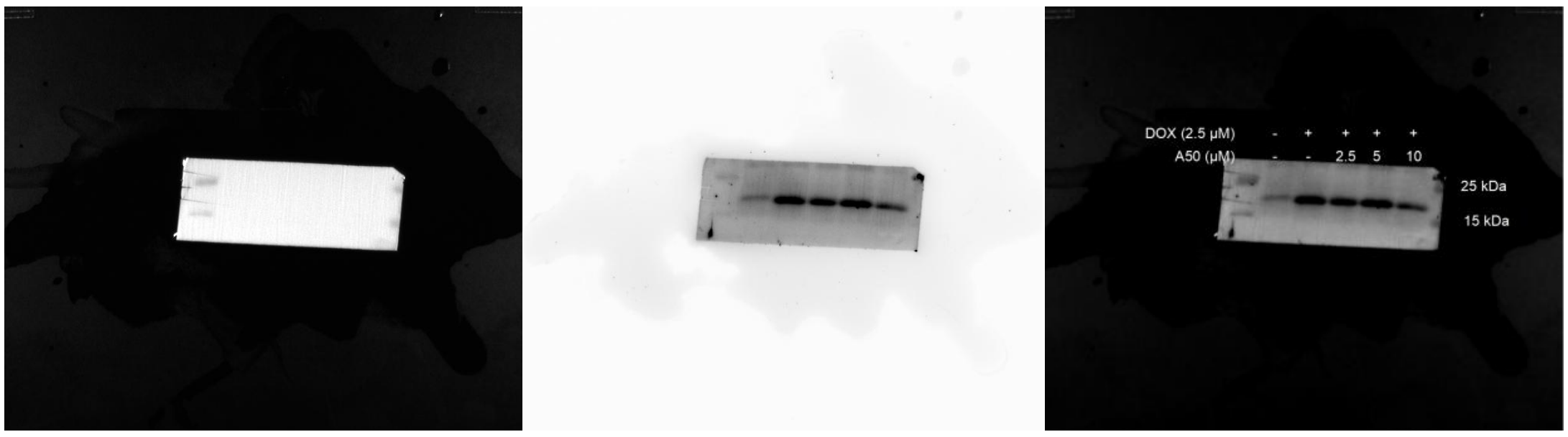

β-Tublin

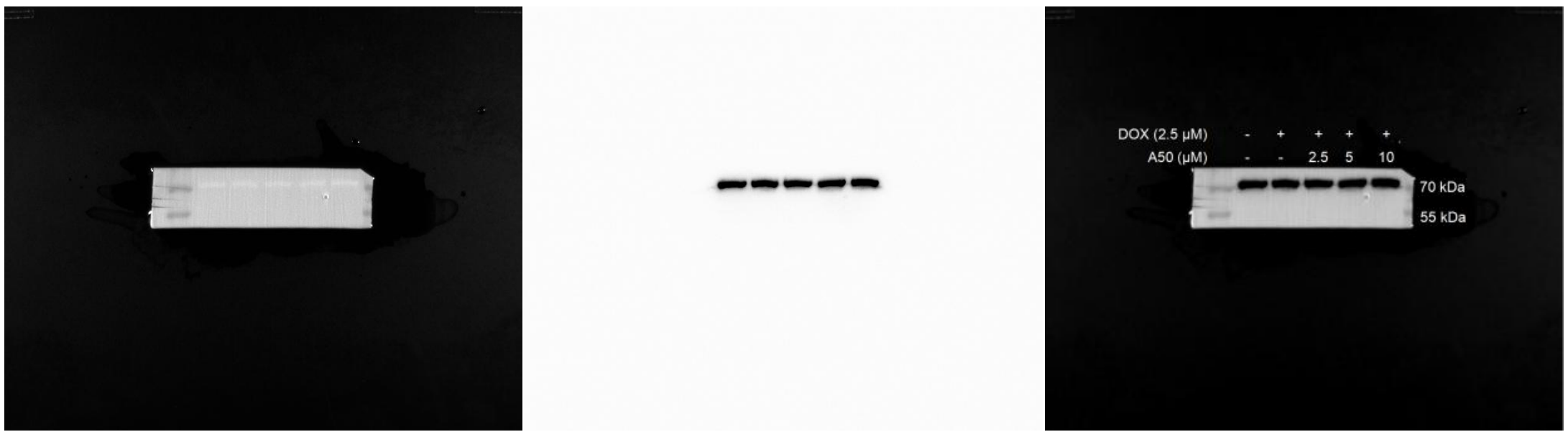

Full-size

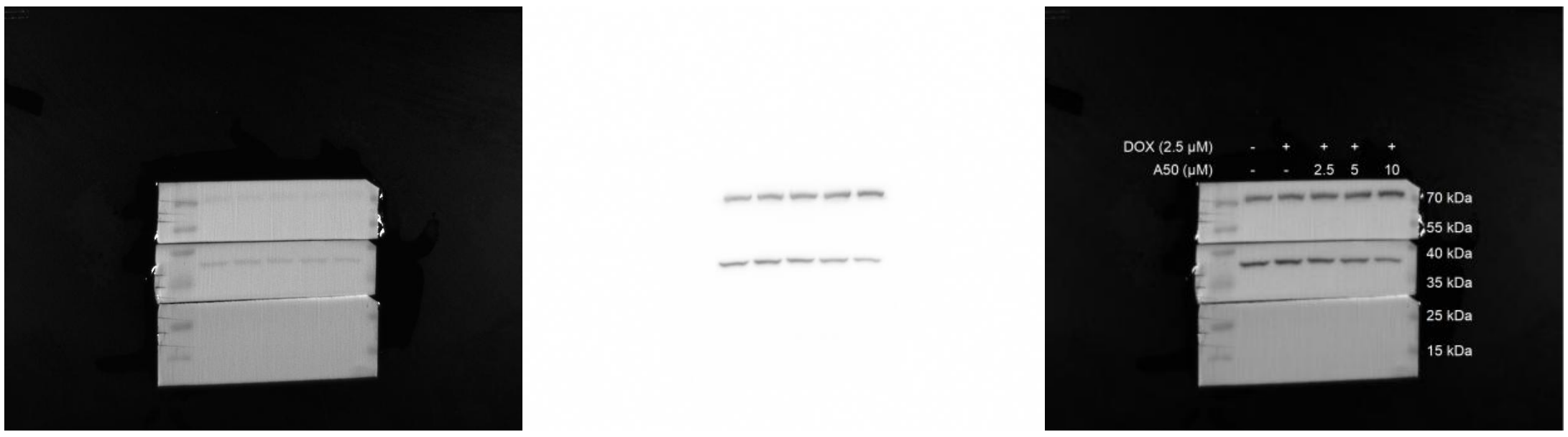

(3) Caspase-3

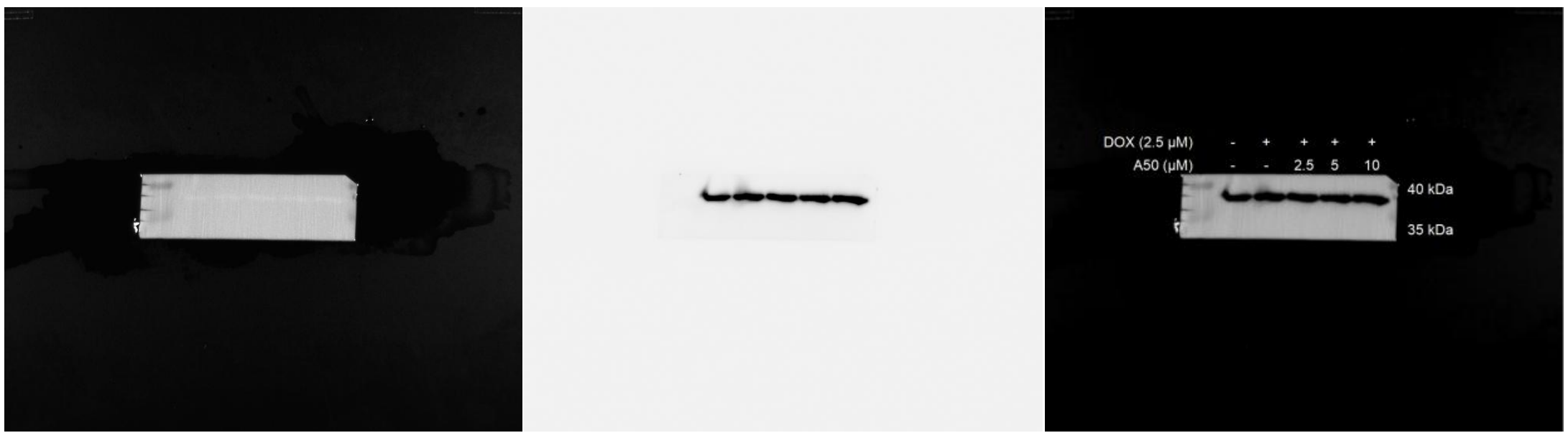

Cleaved-Caspase-3

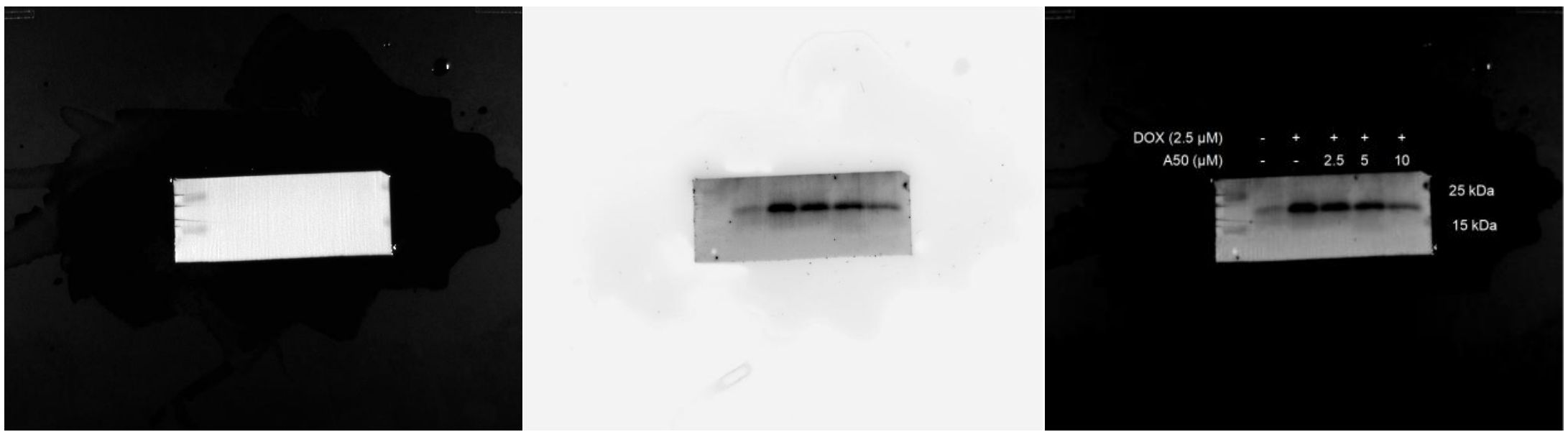

β-Tublin

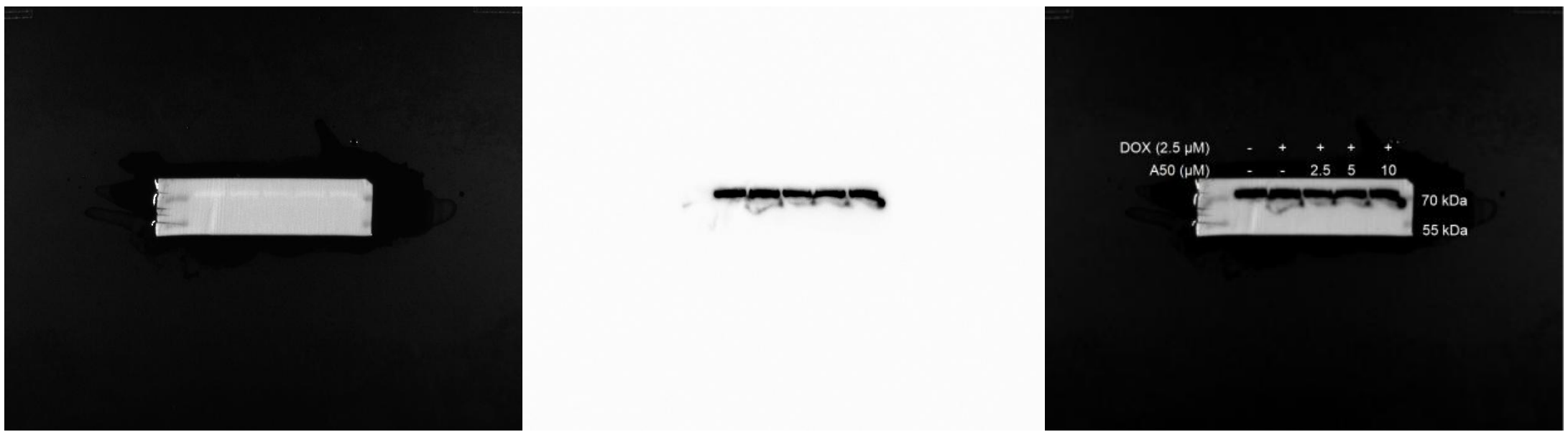

Full-size

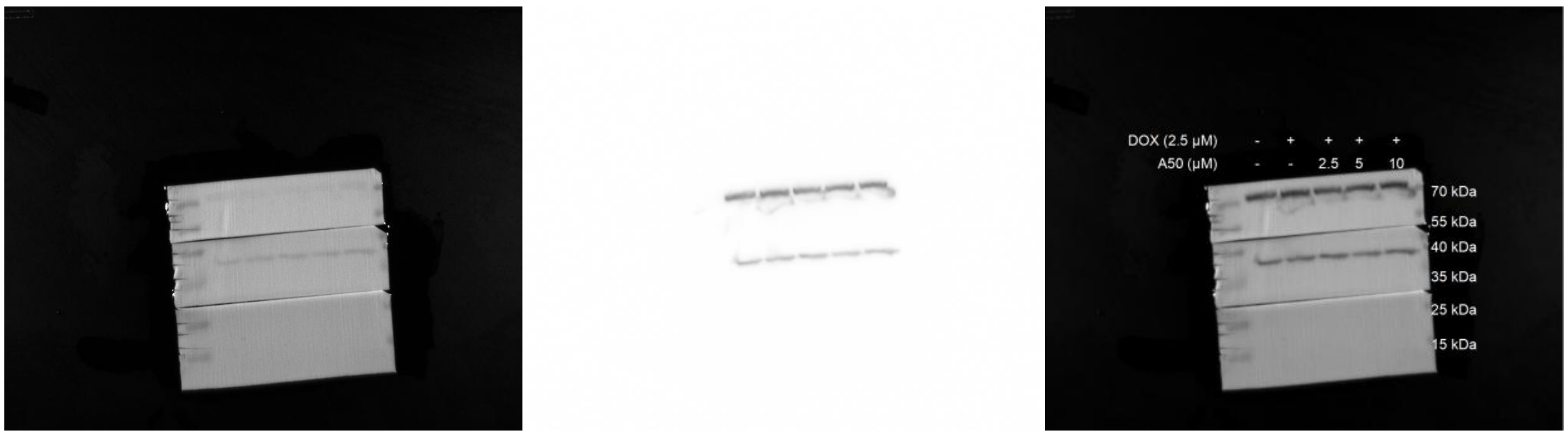

Western blot of HO-1, PI3K, p-PI3K, AKT and p-AKT (Figure 6, A. Repeat 1-3)

1. (1) HO-1

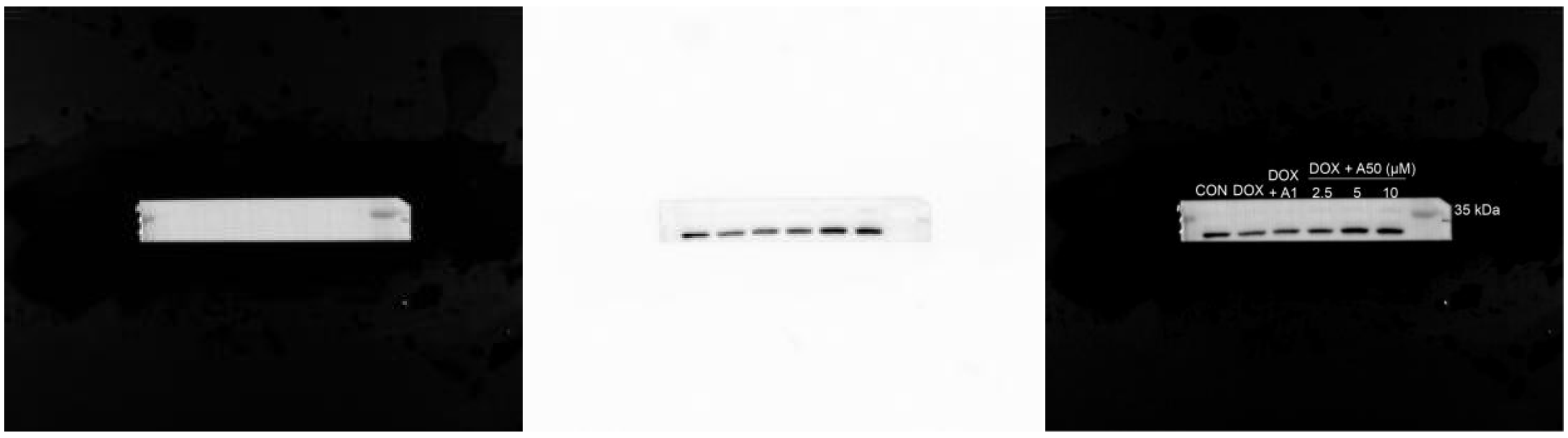

β-actin

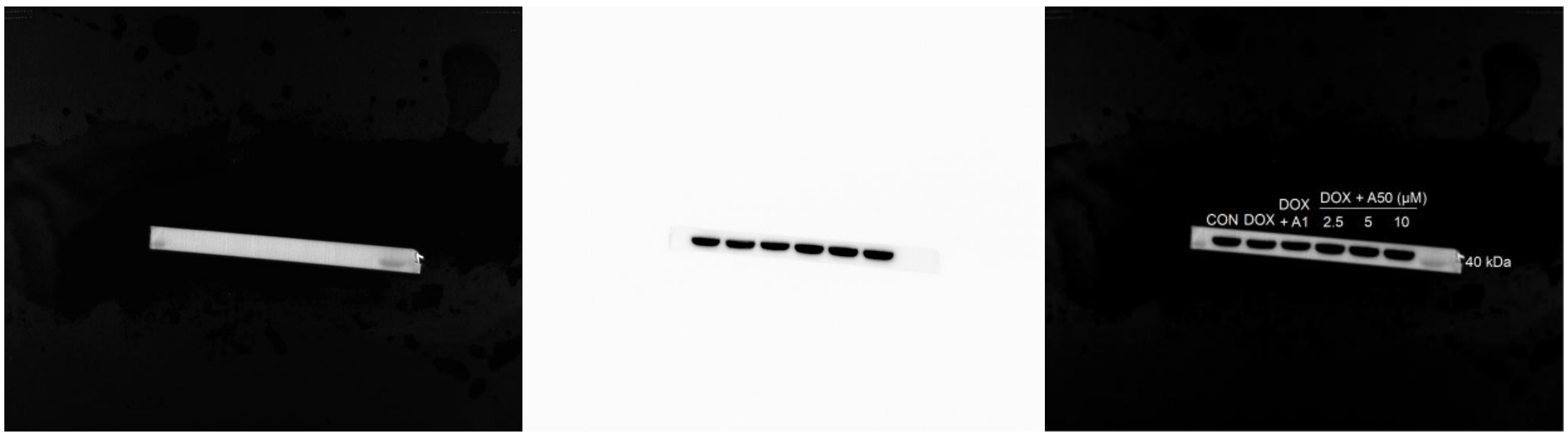

Full-size

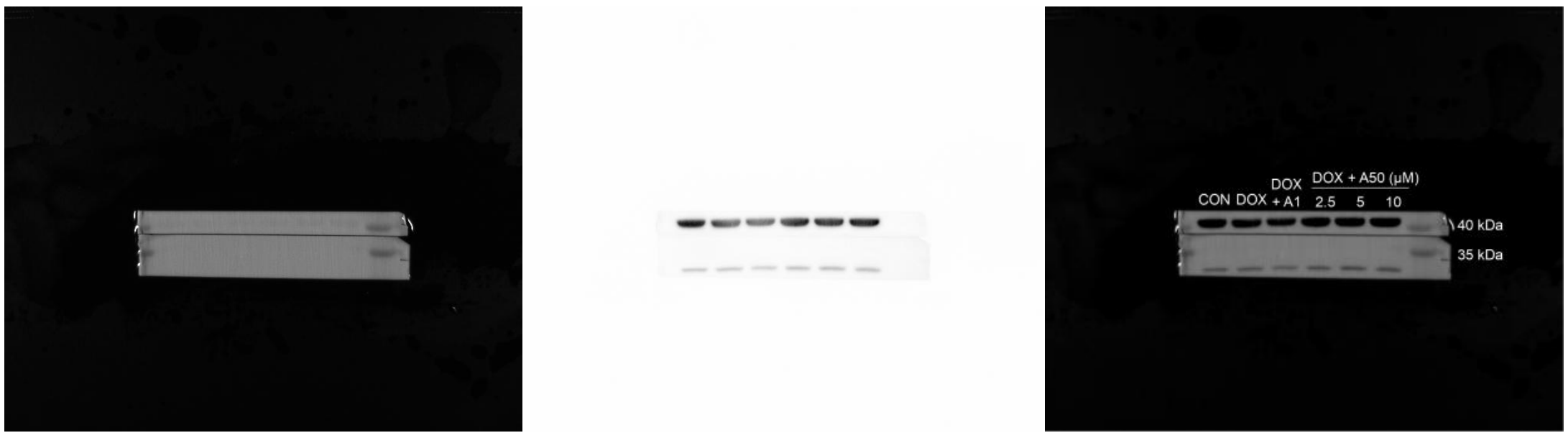

(2) HO-1

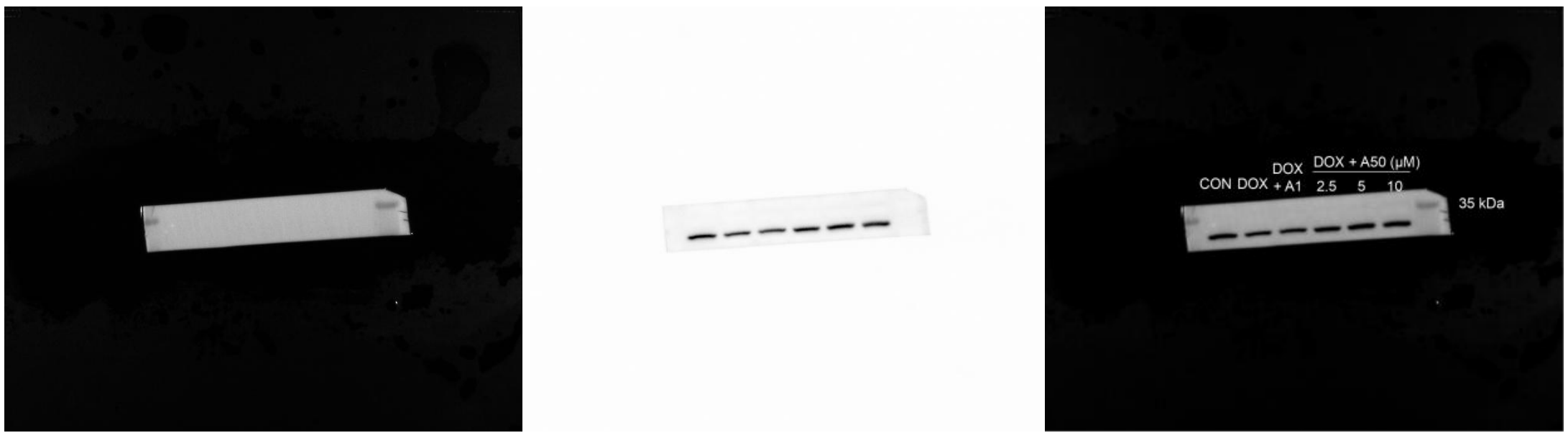

β-actin

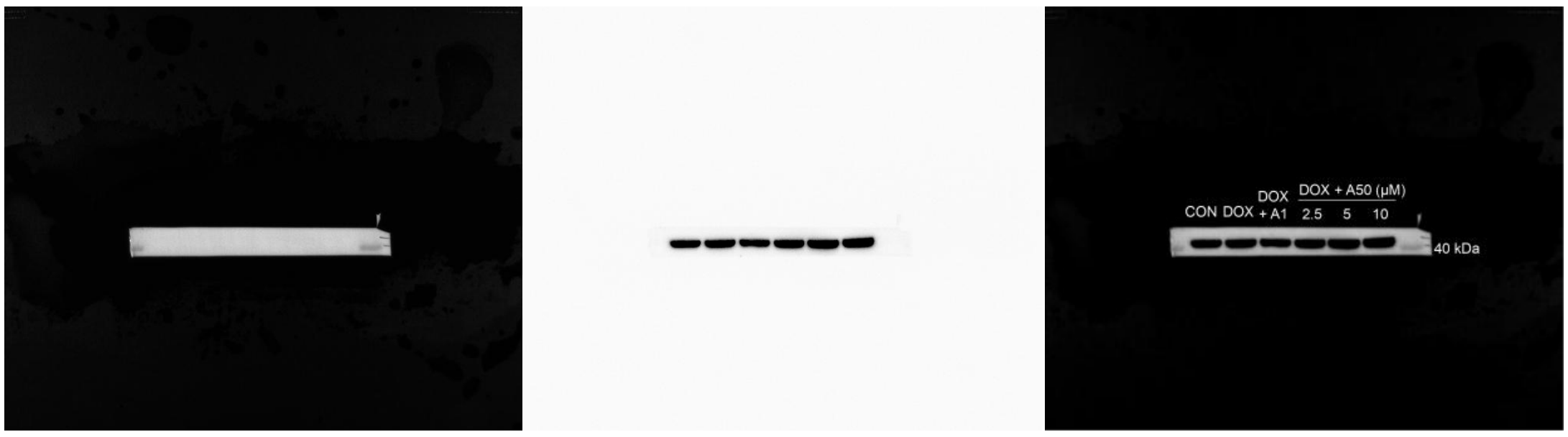

Full-size

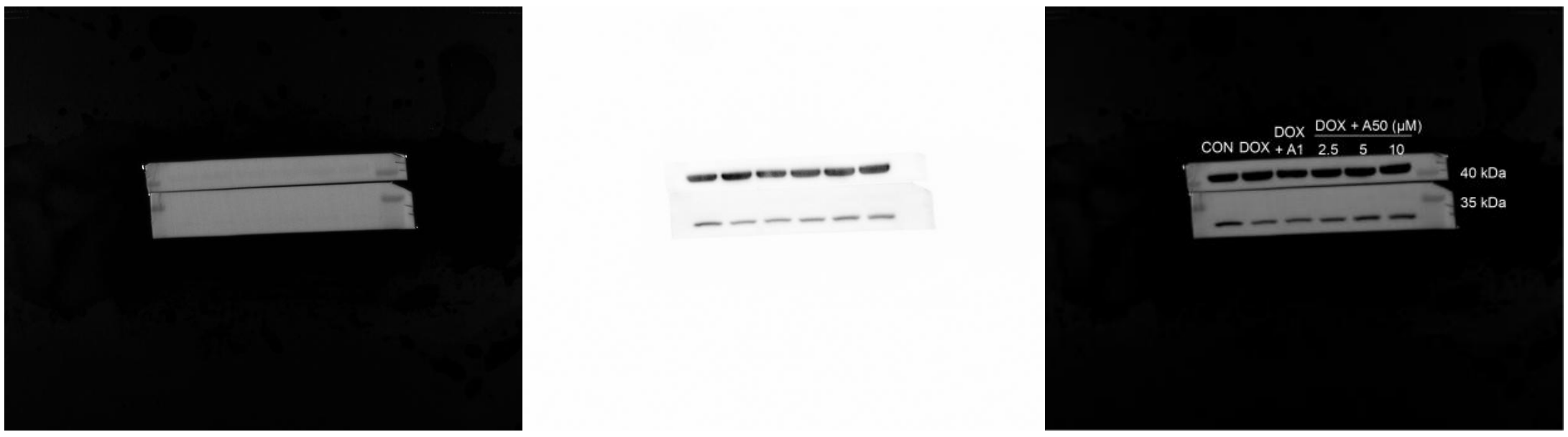

(3) HO-1

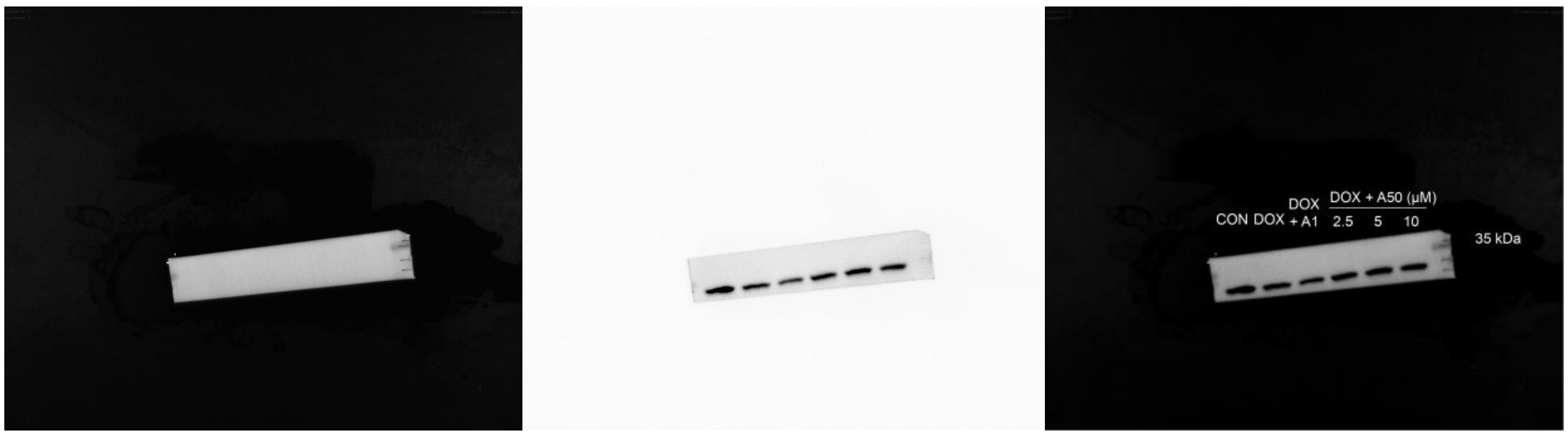

β-actin

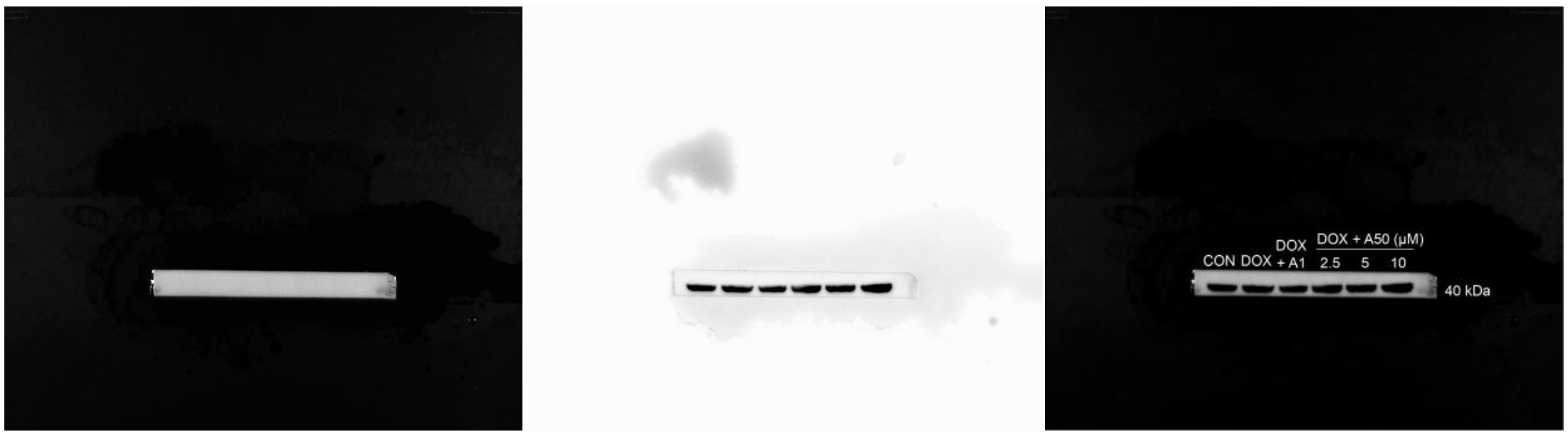

Full-size

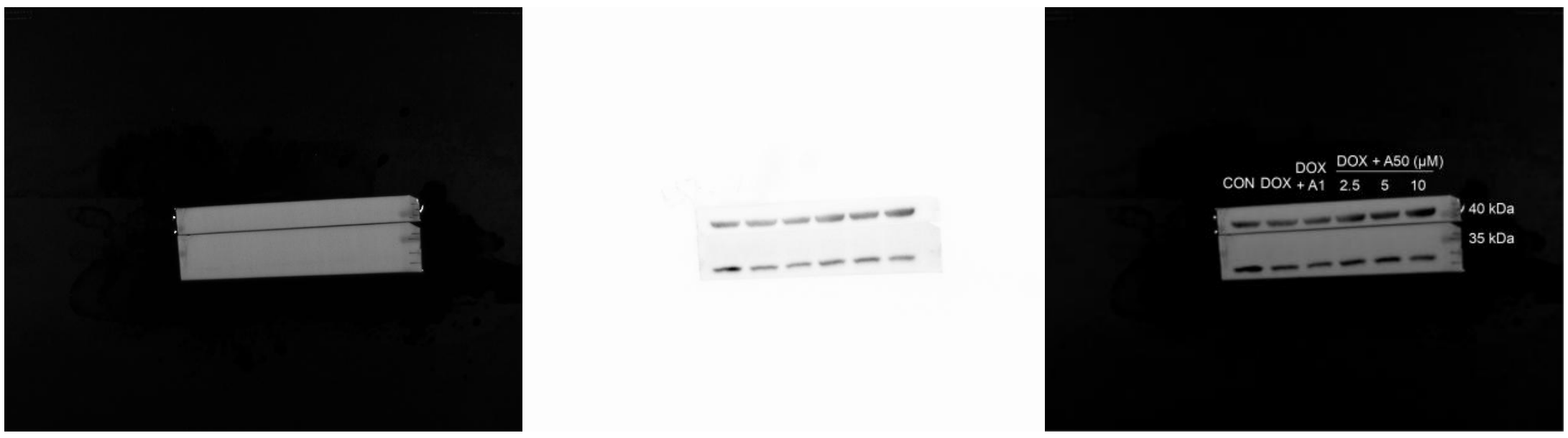

2. (1) PI3K

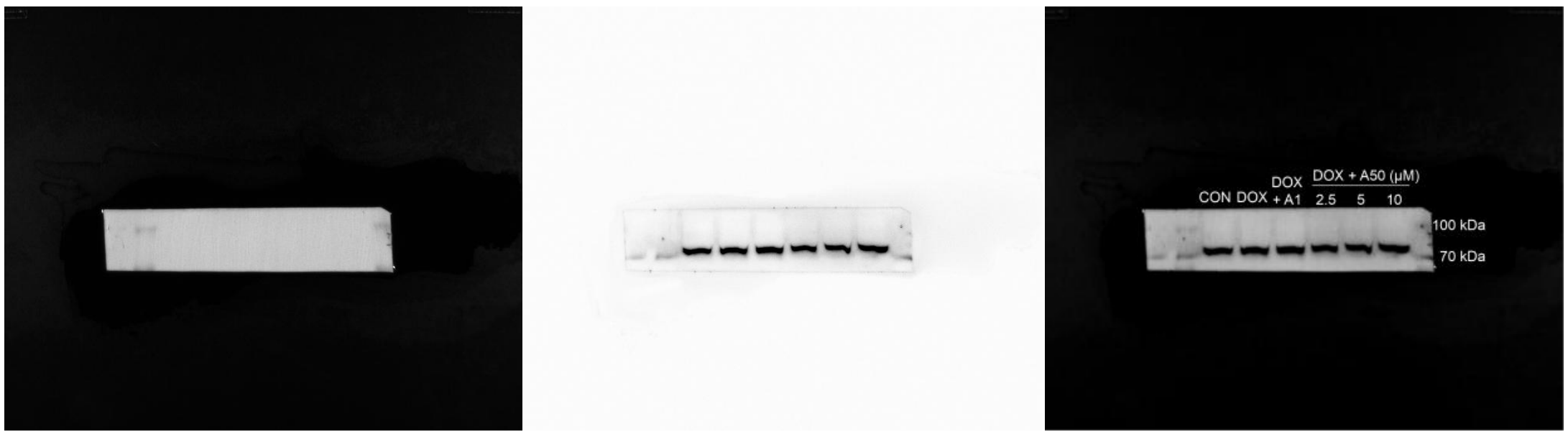

AKT

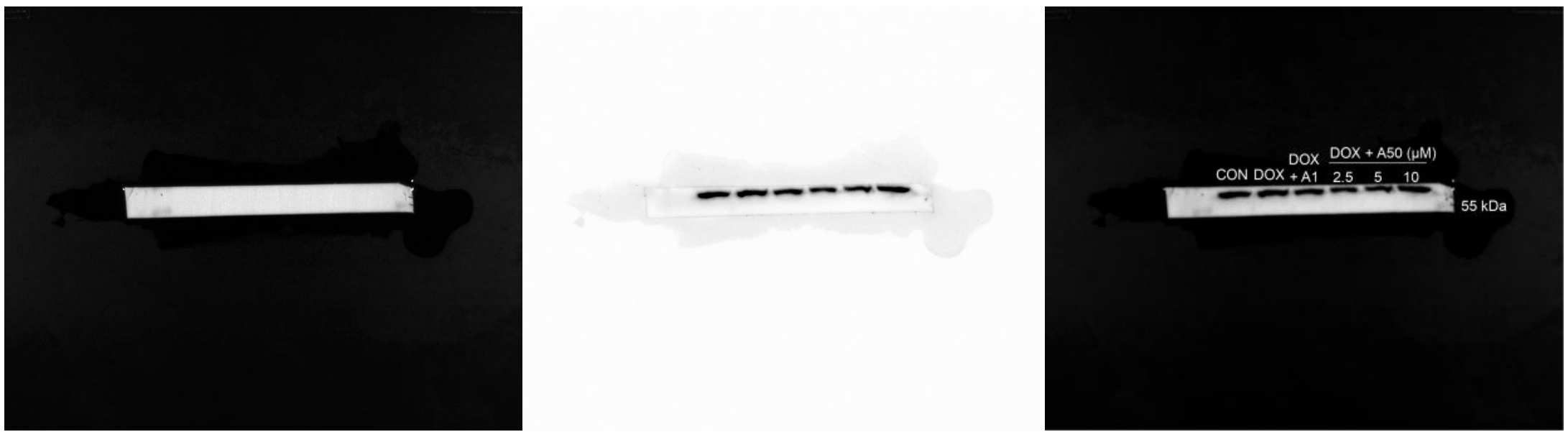

β-actin

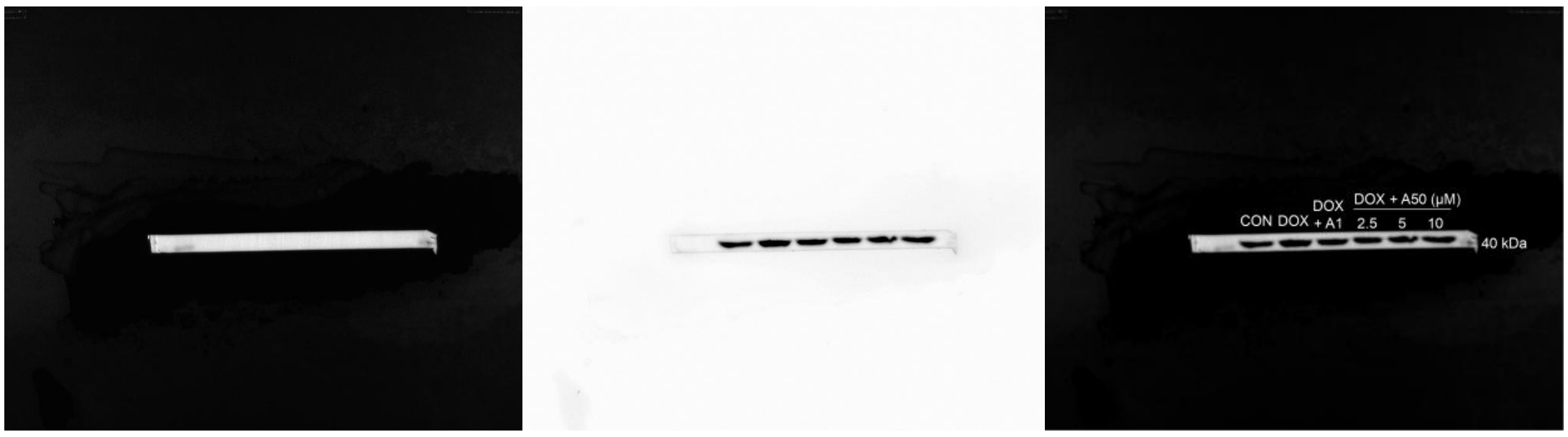

Full-size

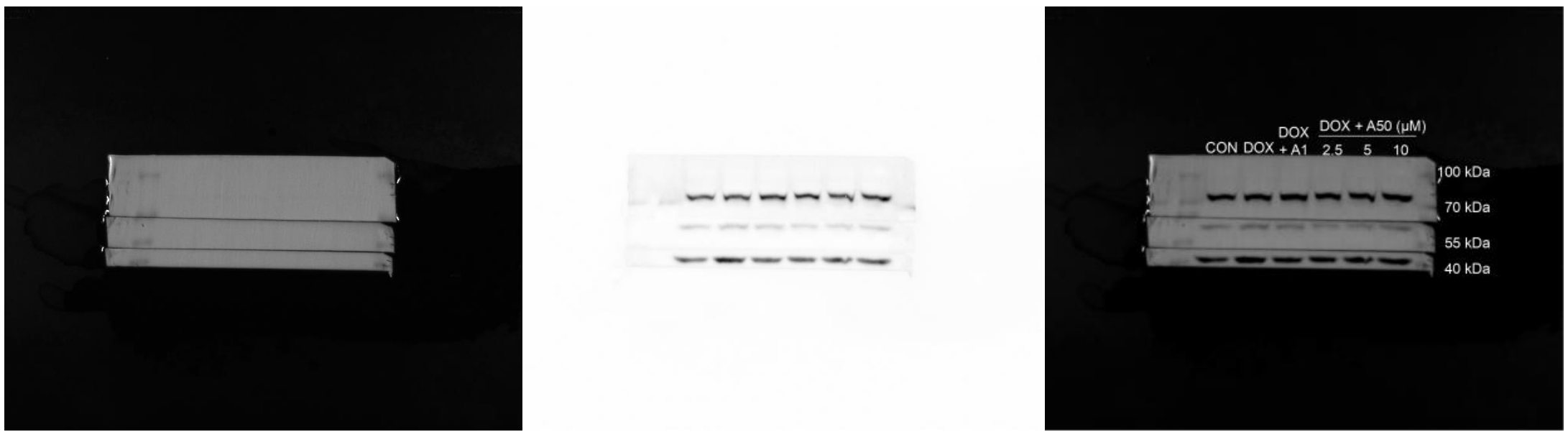

(2) PI3K

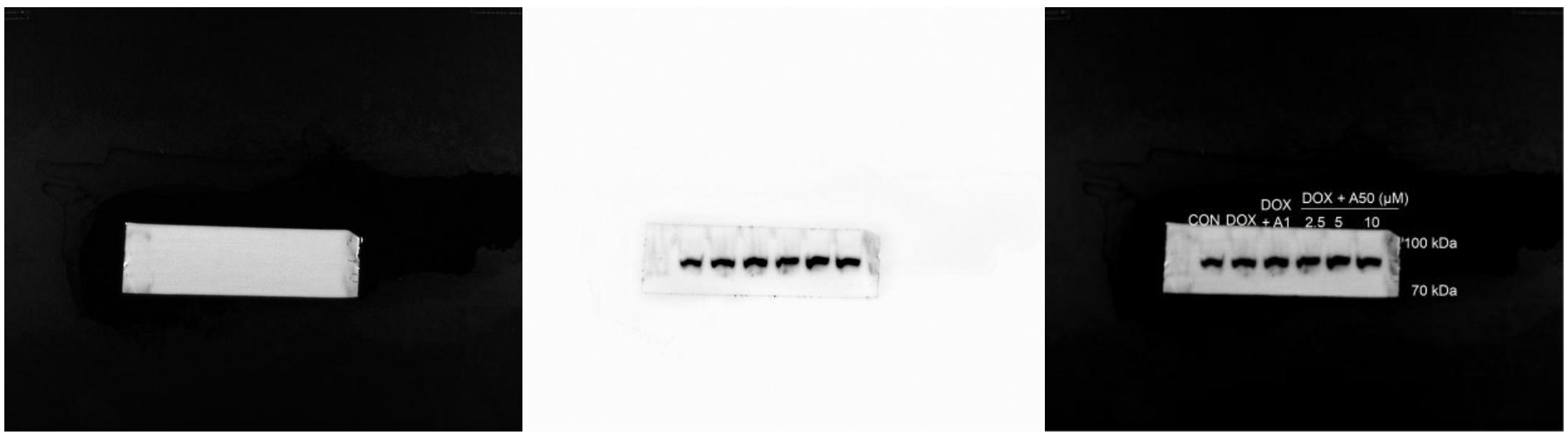

AKT

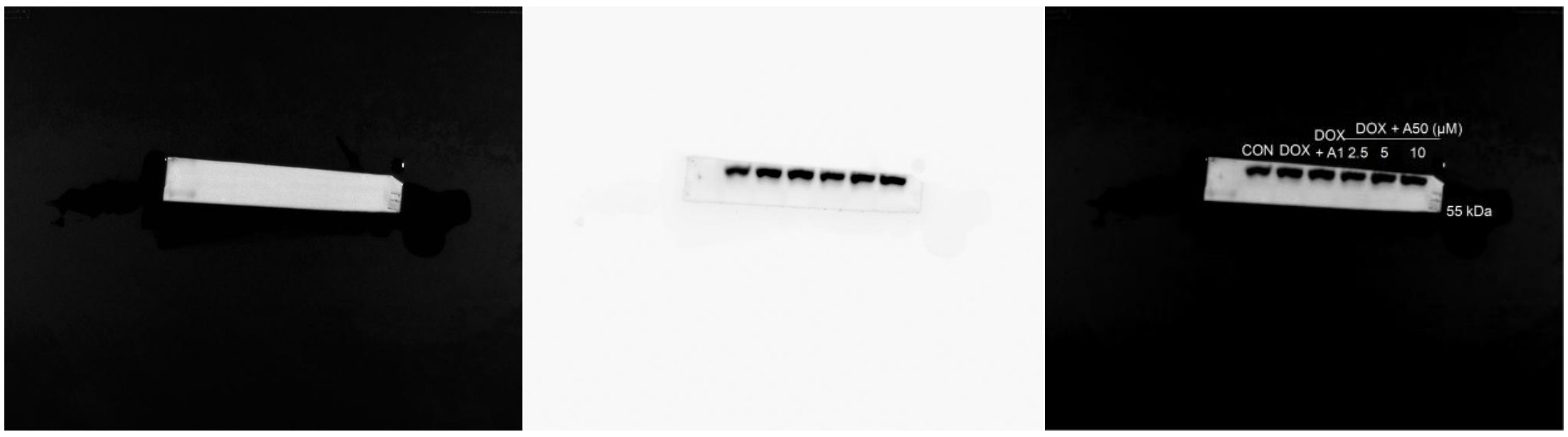

β-actin

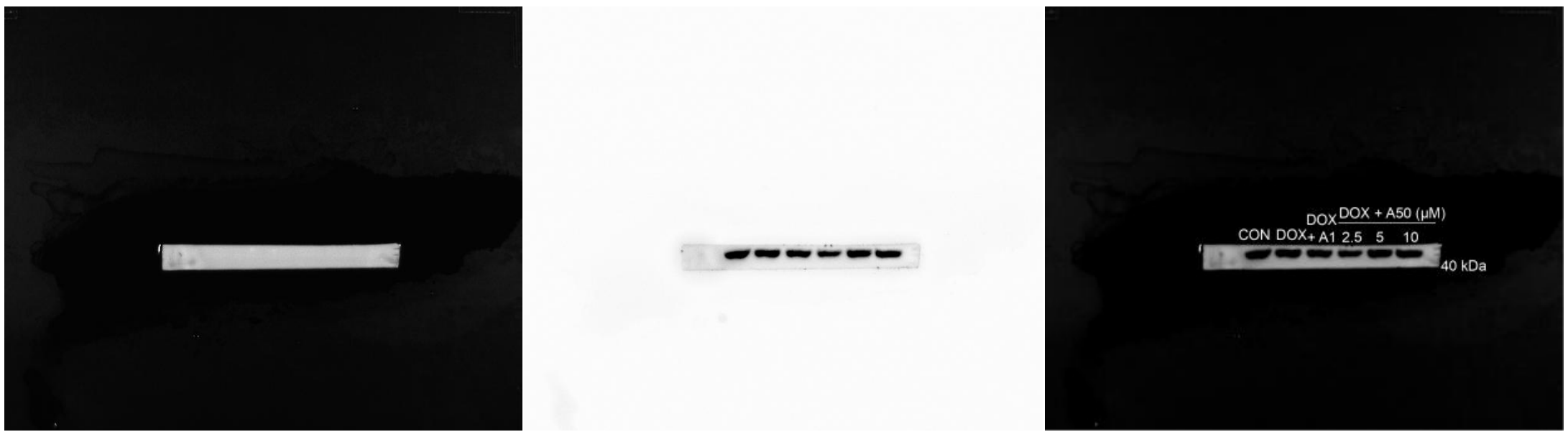

Full-size

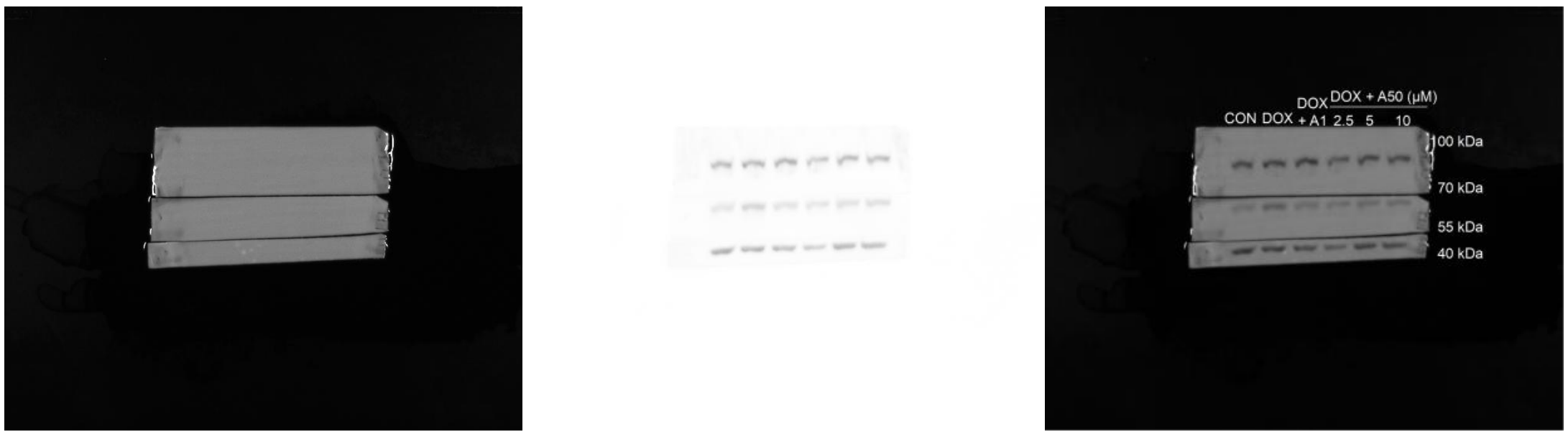

(3) PI3K

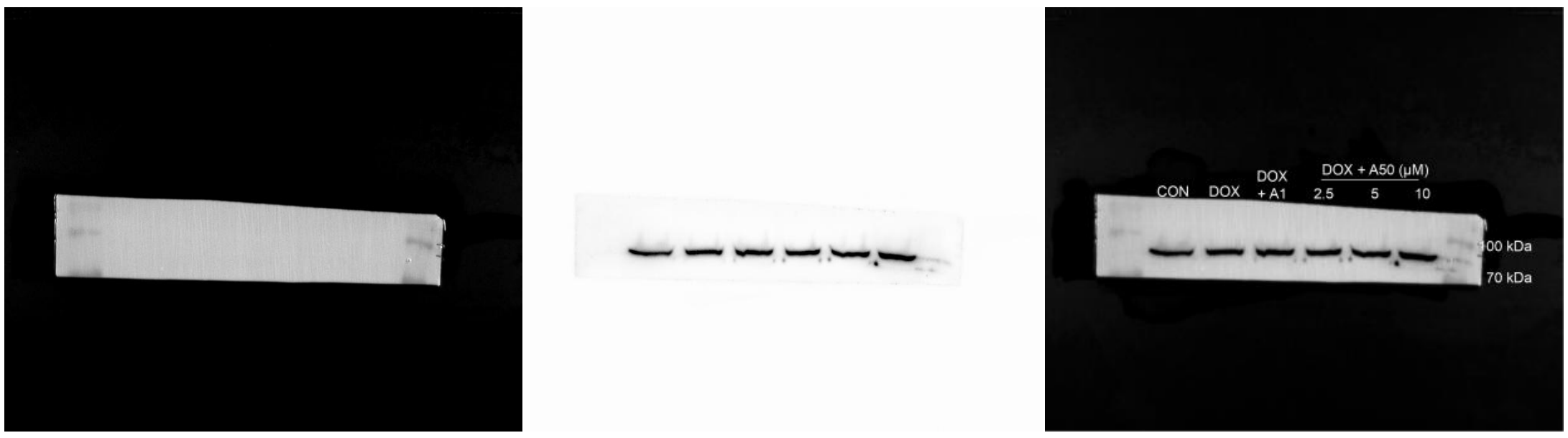

AKT

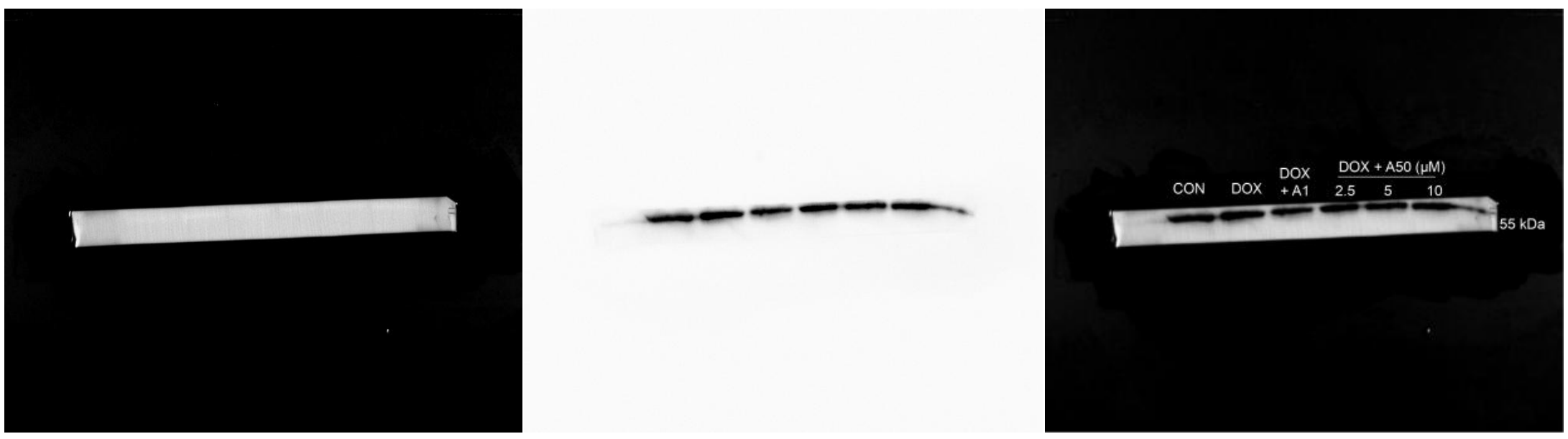

β-actin

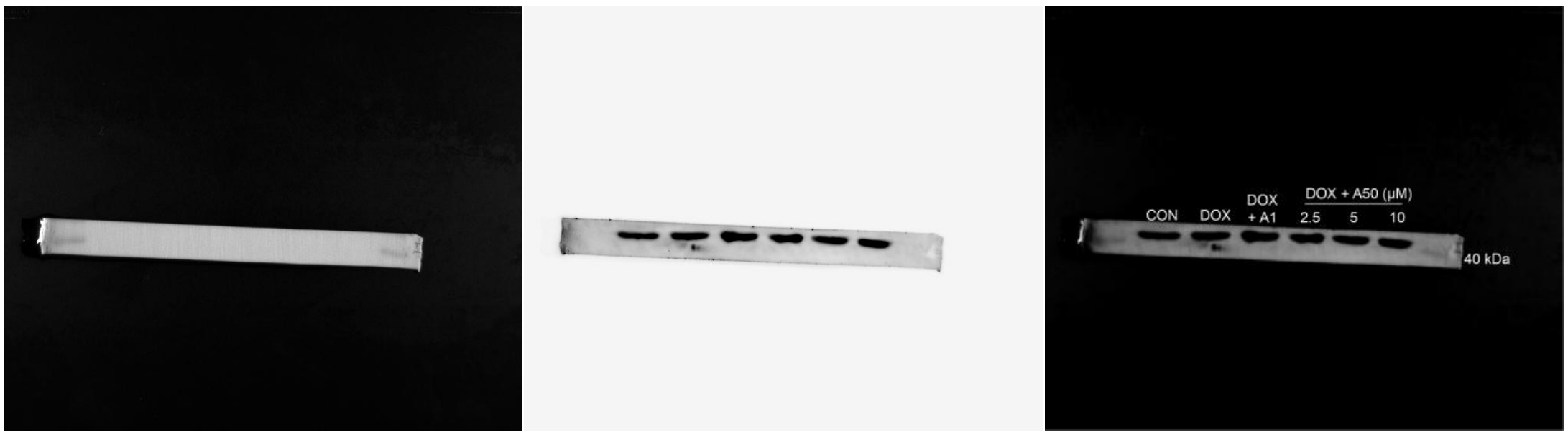

Full-size

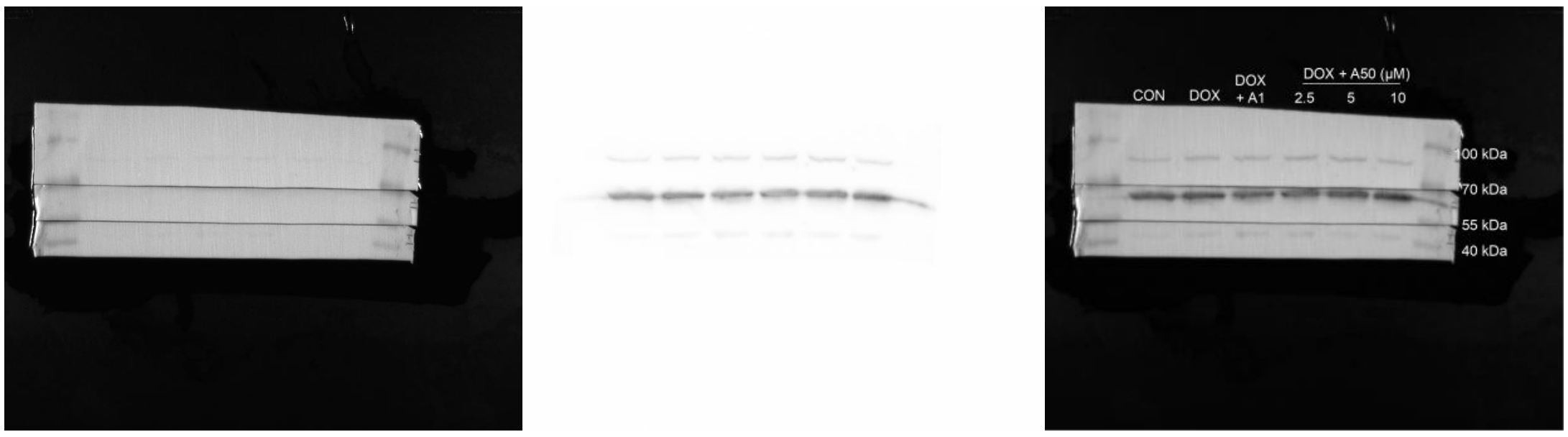

3. (1) p-PI3K

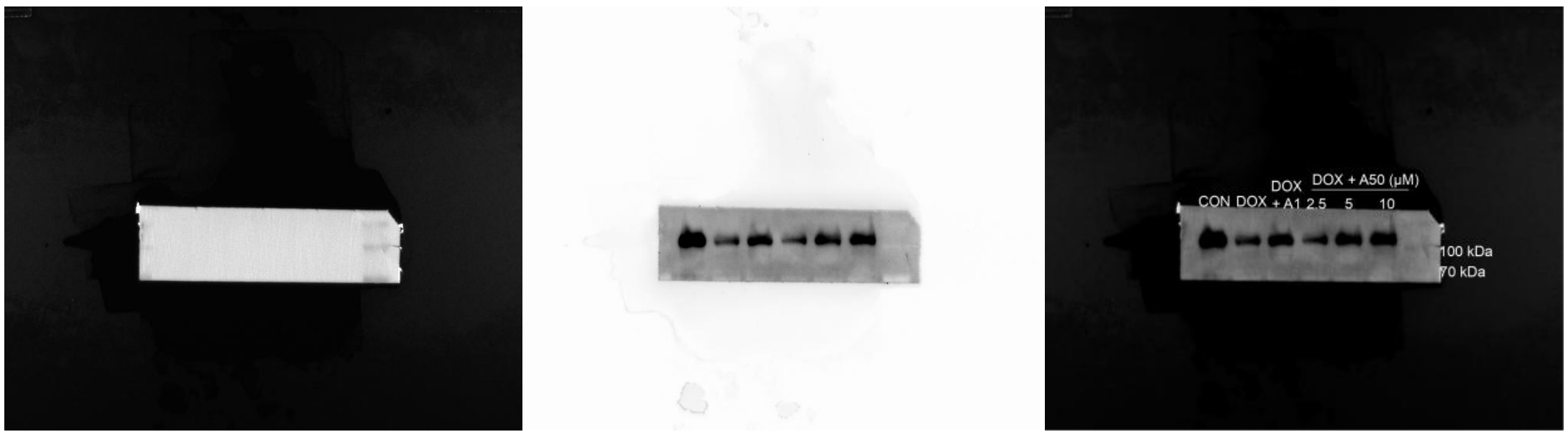

β-actin

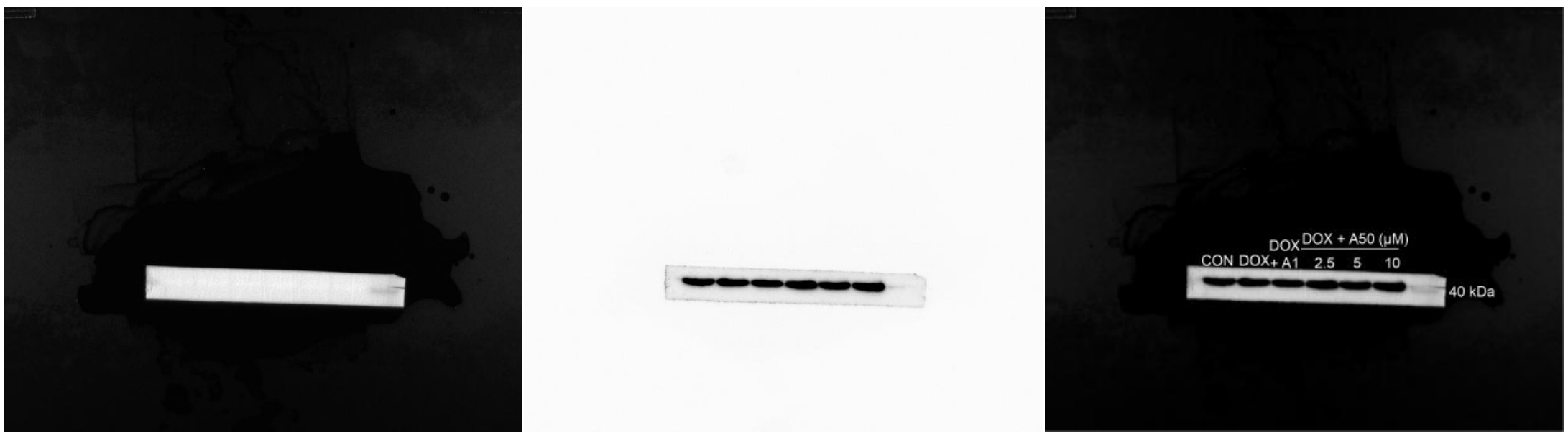

Full-size

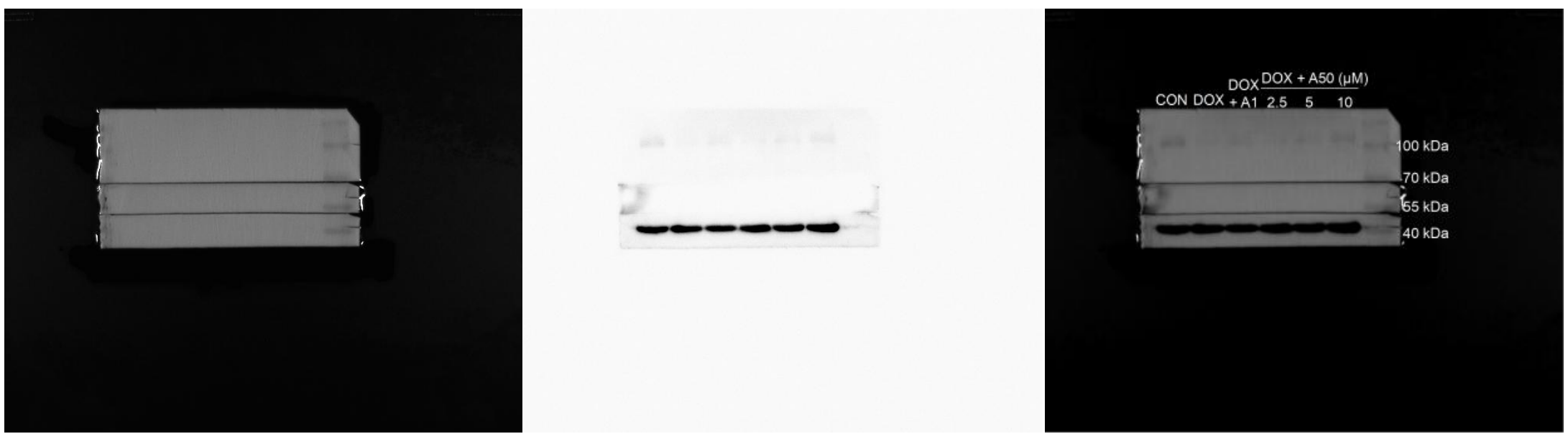

(2) p-PI3K

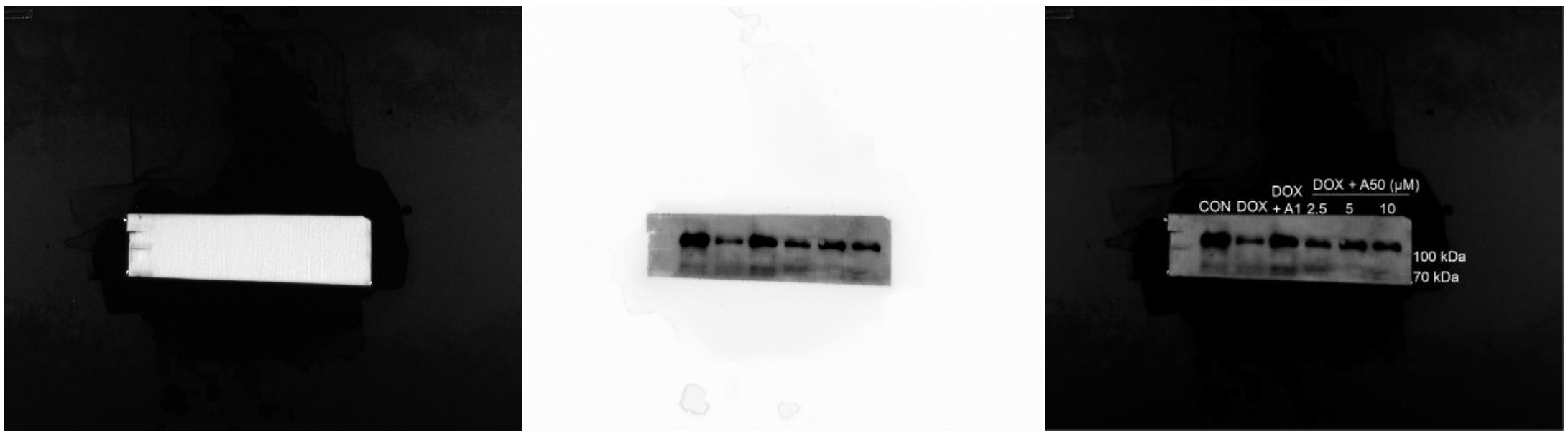

β-actin

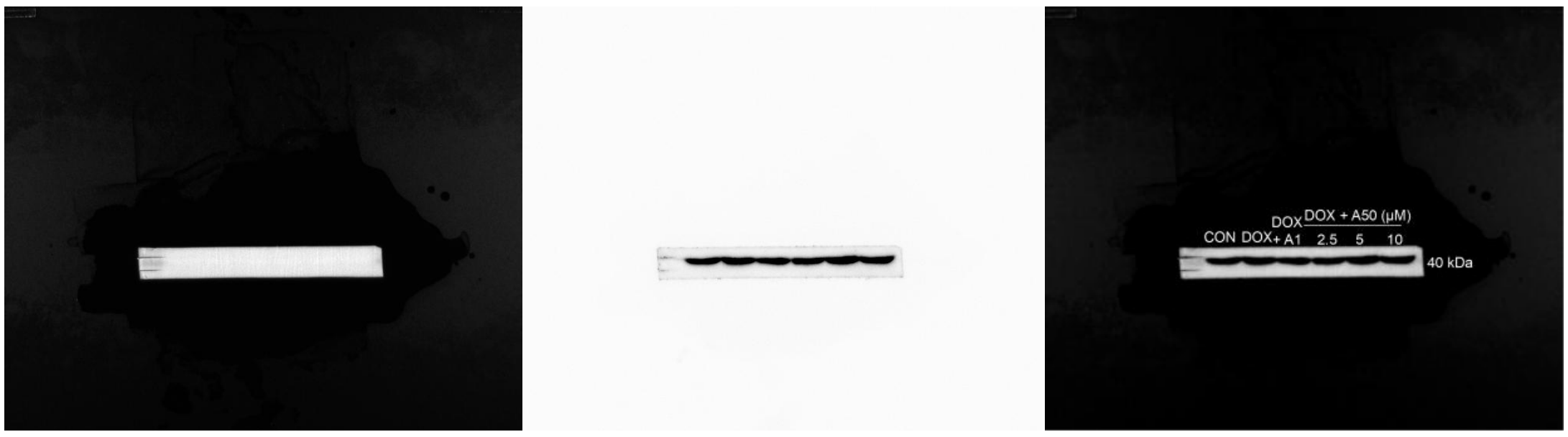

Full-size

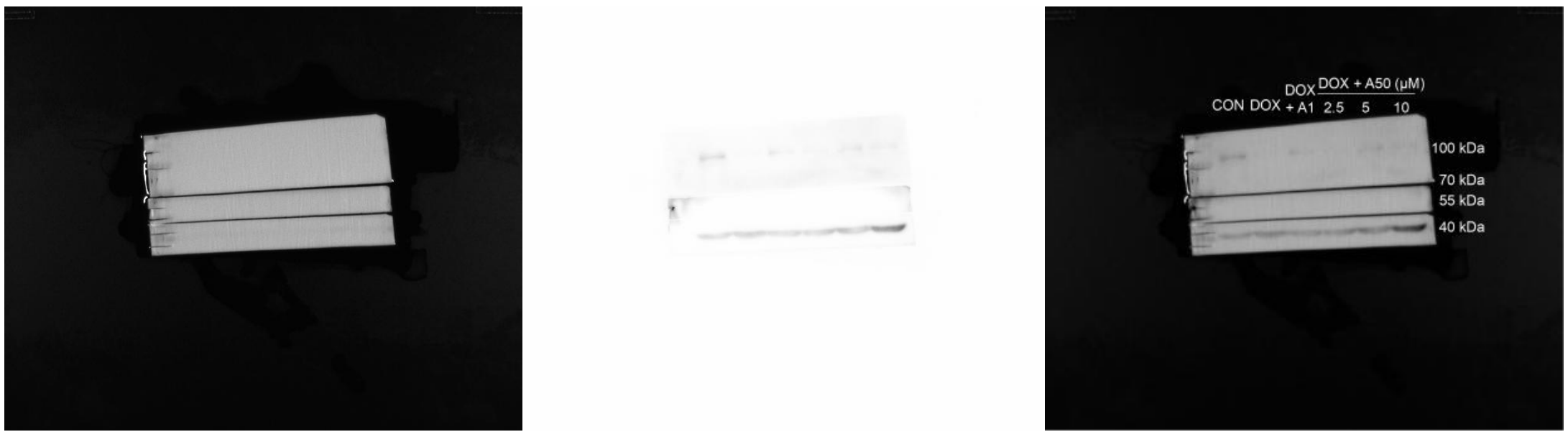

(3) p-PI3K

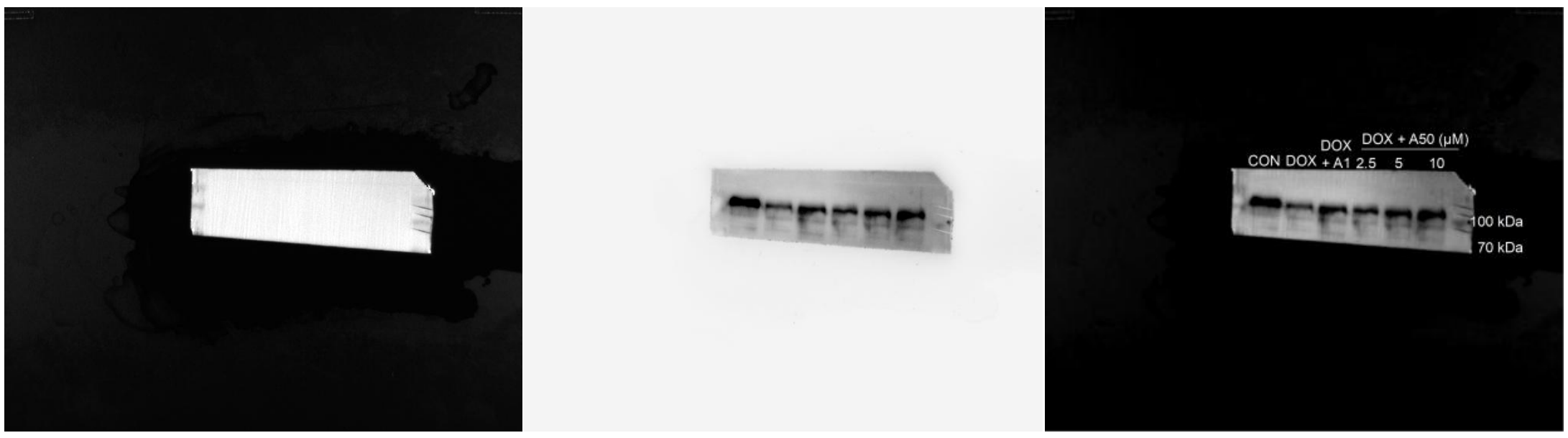

β-actin

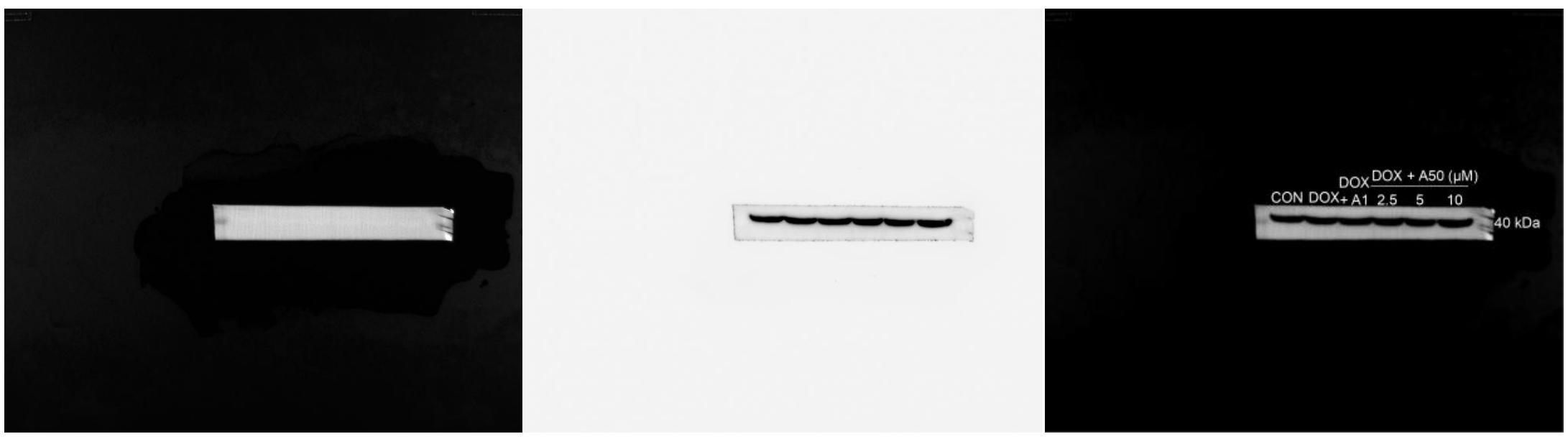

Full-size

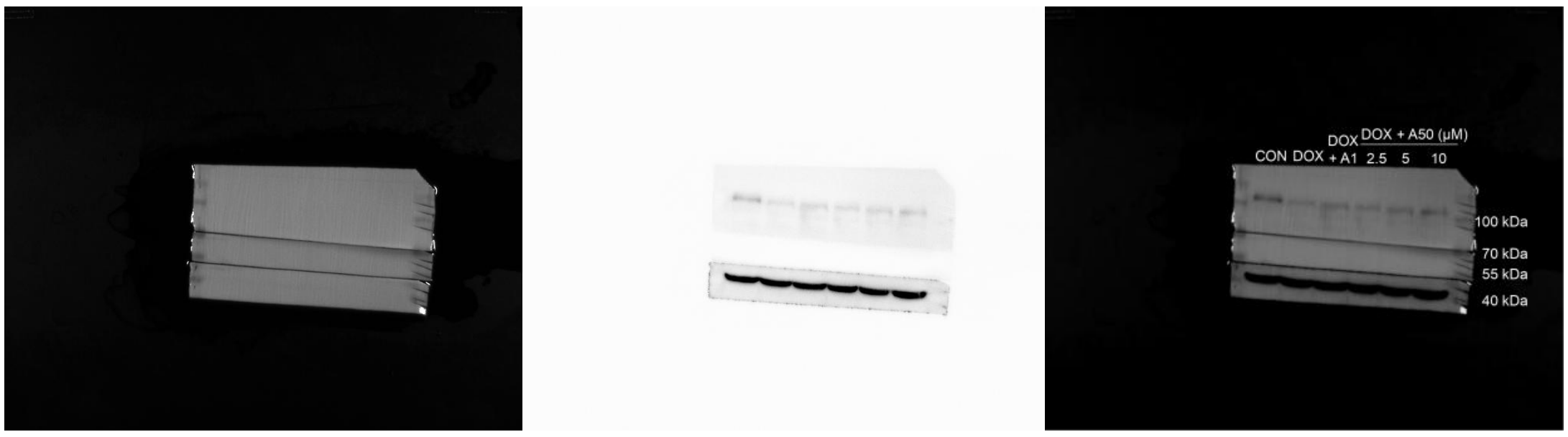

4. (1) p-AKT

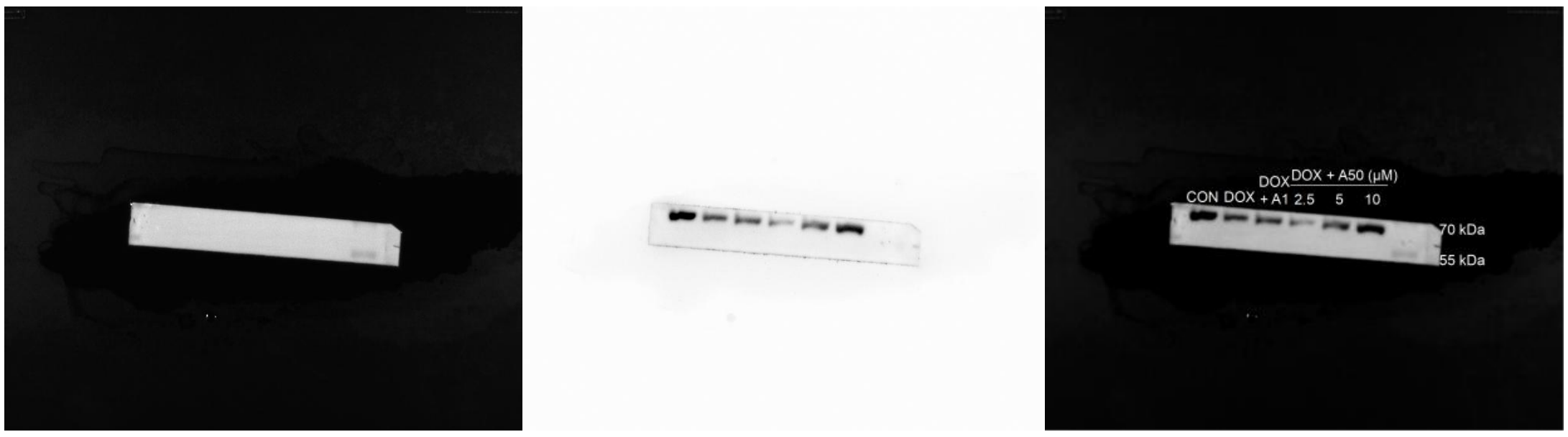

β-actin

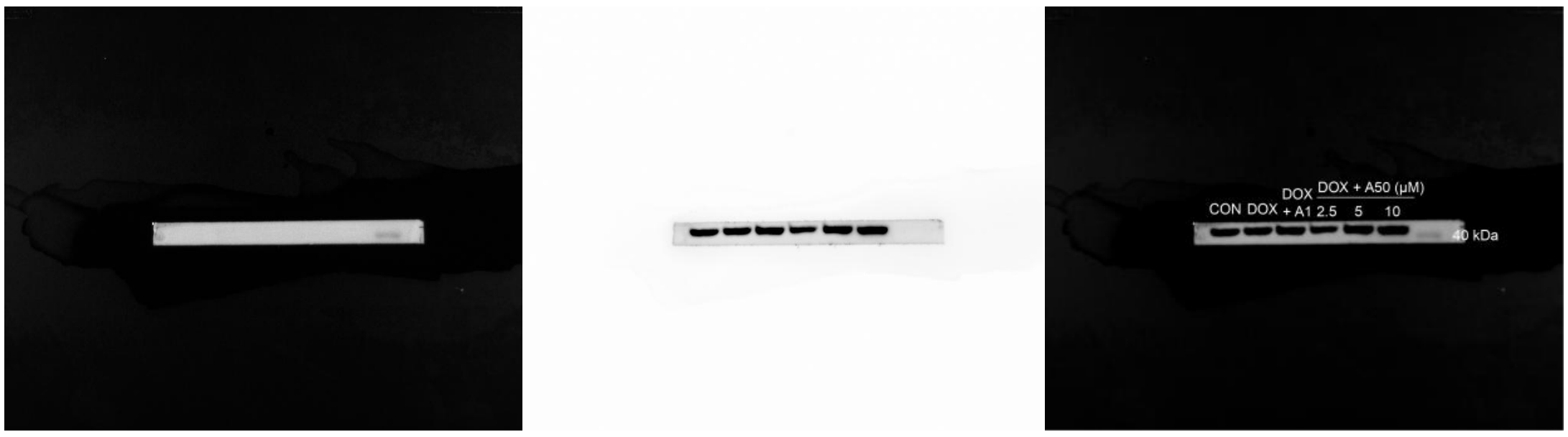

Full-size

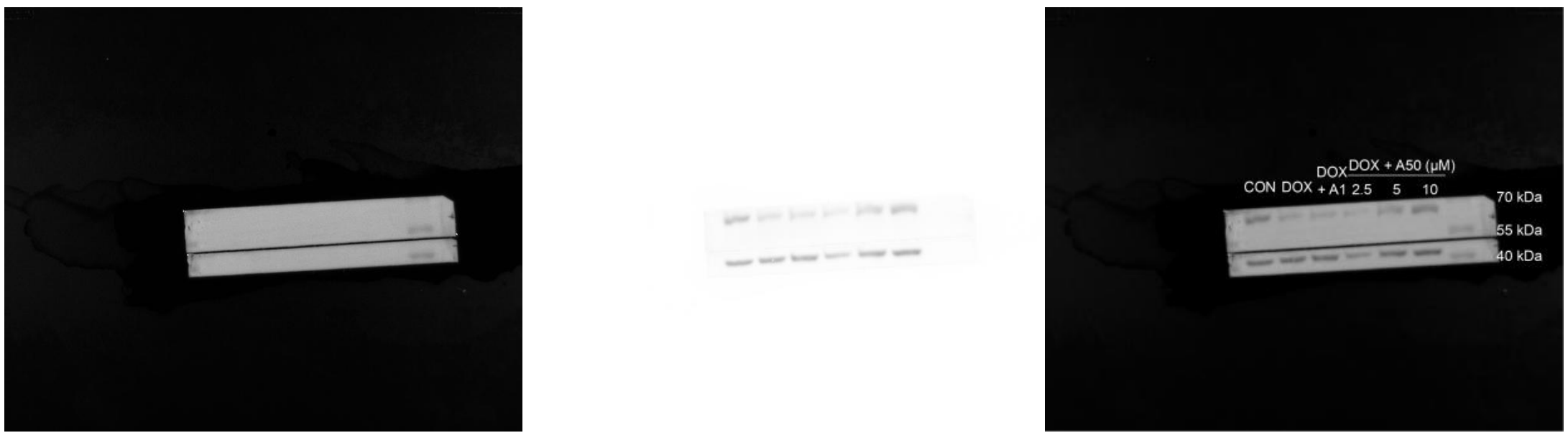

(2) p-AKT

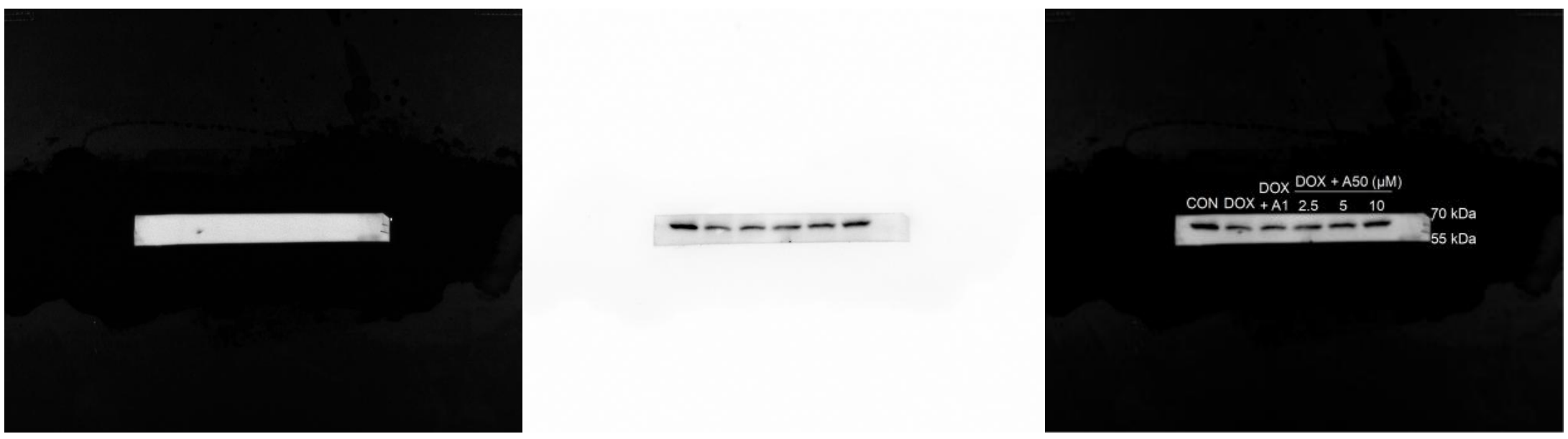

β-actin

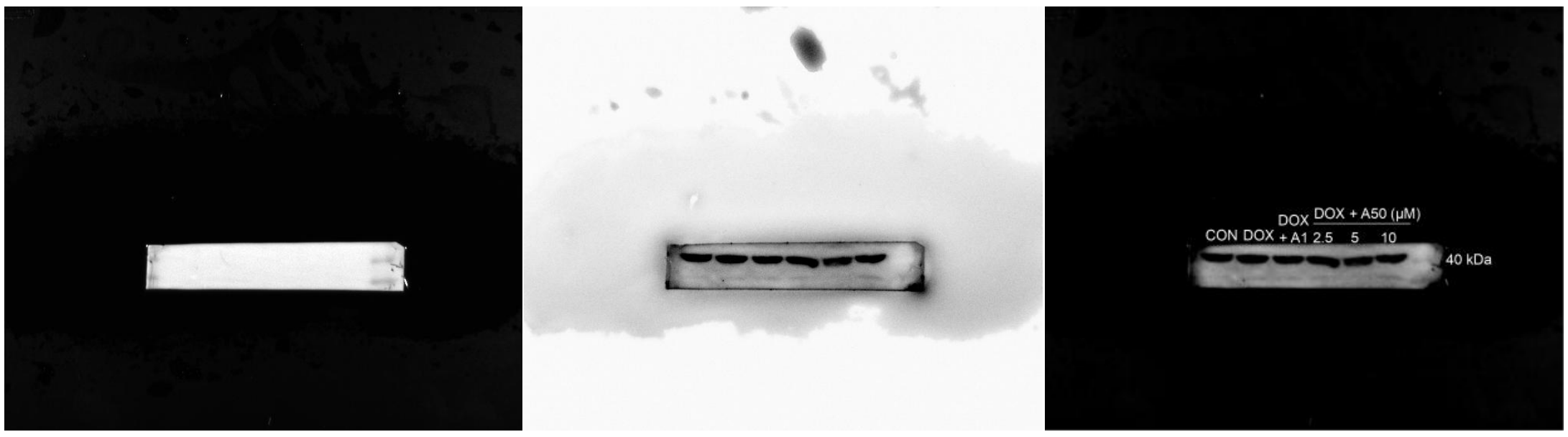

Full-size

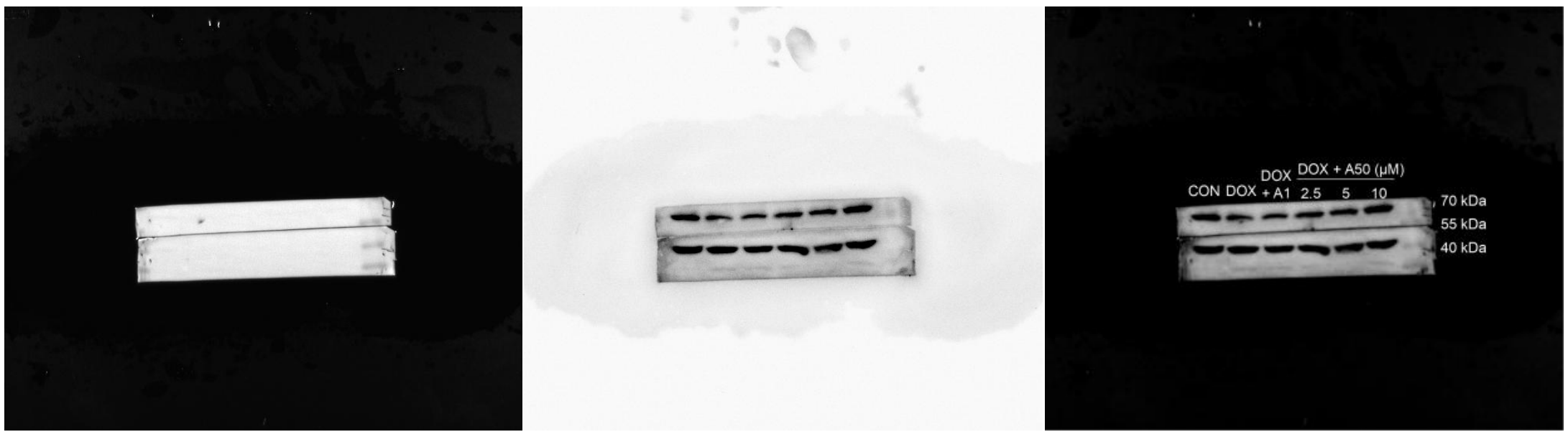

(3) p-AKT

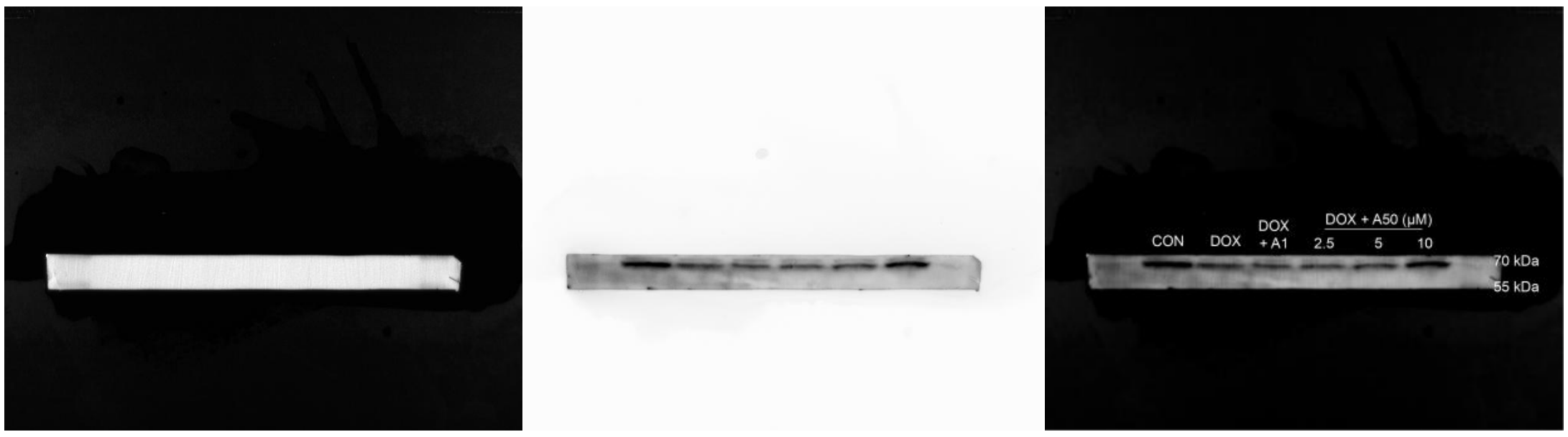

β-actin

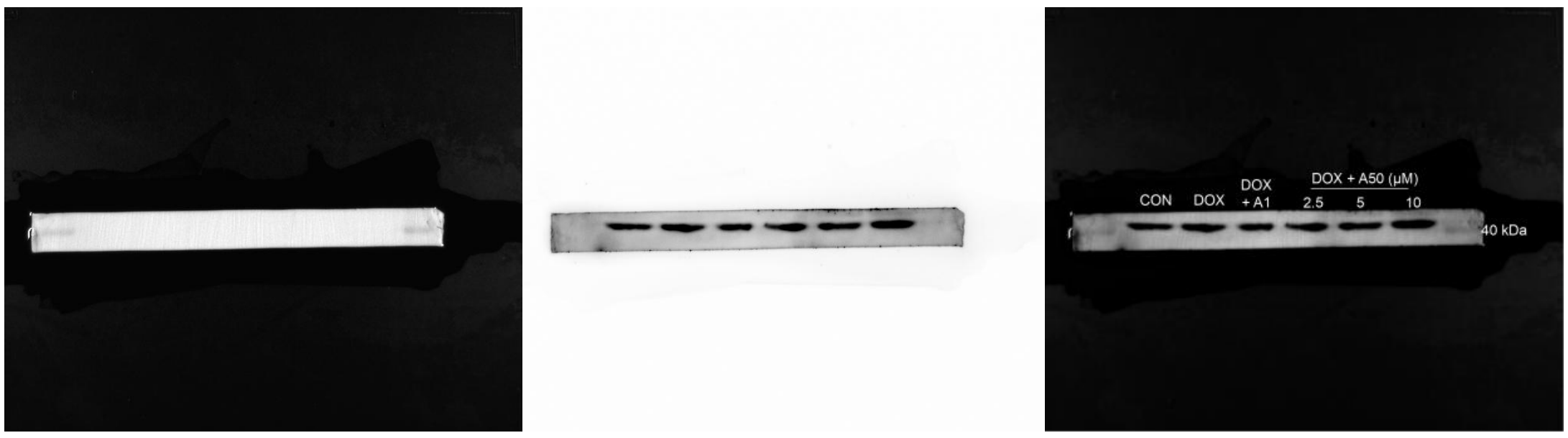

Full-size

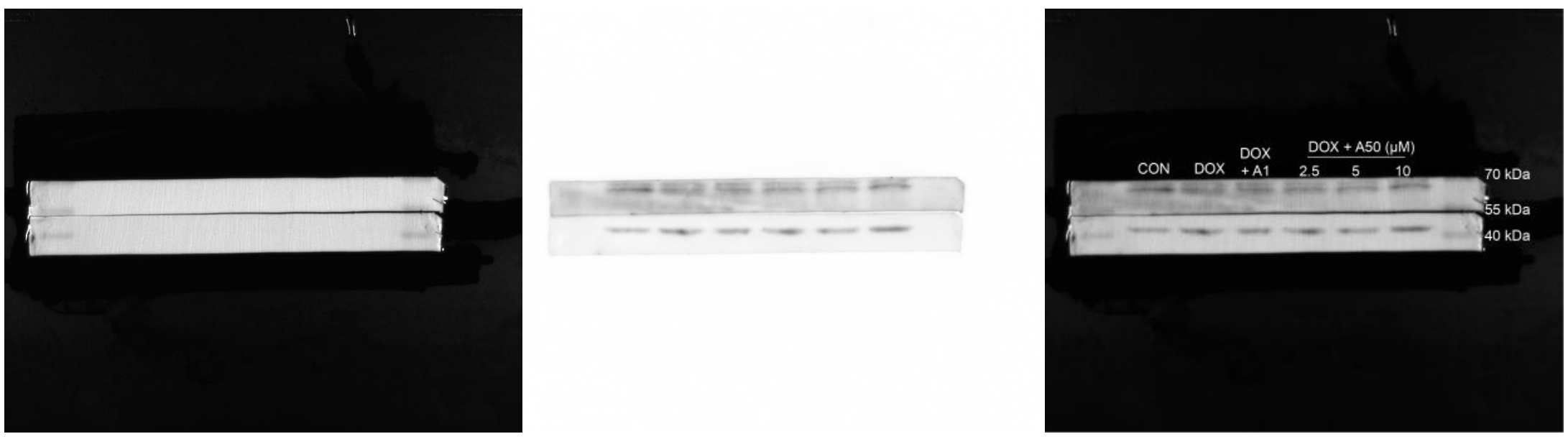

Western blot of PI3K, p-PI3K, AKT, p-AKT, β-Actin (Figure 6, E. Repeat 1-3)

1. (1) HO-1

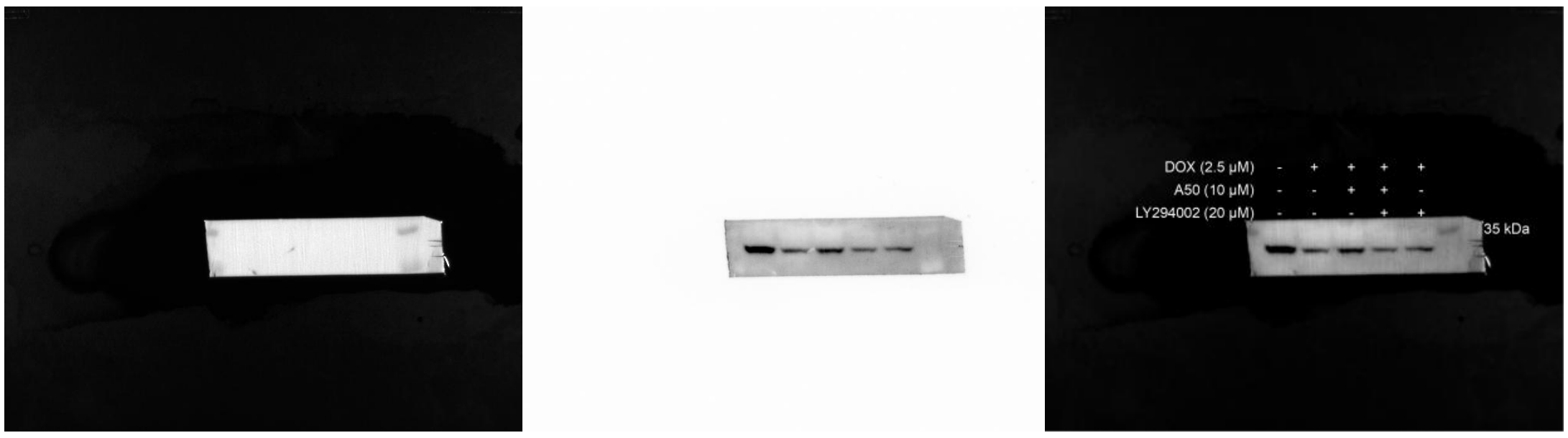

β-actin

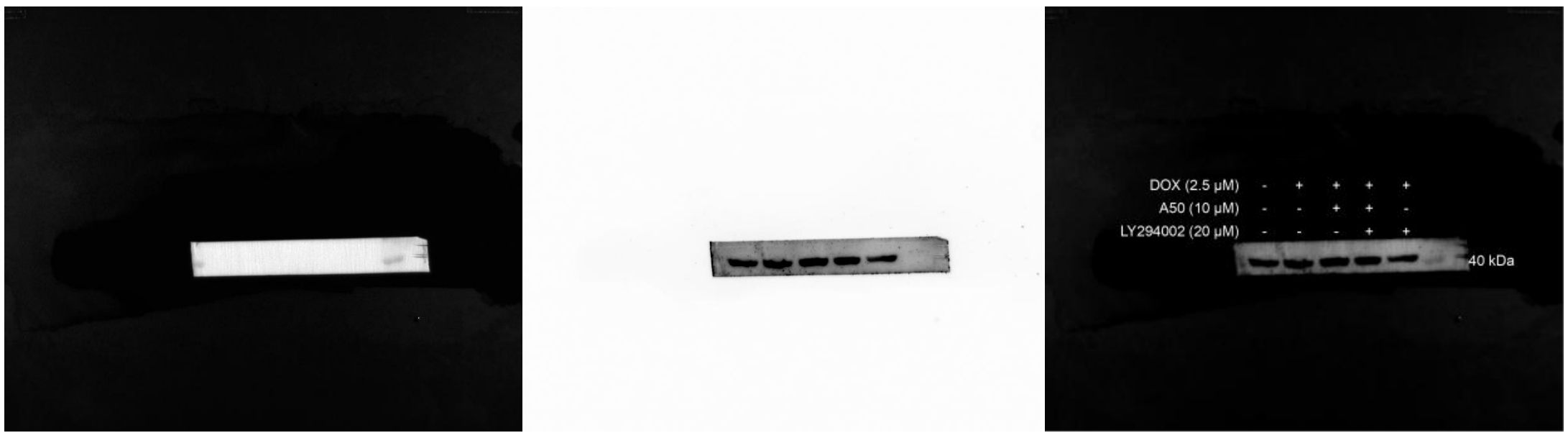

Full-size

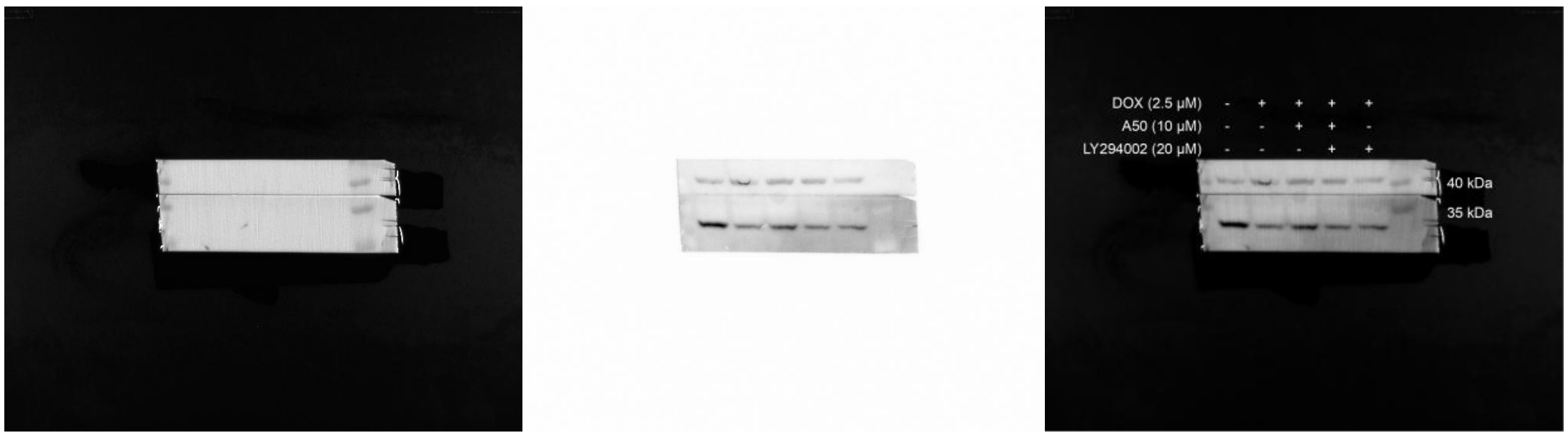

(2) HO-1

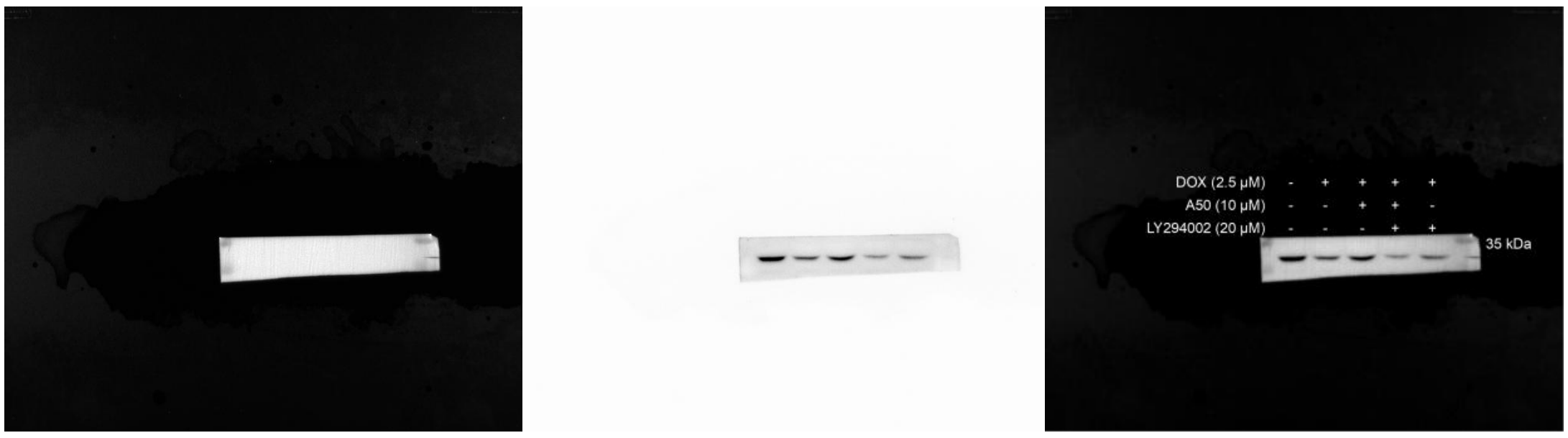

β-actin

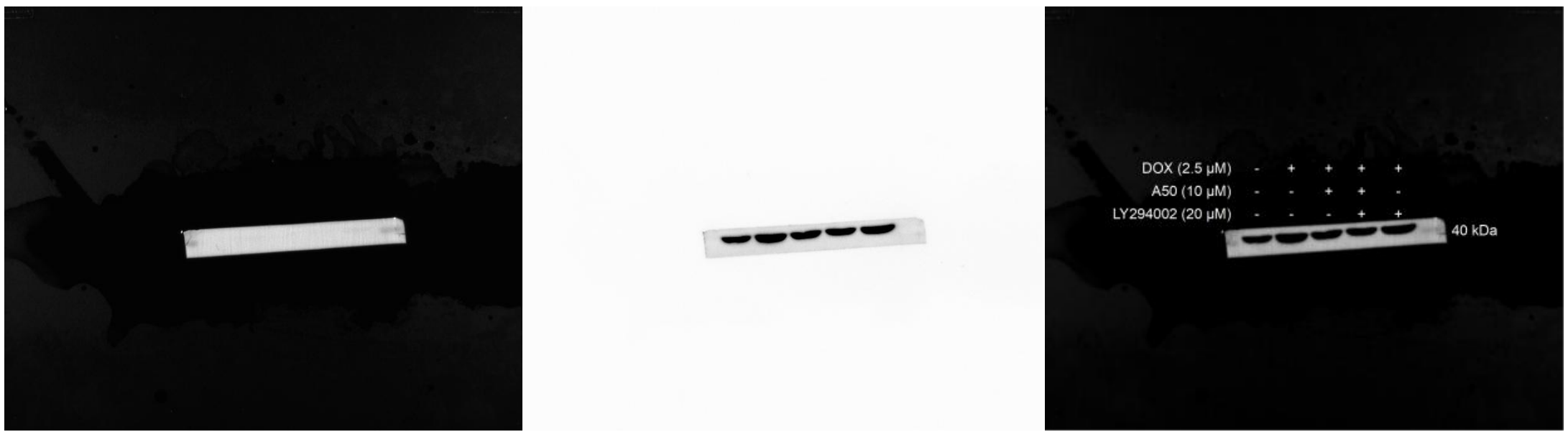

Full-size

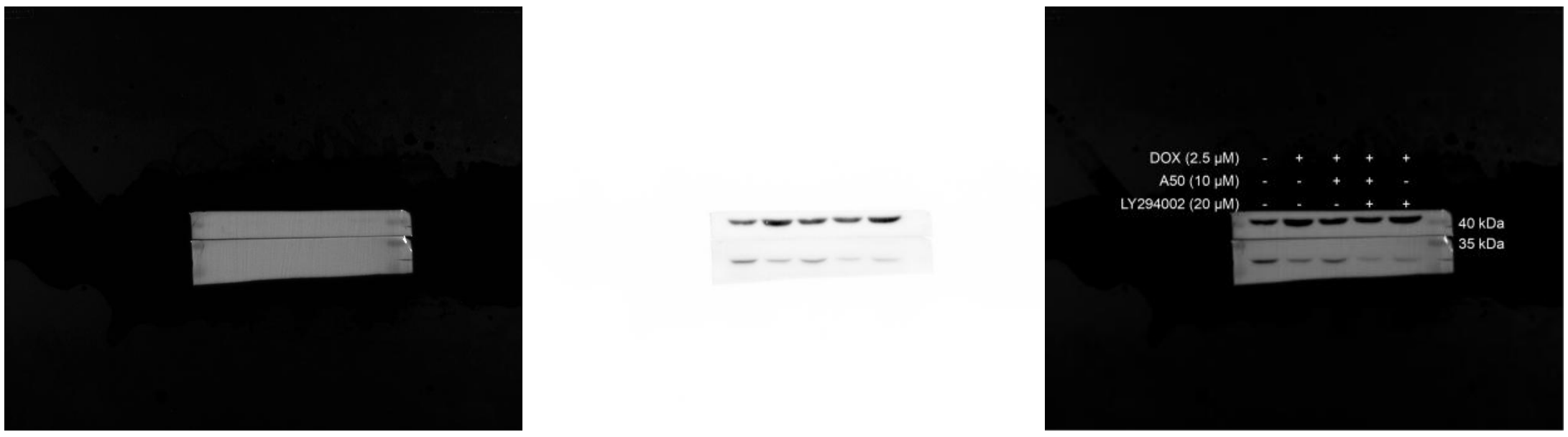

(3) HO-1

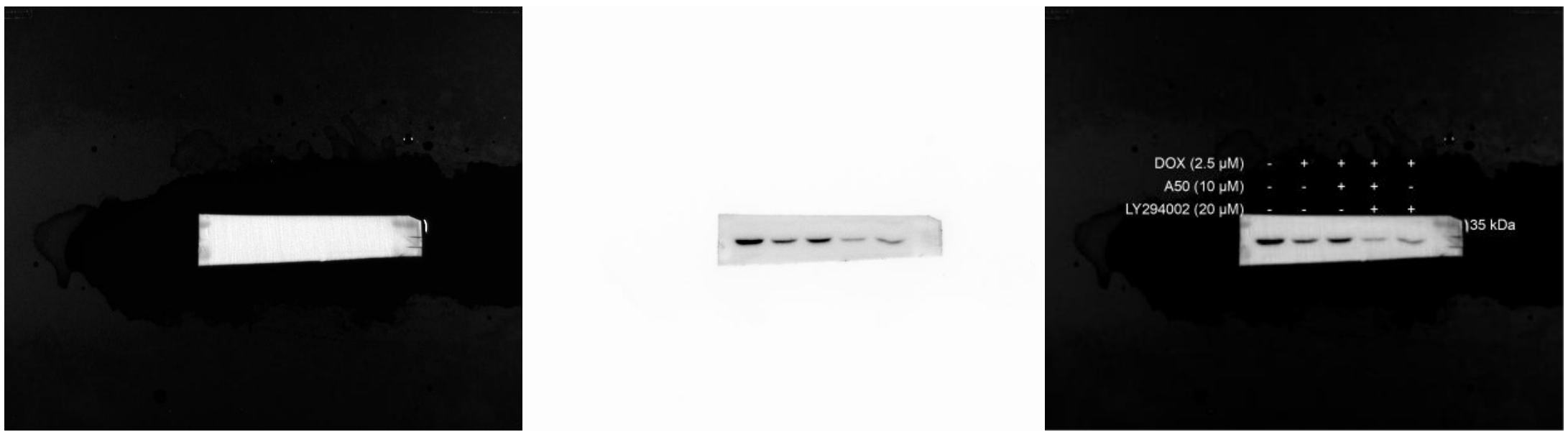

β-actin

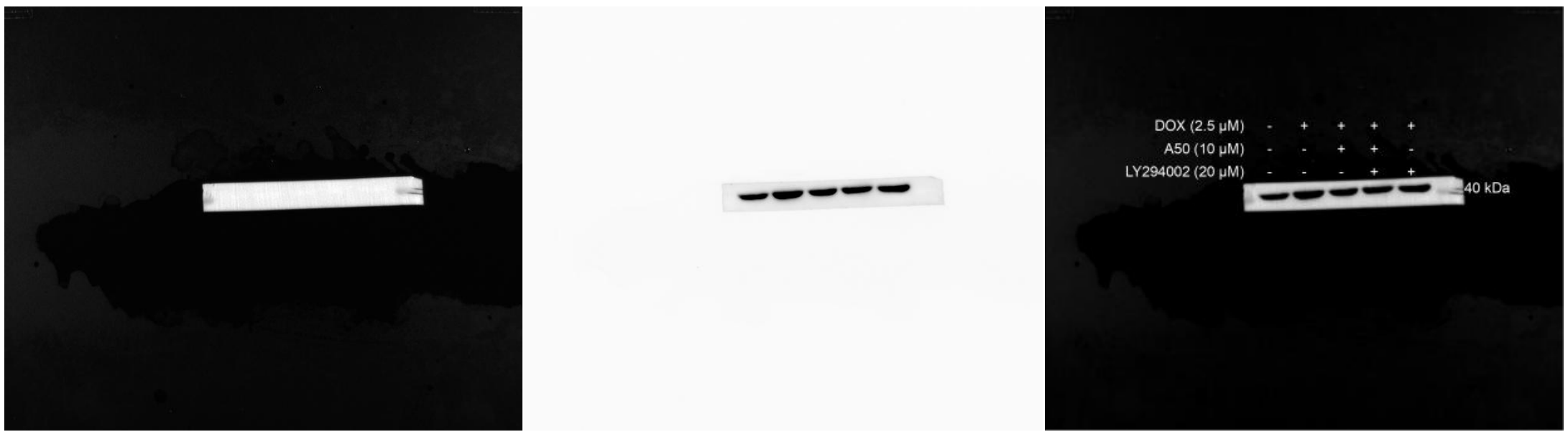

Full-size

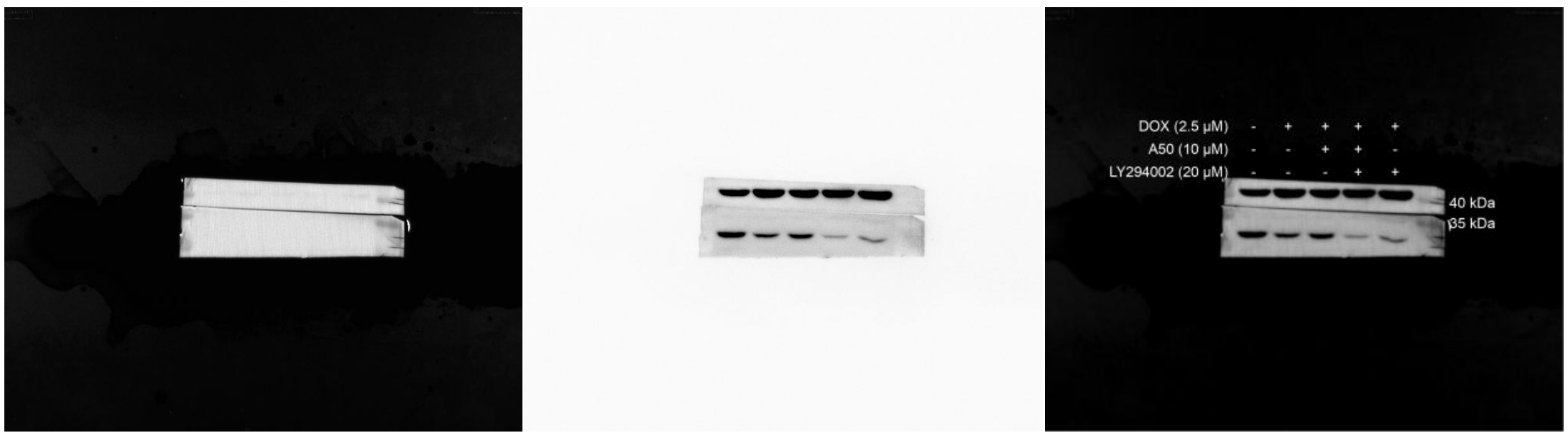

2. (1) PI3K

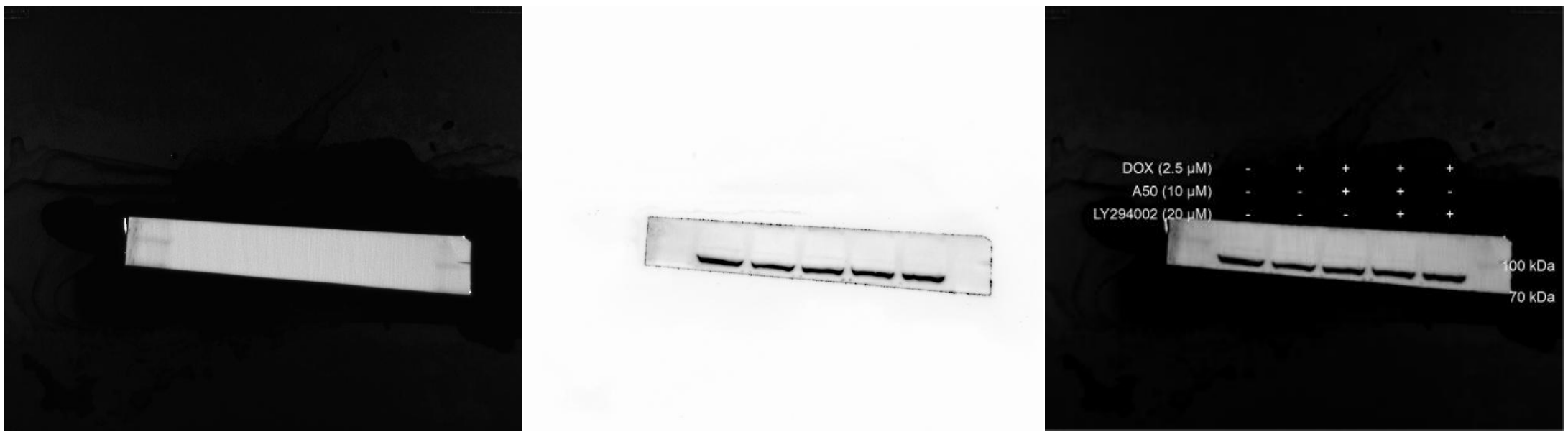

AKT

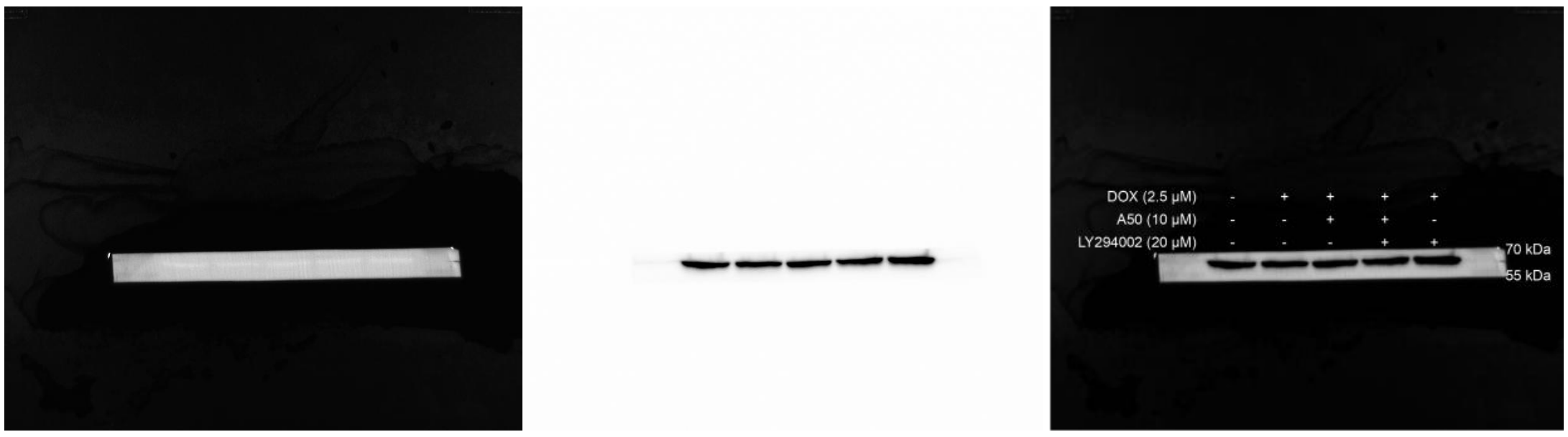

β-actin

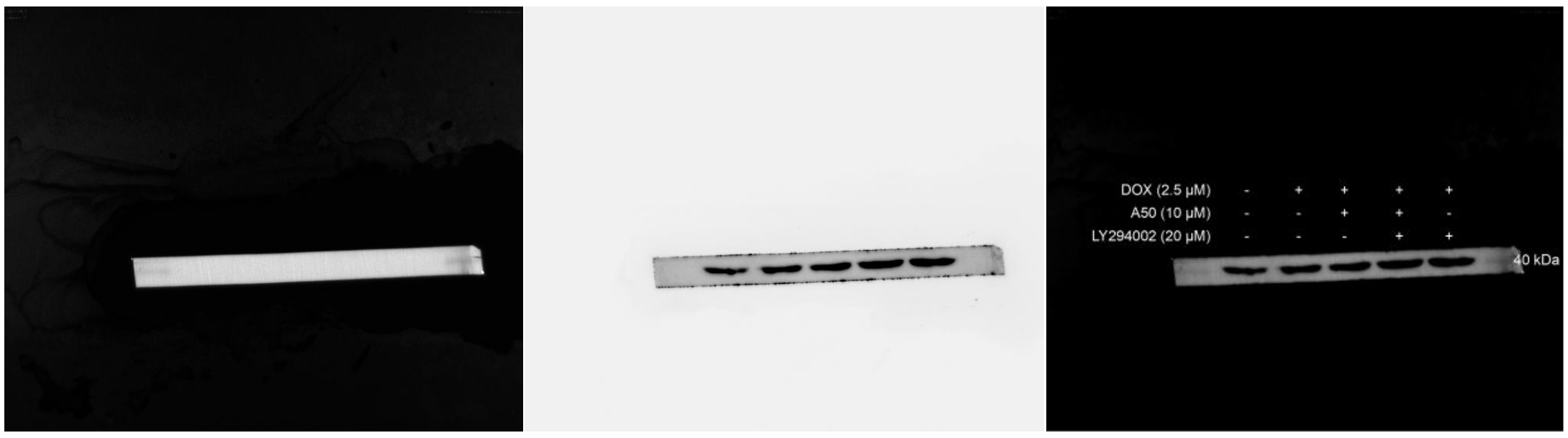

Full-size

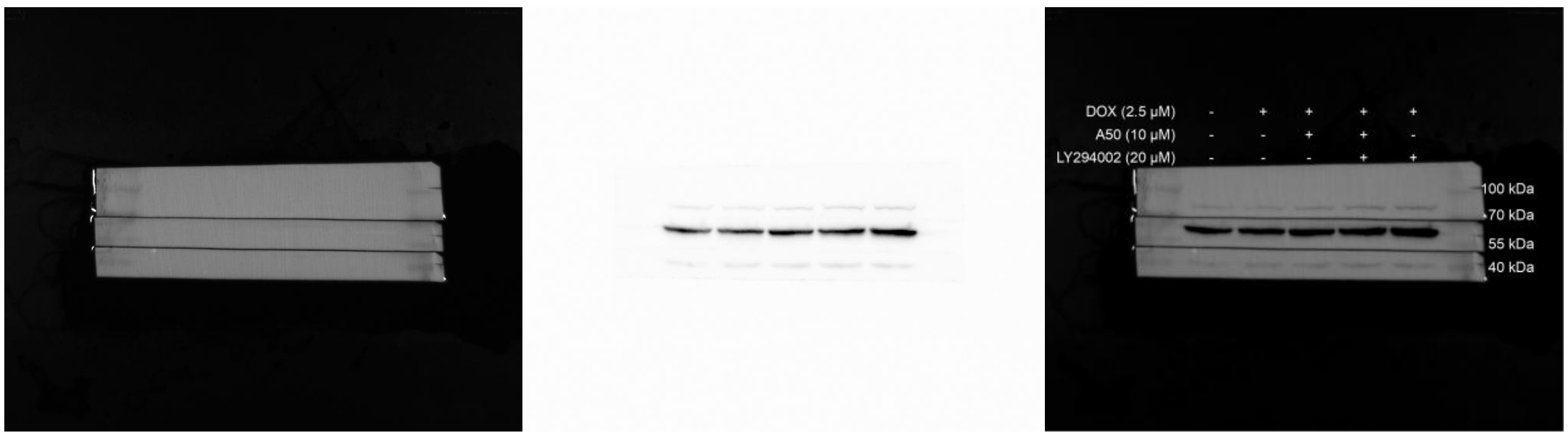

(2) PI3K

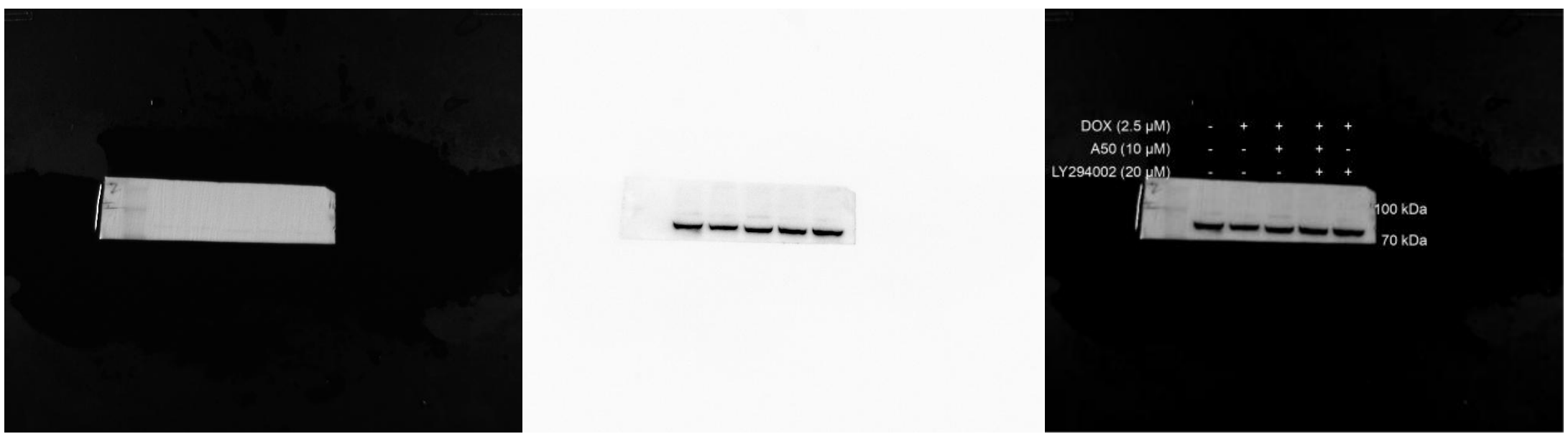

AKT

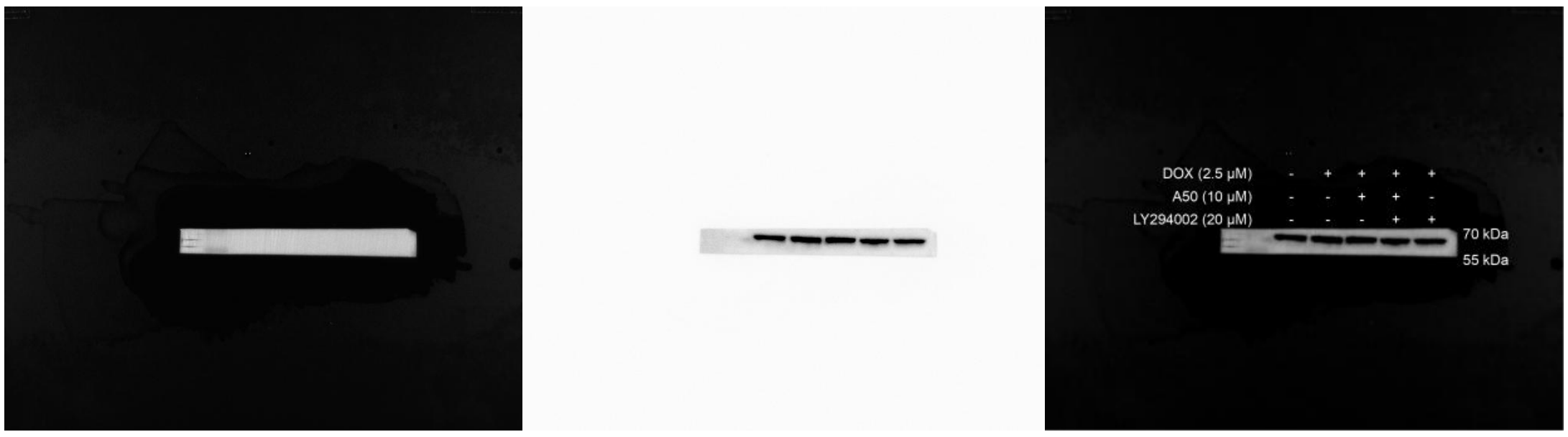

β-actin

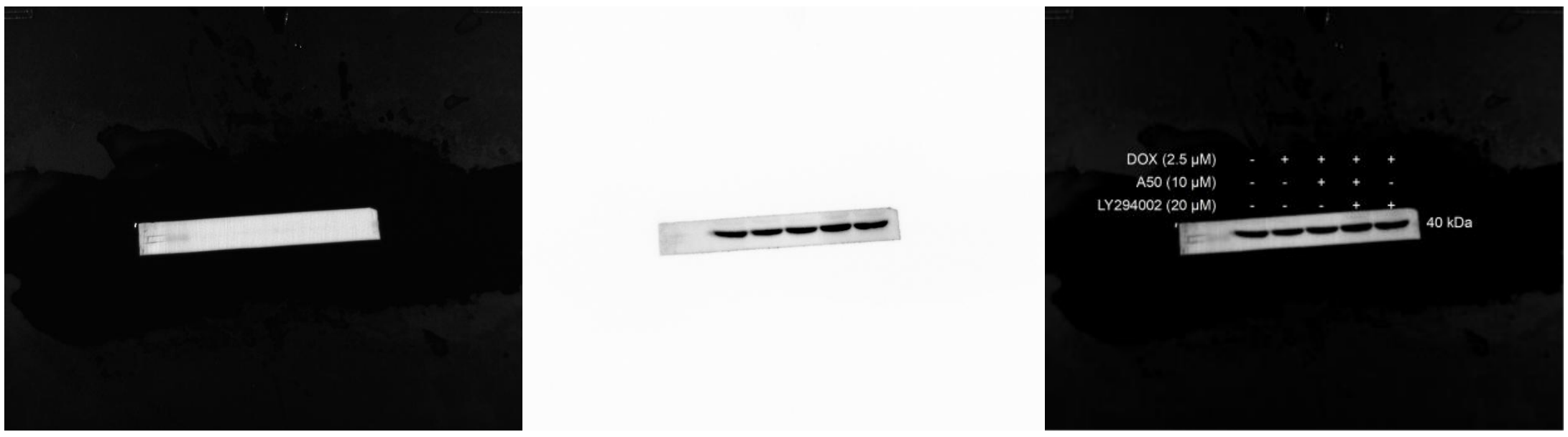

Full-size

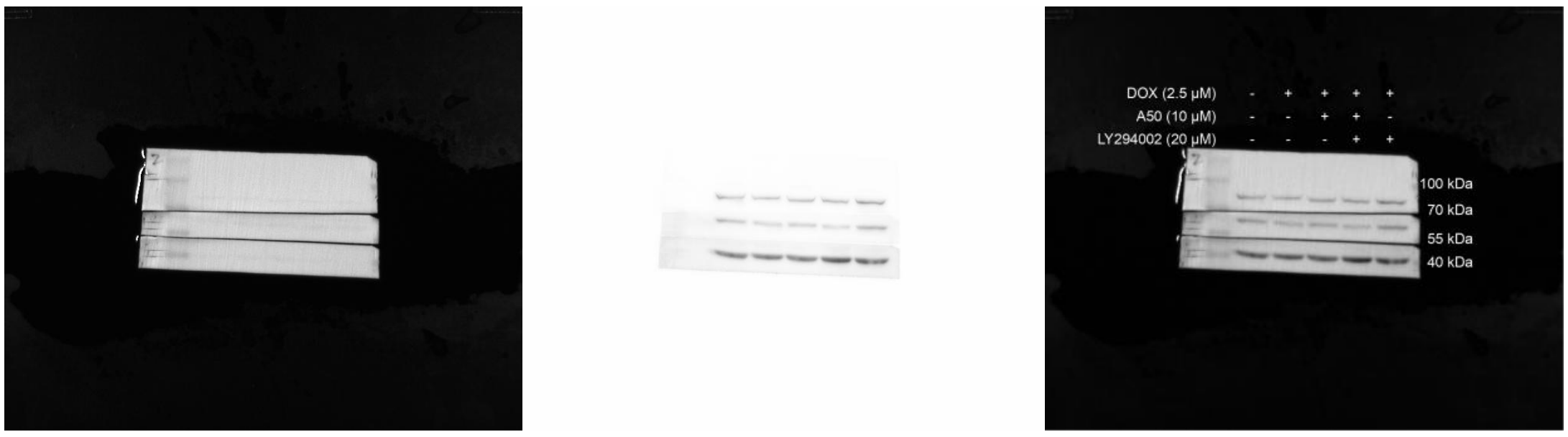

(3) PI3K

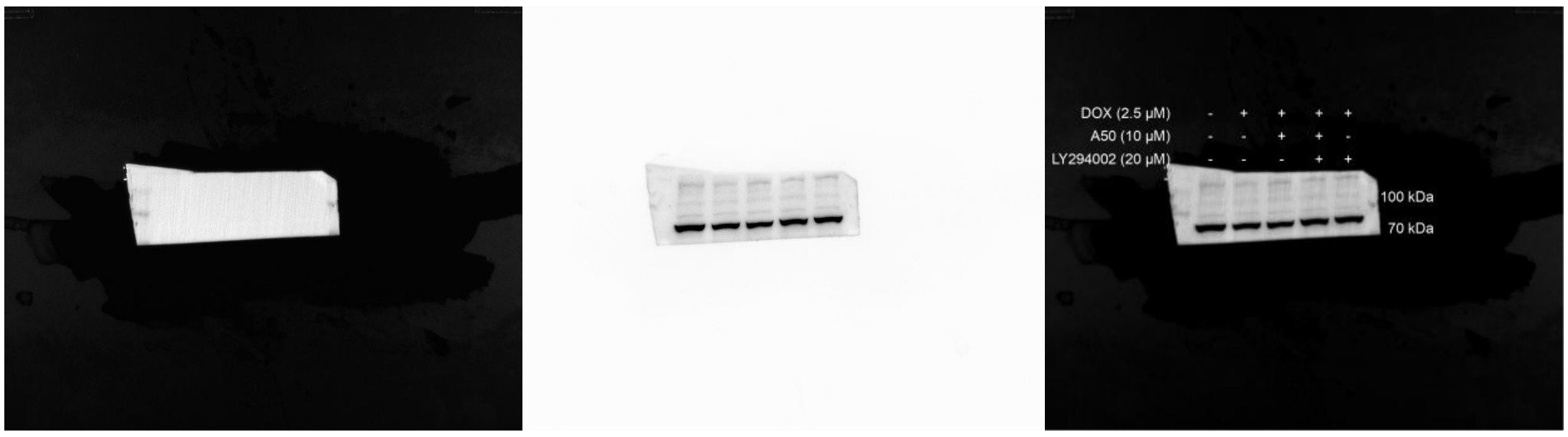

AKT

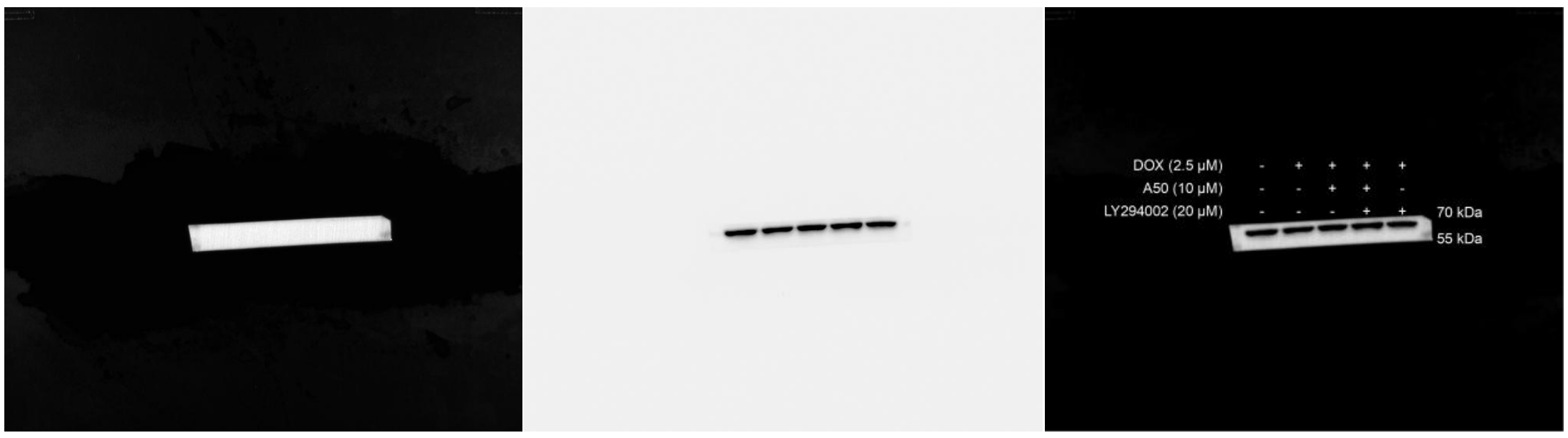

β-actin

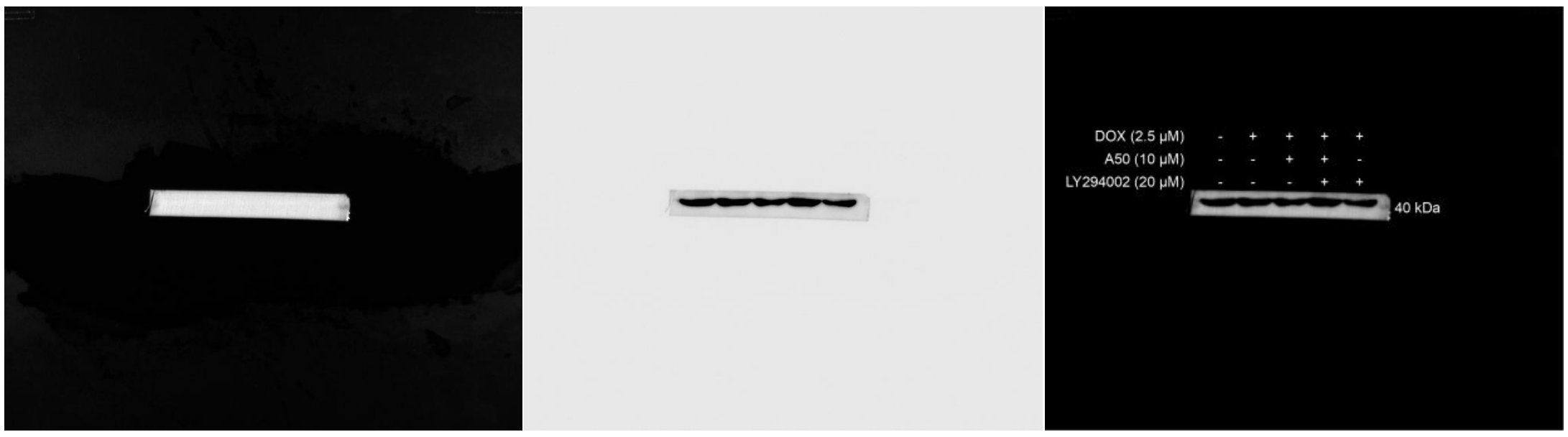

Full-size

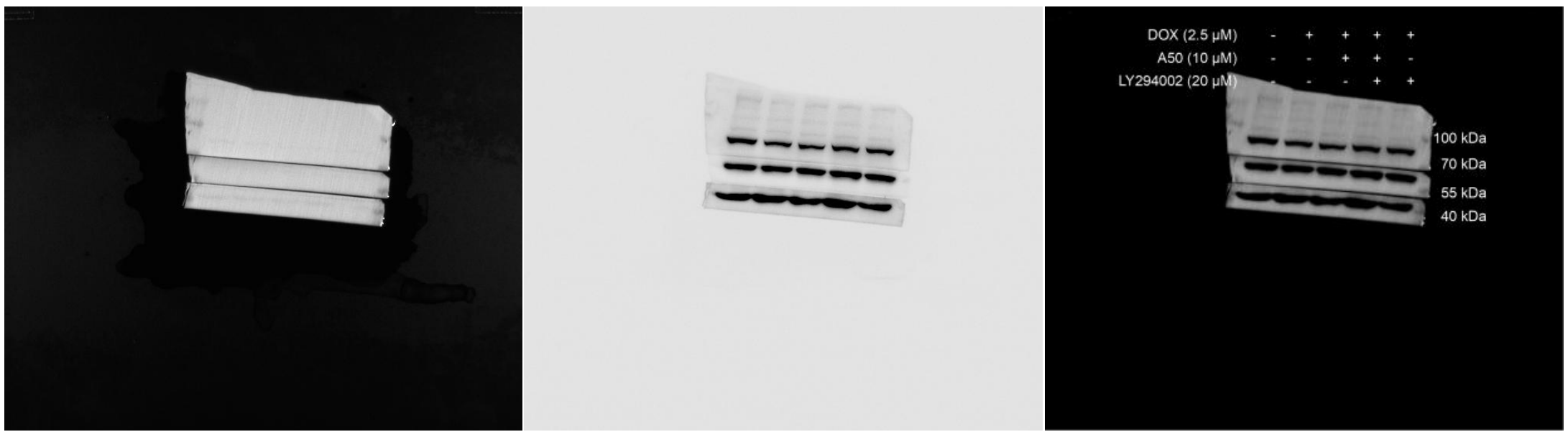

3. (1) p-PI3K

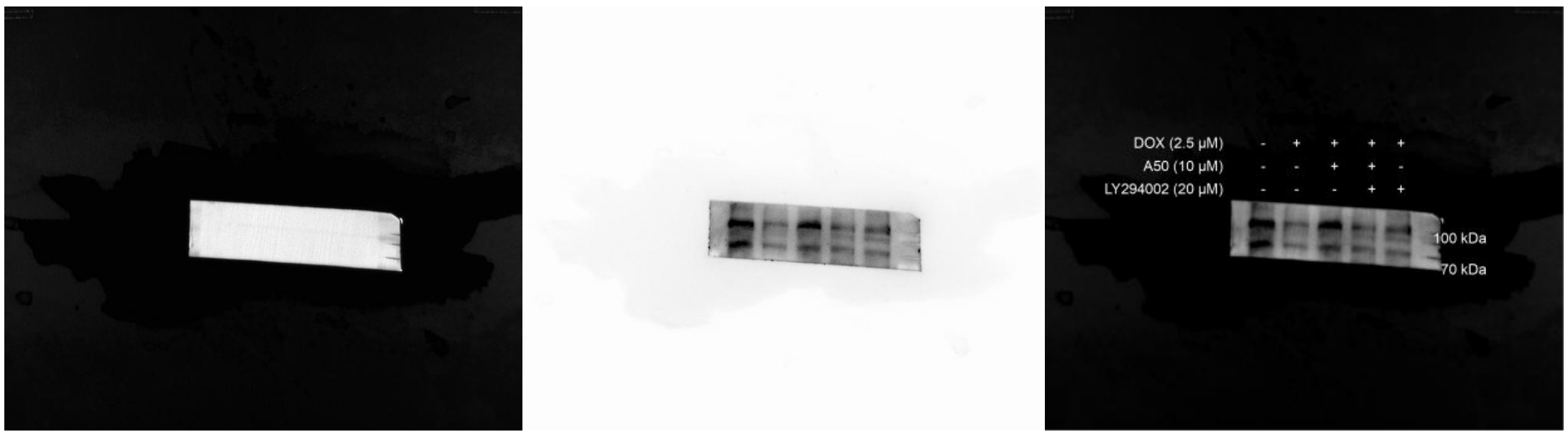

β-actin

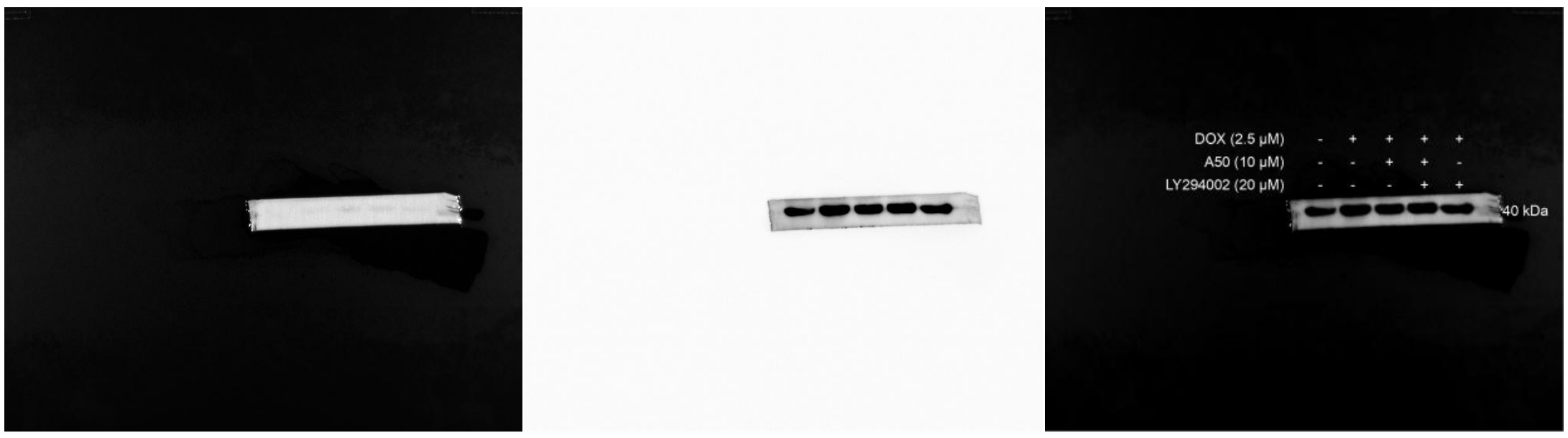

Full-size

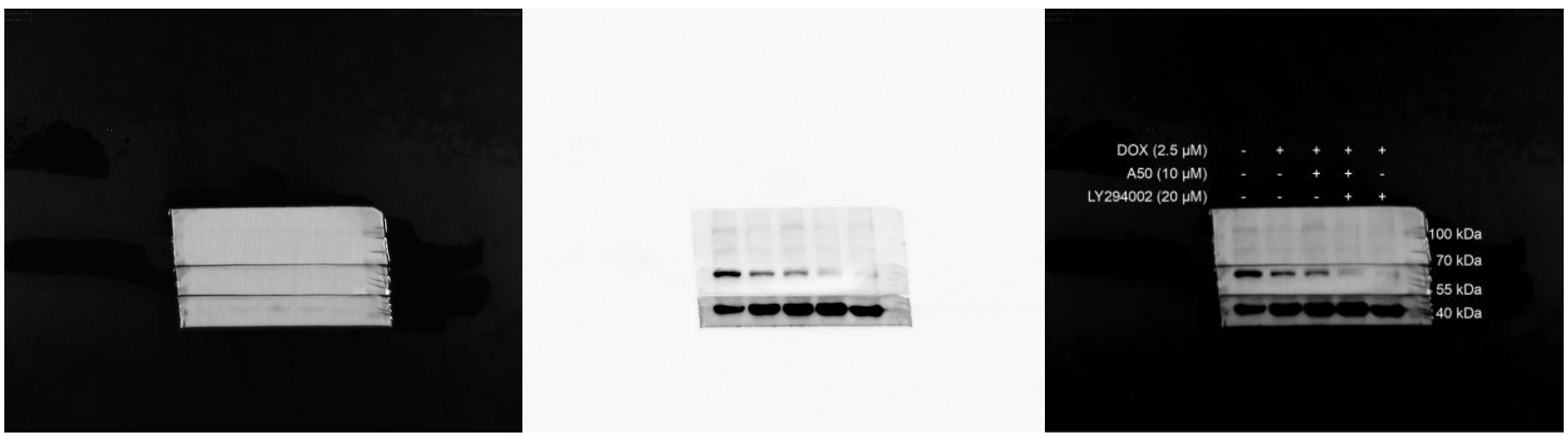

(2) p-PI3K

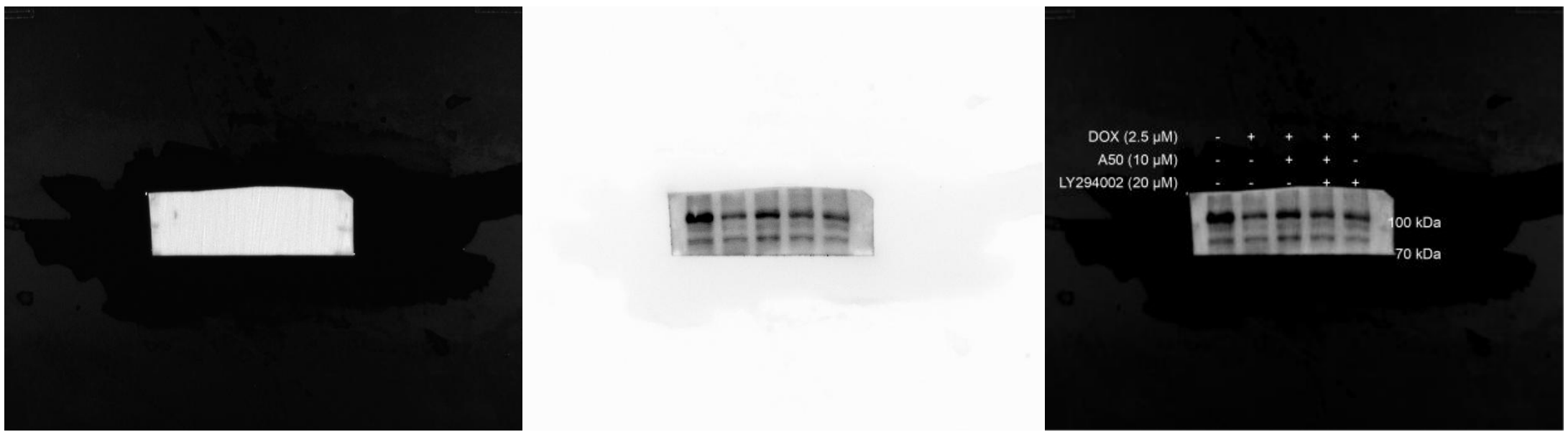

β-actin

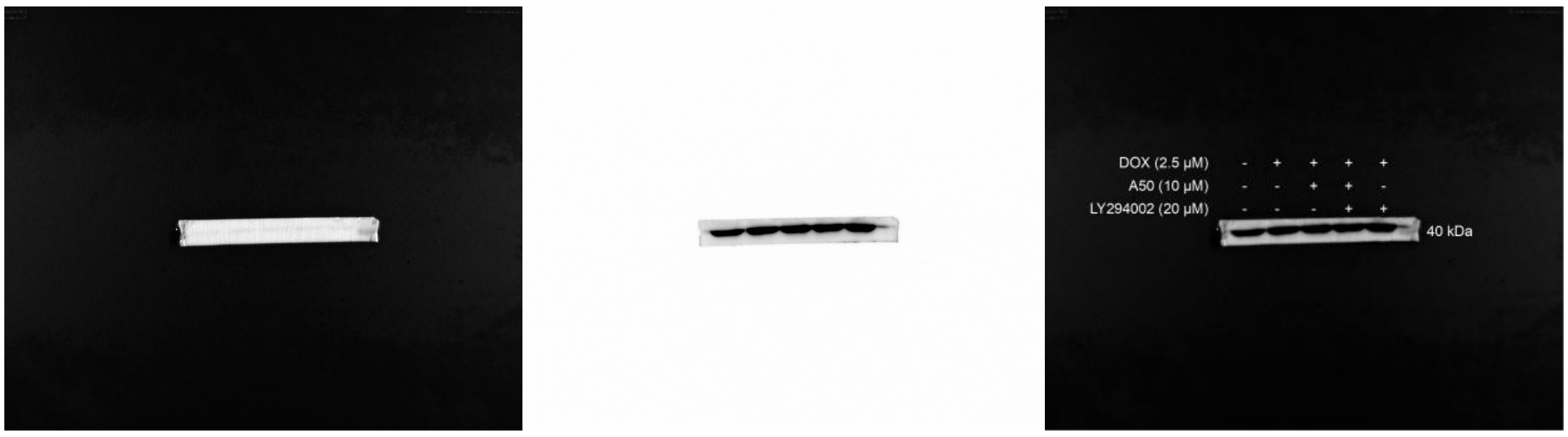

Full-size

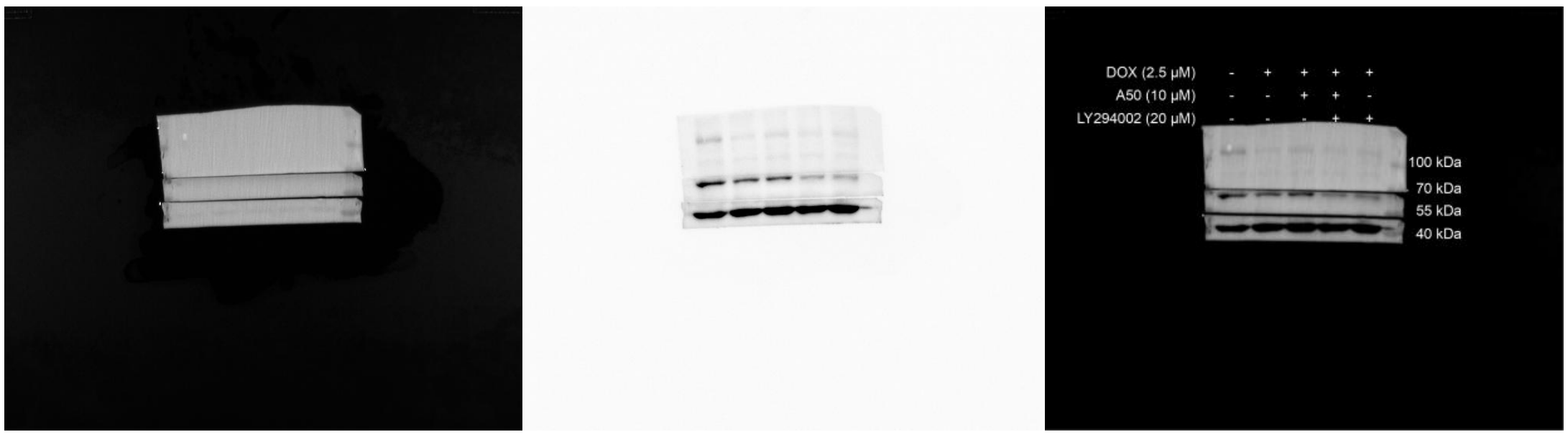

(3) p-PI3K

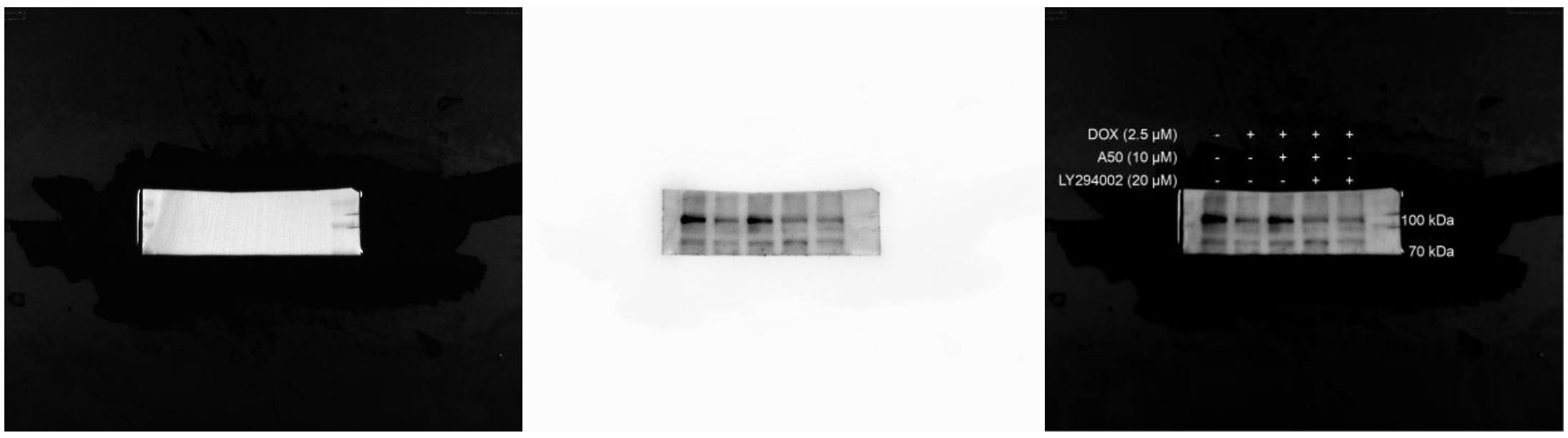

β-actin

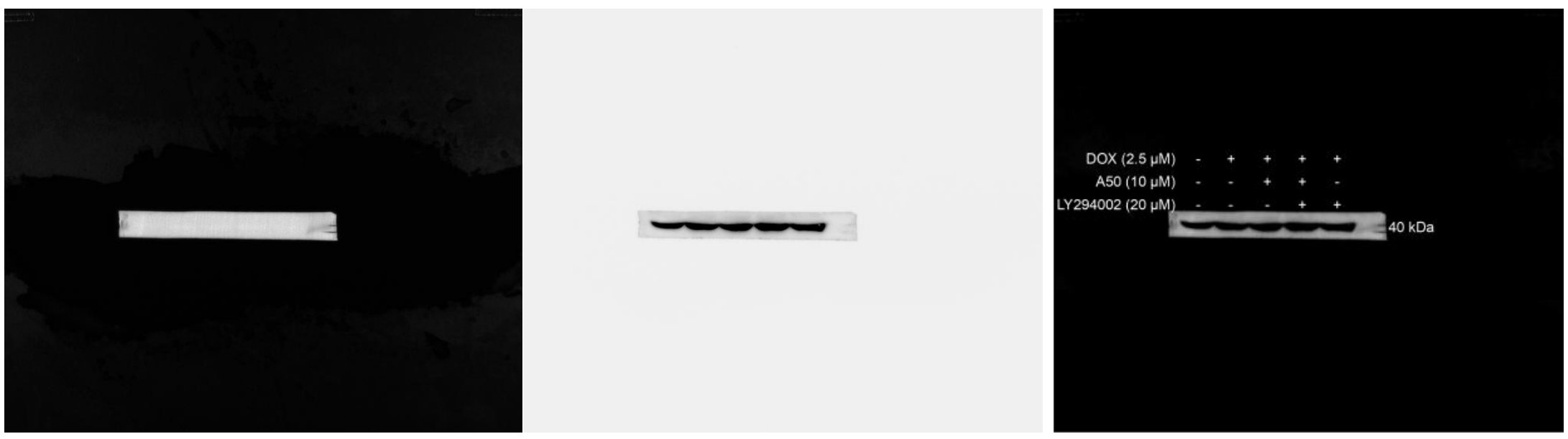

Full-size

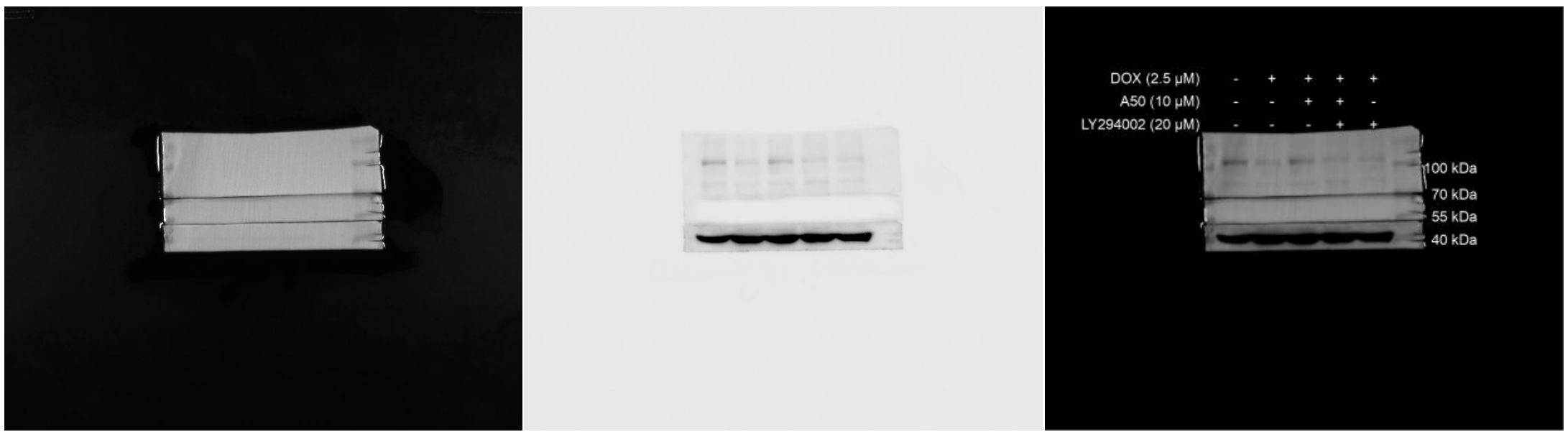

4. (1) p-AKT

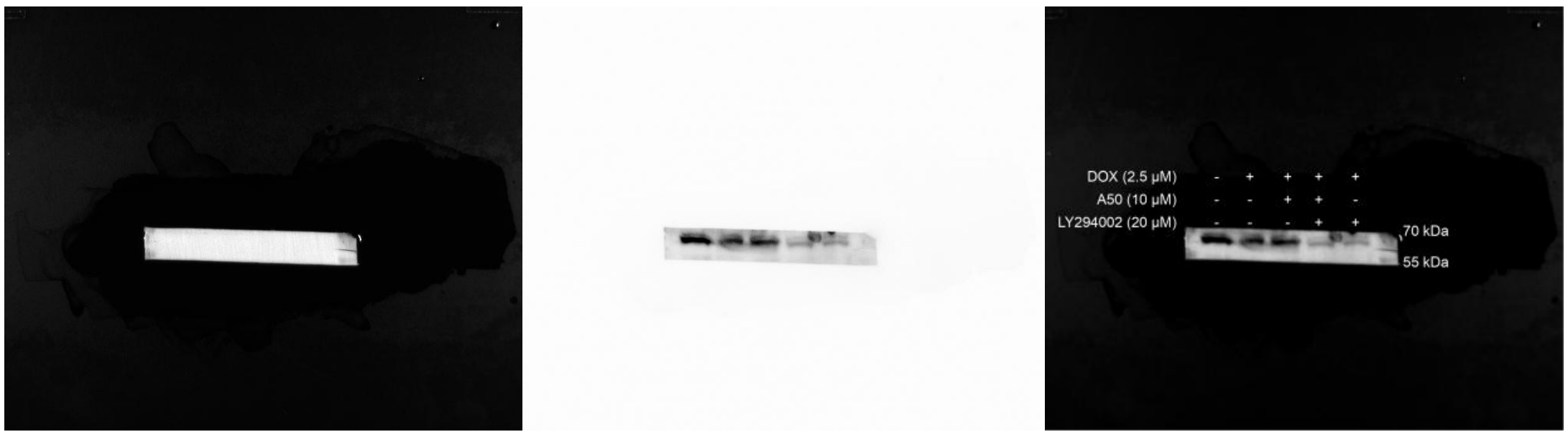

β-actin

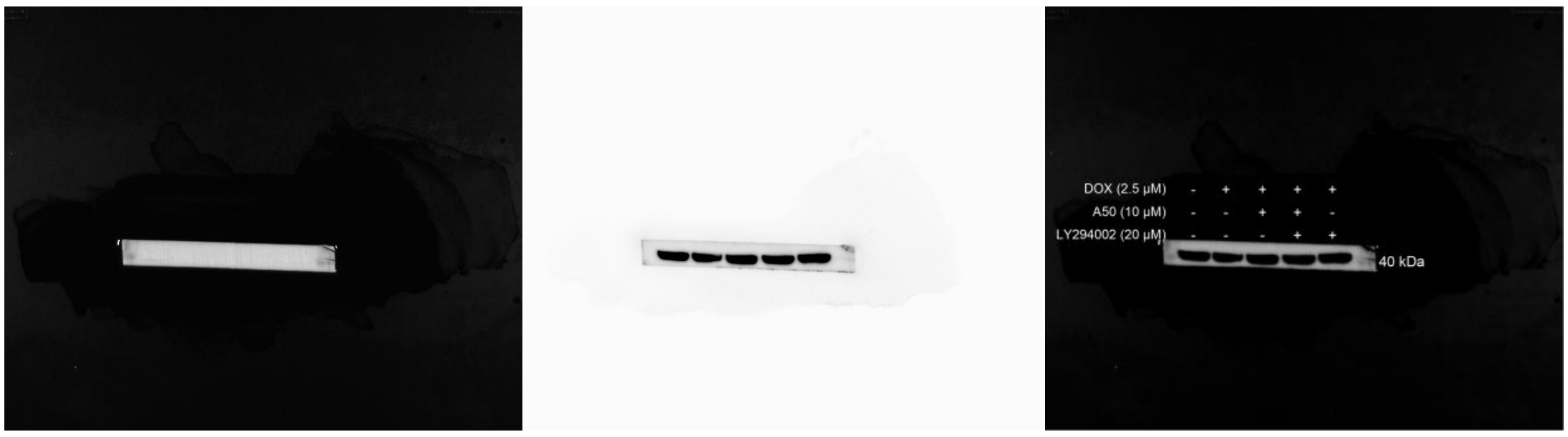

Full-size

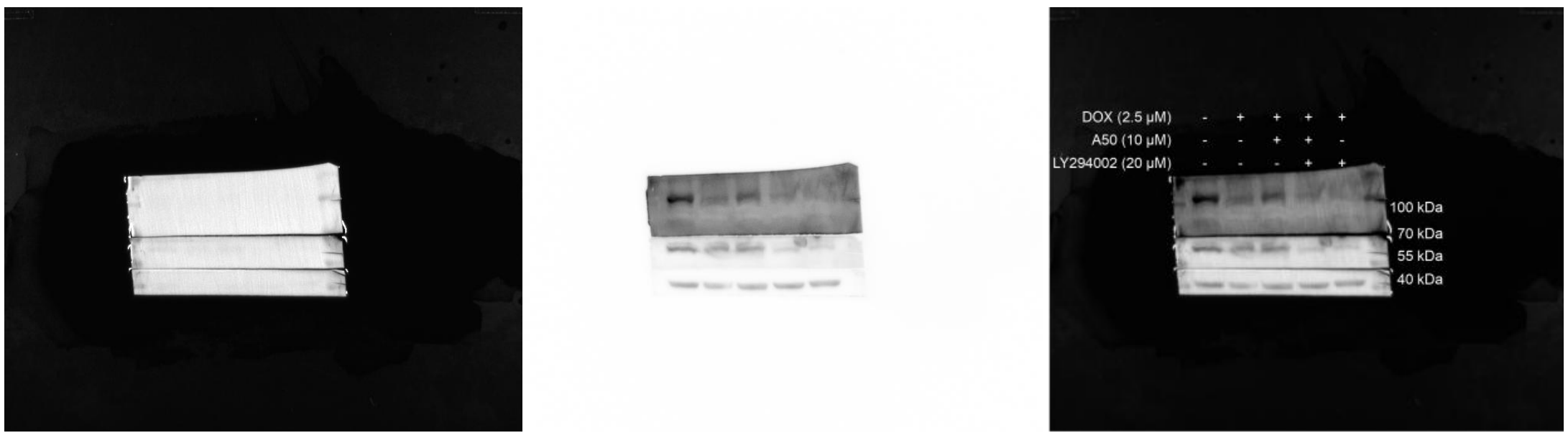

(2) p-AKT

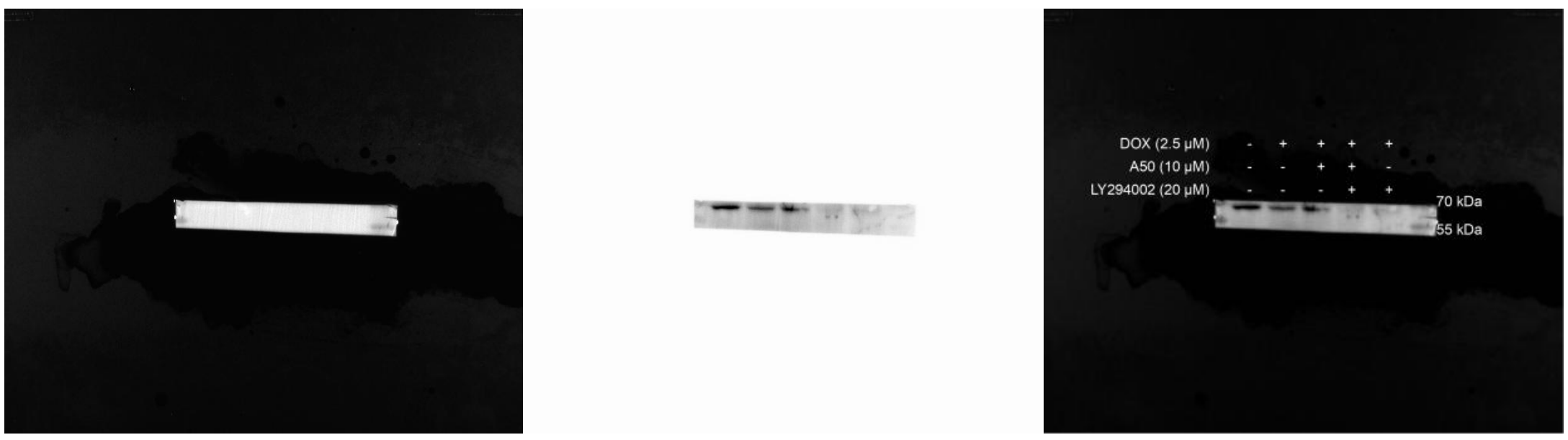

β-actin

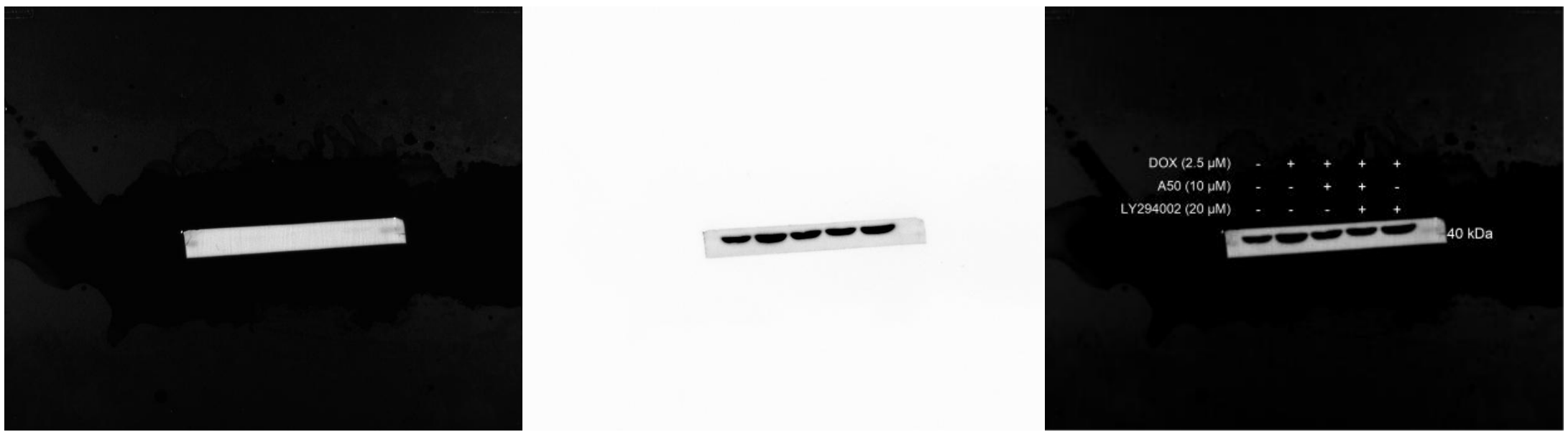

Full-size

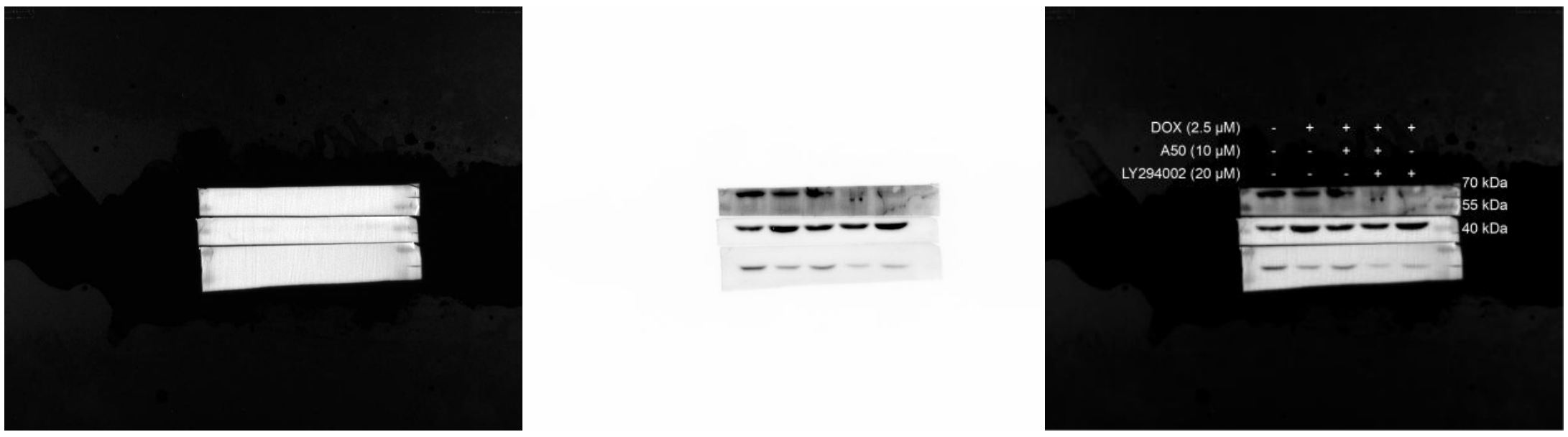

(3) p-AKT

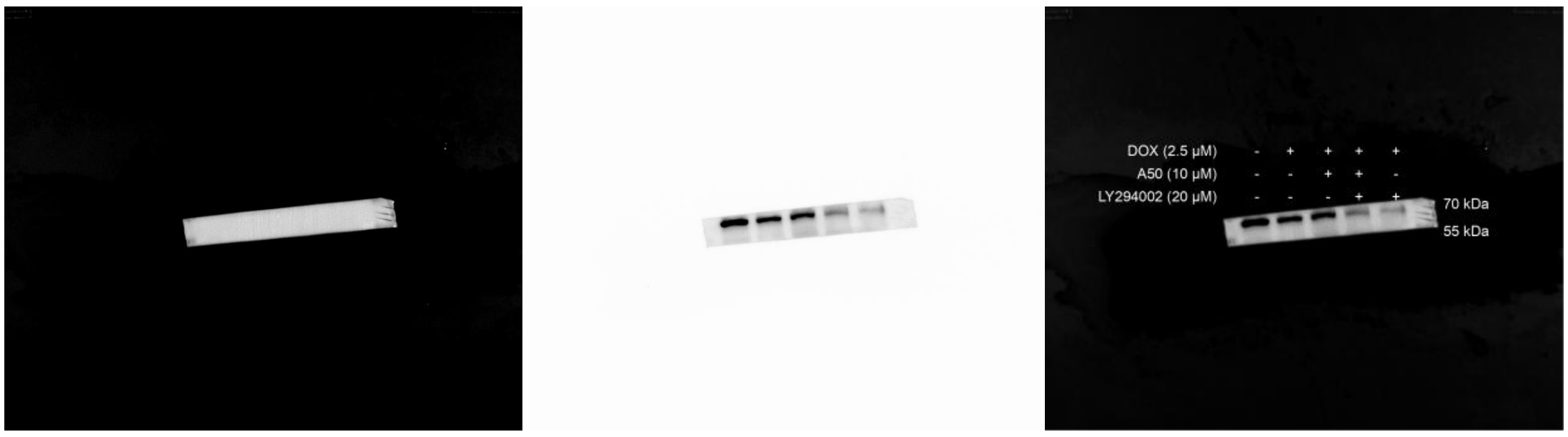

β-actin

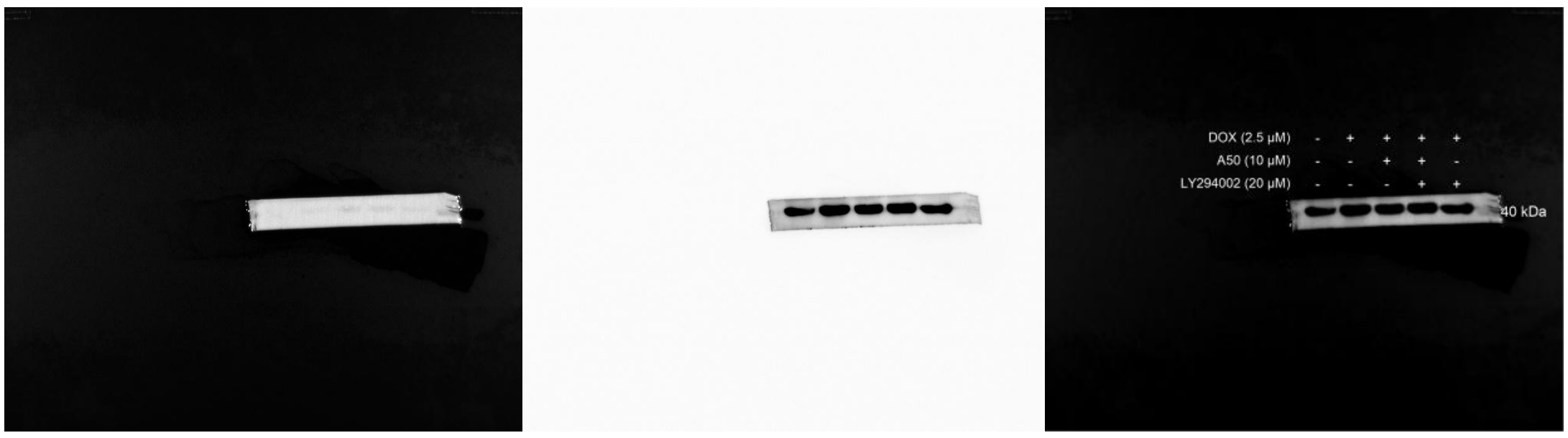

Full-size

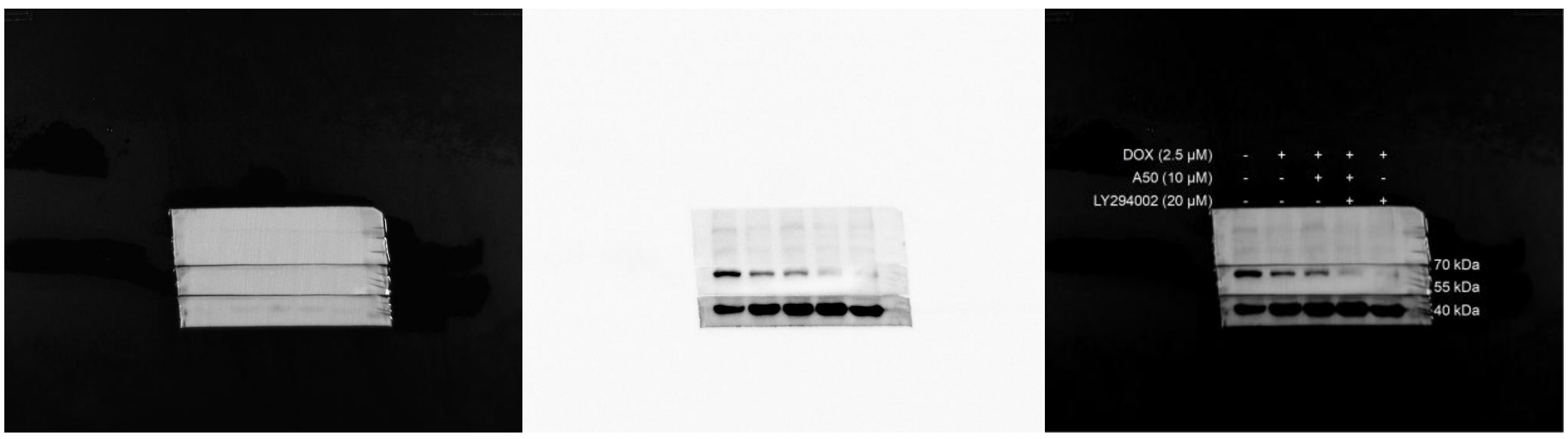

Supplement: Supplementary file 1 [file DataSheet1.pdf]
